# Supplementary material for: Ubiquitination of CLIP-170 family protein restrains polarized growth upon DNA replication stress
Source: Nat Commun. 2022 Sep 22;13:5565. doi: 10.1038/s41467-022-33311-y (PMC9499959; doi:10.1038/s41467-022-33311-y)
Supplement: Supplementary file 1 — Supplementary Information [file 41467_2022_33311_MOESM1_ESM.pdf]

Supplementary Information

Ubiquitination of CLIP-170 family protein restrains polarized growth upon DNA replication stress

Xi Wang[1#], Fan Zheng[2#], Yuan-yuan Yi[1], Gao-yuan Wang[1], Li-xin Hong[3], Dannel McCollum[4], Chuanhai Fu[2]*, Yamei Wang[1]*, Quan-wen Jin[1]*

*Corresponding Authors: Chuanhai Fu (chuanhai@ustc.edu.cn), Yamei Wang (wangyamei@xmu.edu.cn), and Quan-wen Jin (jinquanwen@xmu.edu.cn).

This PDF file includes:

Supplementary Figures 1 to 14

Supplementary Tables 1 to 3

Supplementary References

a

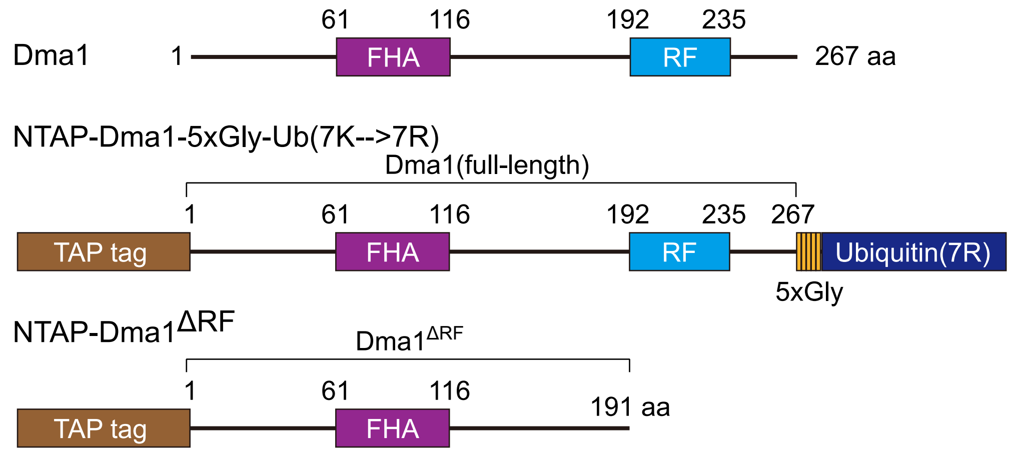

b

Proteins identified (#unique peptides; %coverage)

dma1Δ + pREP41-NTAP-Dma1-5xGly-Ub(7K-->7R)	*mts3-1 dnt1Δ dma1Δ* + pREP41-NTAP-Dma1-5xGly-Ub(7K-->7R)
Dma1 (26; 48.3%)	Dma1 (6; 28.1%)
Cka1 (6; 19.3%)	Nup124 (12; 22.8%)
Ckb1 (4; 15.2%)	Tea2 (7; 18.6%)
Tip1 (4; 10.0%)	Pob1 (6; 17.3%)

mts3-1 dnt1Δ dma1Δ + pREP41-NTAP-Dma1^{ΔRF}	*mts3-1 dnt1Δ dma1Δ* + pREP41-NTAP-Dma1-5xGly-Ub(7K-->7R) (continued)
Dma1 (37; 37.5%)	Tea3 (9; 16.3%)
Tip1 (9; 21.7%)	Gef1 (10; 16.3%)
	Sec13 (3; 16.2%)
	Lsb4 (3; 16.0%)
	Tea4 (6; 15.6%)
	Tip1 (4; 15.6%)

c

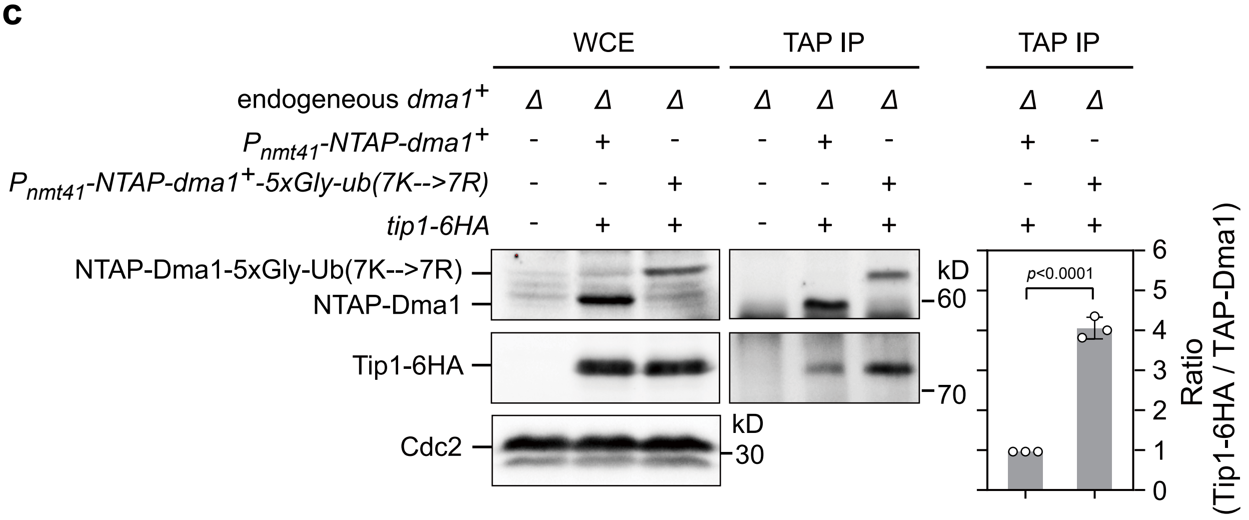

Supplementary Figure 1. Tip1 was identified as one of the Dma1-interacting proteins in TAP-Dma1 purifications.

(**a**) Schematic diagrams of constructs (not on scale) of Dma1 variants fused to TAP tag at N-terminus or mutant version of ubiquitin (ubiquitin-7R) at C-terminus, which were expressed in fission yeast cells and used for purifications of potential interacting proteins.

(**b**) Tandem mass spectrometry analyses of protein mixtures from three independent Dma1-TAP purifications identified Tip1 as one Dma1-interacting protein. Full lists of identified proteins from Dma1-TAP purifications are in Supplementary Data 1.

(**c**) Binding affinity between Dma1 and Tip1 is elevated by Dma1-5xGly-Ub(7K→7R) fusion. Yeast strains carrying *tip1-6HA* and P_{nmt41}*-NTAP-dma1*$^{+}$ or P_{nmt41}*-NTAP-dma1-5xGly-Ub(7K→R)* were grown in EMM5S without thiamine for 18 hours to induce expression of NTAP-Dma1 and NTAP-Dma1-5xGly-Ub(7K→7R). Lysates were prepared from yeast cells, and then the association of Tip1 to NTAP-Dma1 and NTAP-Dma1-5xGly-Ub(7K→7R) was assessed by immunoprecipitation of NTAP-tagged Dma1 versions and Western blot. The amount of co-immunoprecipitated Tip1-6HA was normalized to those of total immunoprecipitated NTAP-Dma1 or NTAP-Dma1-5xGly-Ub(7K→7R) in each sample, with the relative ratio between Tip1-6HA and unmodified NTAP-Dma1 set as 1.0. Quantitative data are presented in graph as mean ± SD from three independent experiments; *p* values were determined by two-tailed unpaired *t*-test. Note that Dma1-5xGly-Ub(7KR) fusion co-imunoprecipitated significantly more Tip1 than unmodified Dma1 did. WCE, whole cell extract.

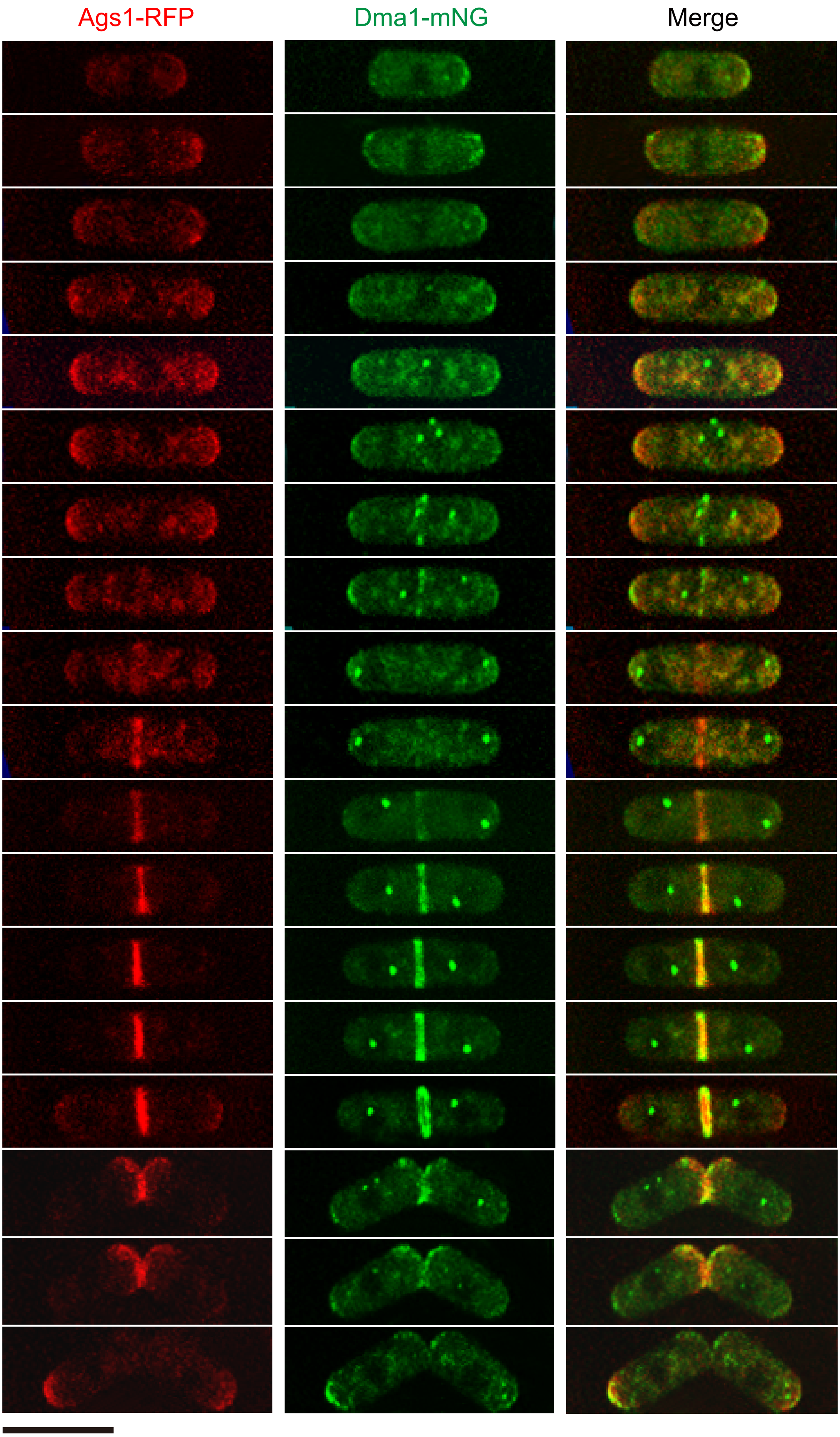
Ags1-RFP
Dma1-mNG
Merge

Supplementary Figure 2. Related to Figure 1d.

Enlarged and colour merged images of cells expressing both Dma1-mNeonGreen and Ags1-RFP. Scale bar, 10 μm.

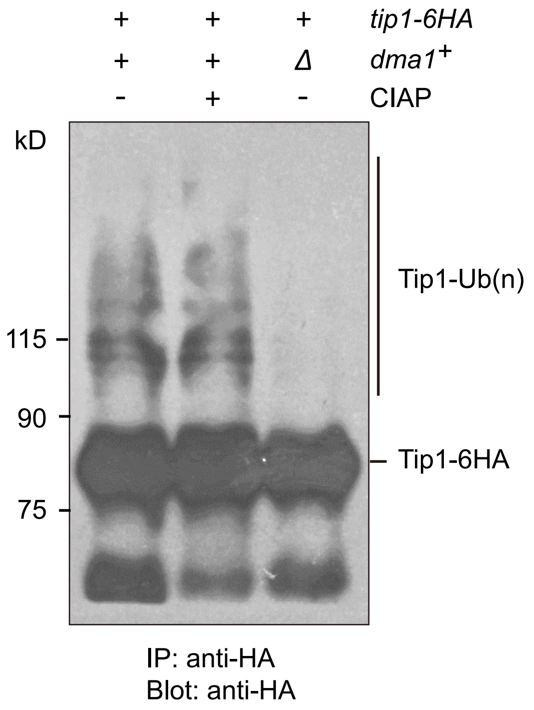
+ + + tip1-6HA
+ + Δ dma1+
- + - CIAP
kD
115
90
75
Tip1-Ub(n)
Tip1-6HA
IP: anti-HA
Blot: anti-HA

Supplementary Figure 3. The higher molecular weight forms of Tip1 is not derived from phosphorylation.

Cells of indicated strains carrying *tip1-6HA* grown in YE5S liquid medium at 30 ℃ were treated with 12 mM HU for 5.5 hr, collected, and then cell lysates were prepared and treated without or with CIAP (calf intestinal alkaline phosphatase) before immunoprecipitation with anti-HA antibodies. CIAP treatment did not remove the ladder of slow-migrating bands of immunoprecipitated Tip1-6HA.

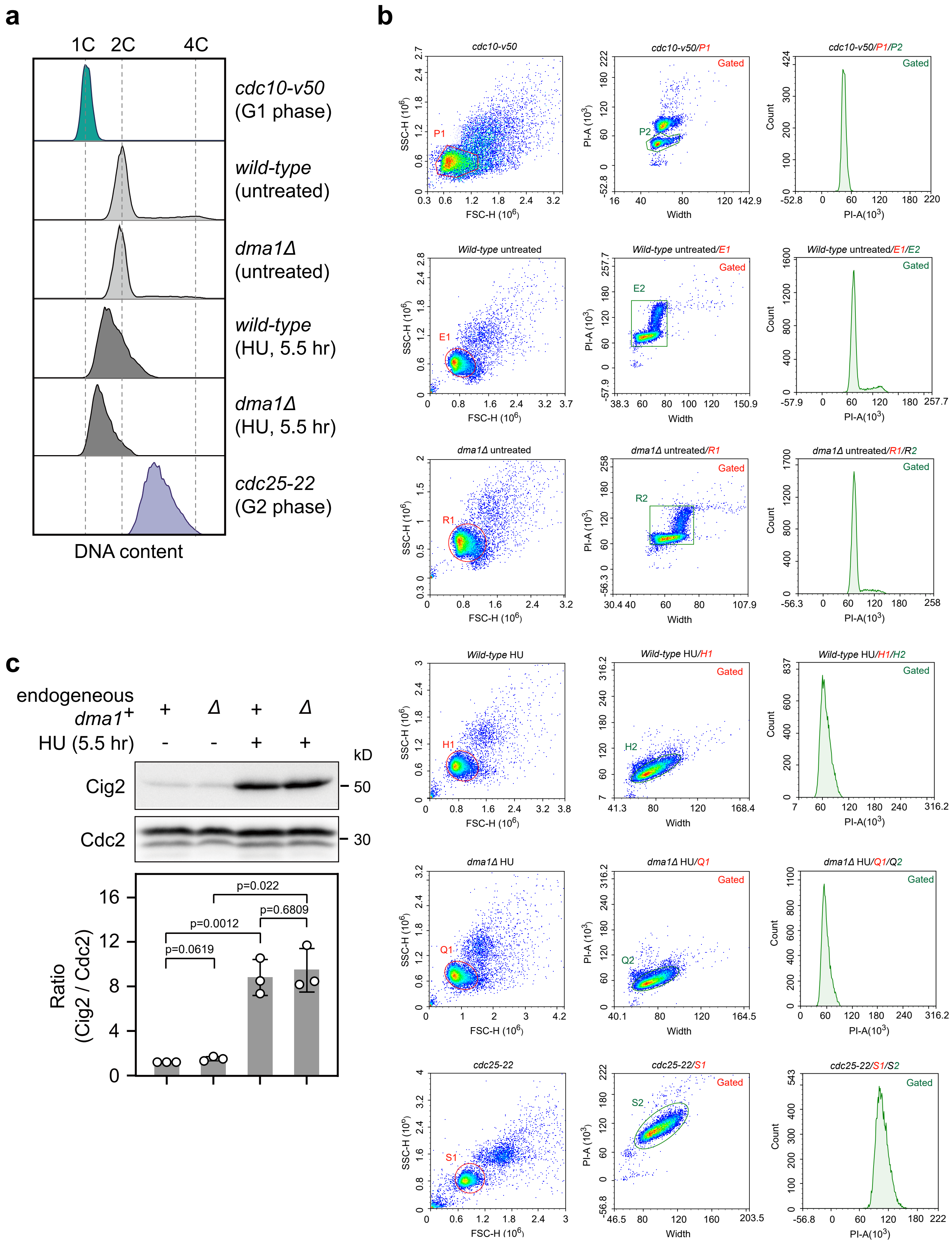

a
1C
2C
4C
cdc10-v50 (G1 phase)
wild-type (untreated)
dma1Δ (untreated)
wild-type (HU, 5.5 hr)
dma1Δ (HU, 5.5 hr)
cdc25-22 (G2 phase)
DNA content
b
cdc10-v50
cdc10-v50/P1
cdc10-v50/P1/P2
Gated
P1
P2
SSC-H (10^6)
FSC-H (10^6)
PI-A (10^3)
Width
Count
PI-A(10^3)
Wild-type untreated
Wild-type untreated/E1
Wild-type untreated/E1/E2
E1
E2
dma1Δ untreated
dma1Δ untreated/R1
dma1Δ untreated/R1/R2
R1
R2
Wild-type HU
Wild-type HU/H1
Wild-type HU/H1/H2
H1
H2
dma1Δ HU
dma1Δ HU/Q1
dma1Δ HU/Q1/Q2
Q1
Q2
cdc25-22
cdc25-22/S1
cdc25-22/S1/S2
S1
S2
c
endogeneous dma1+
+ Δ + Δ
HU (5.5 hr)
- - + +
kD
Cig2
Cdc2
50
30
p=0.022
p=0.6809
p=0.0012
p=0.0619
Ratio (Cig2 / Cdc2)
0
4
8
12
16

Supplementary Figure 4. ***dma1Δ*** **cells can be arrested at S phase by HU treatment as efficiently as** ***wild-type*** **cells.**

(**a**) DNA content was analyzed with fluorescence activated cell sorting (FACS). *Wild-type* or *dma1Δ* cells were grown in EMM5S and treated without or with 12 mM HU for 5.5 hr. *cdc10-v50* and *cdc25-22* cells were cultured at 25 ℃ and then shifted to 37 ℃ for 4 hr. Cells were collected, fixed with cold 70% ethanol, treated with RNase A and stained with propidium iodide (PI) before being analyzed on flow cytometer. The G_1 phase-arrested *cdc10-v50* cells had 1C DNA content, and the G_2/M phase-arrested *cdc25-22* cells had DNA content skewed to >2C, due to a large cell size vs. asynchronous (i.e. untreated with HU) *wild-type* and *dma1Δ* cells. Both HU-treated *wild-type* and *dma1Δ* cells stayed in the S phase and showed DNA content between 1C and 2C.

(**b**) Gating strategies are shown for (**a**).

(**c**) Levels of S phase cyclin Cig2 are comparable in HU-treated *wild-type* and *dma1Δ* cells. Lysates were prepared from *wild-type* or *dma1Δ* cells grown in EMM5S and treated without or with 12 mM HU for 5.5 hr. Samples were subjected to Western blot analyses using anti-Cig2 and anti-Cdc2 antibodies. Cig2 levels were normalized to those of total Cdc2 in each sample with the relative ratio between Cig2 and Cdc2 in untreated *wild-type* cells set as 1.0. Quantitative data are presented in graph as mean ± SD from three independent experiments; *p* values were determined by two-tailed unpaired *t*-test.

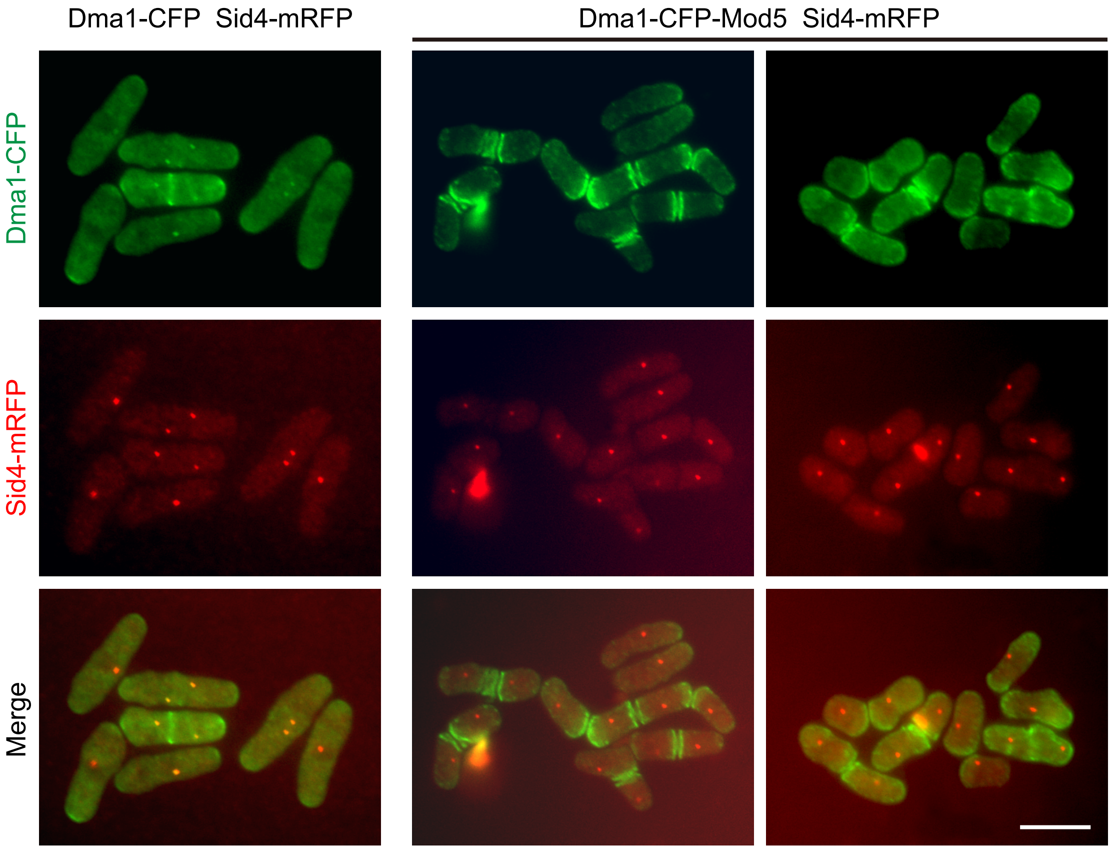

Dma1-CFP Sid4-mRFP
Dma1-CFP-Mod5 Sid4-mRFP
Dma1-CFP
Sid4-mRFP
Merge

Supplementary Figure 5. The engineered fusion protein Dma1-CFP-Mod5 strongly localizes at cell tip regions but not at SPBs.

Live cell images from yeast strains with indicated genotypes were captured under fluorescent microcopy. Sid4-mRFP was visualized as a reference for SPBs.

Scale bar, 10 μm.

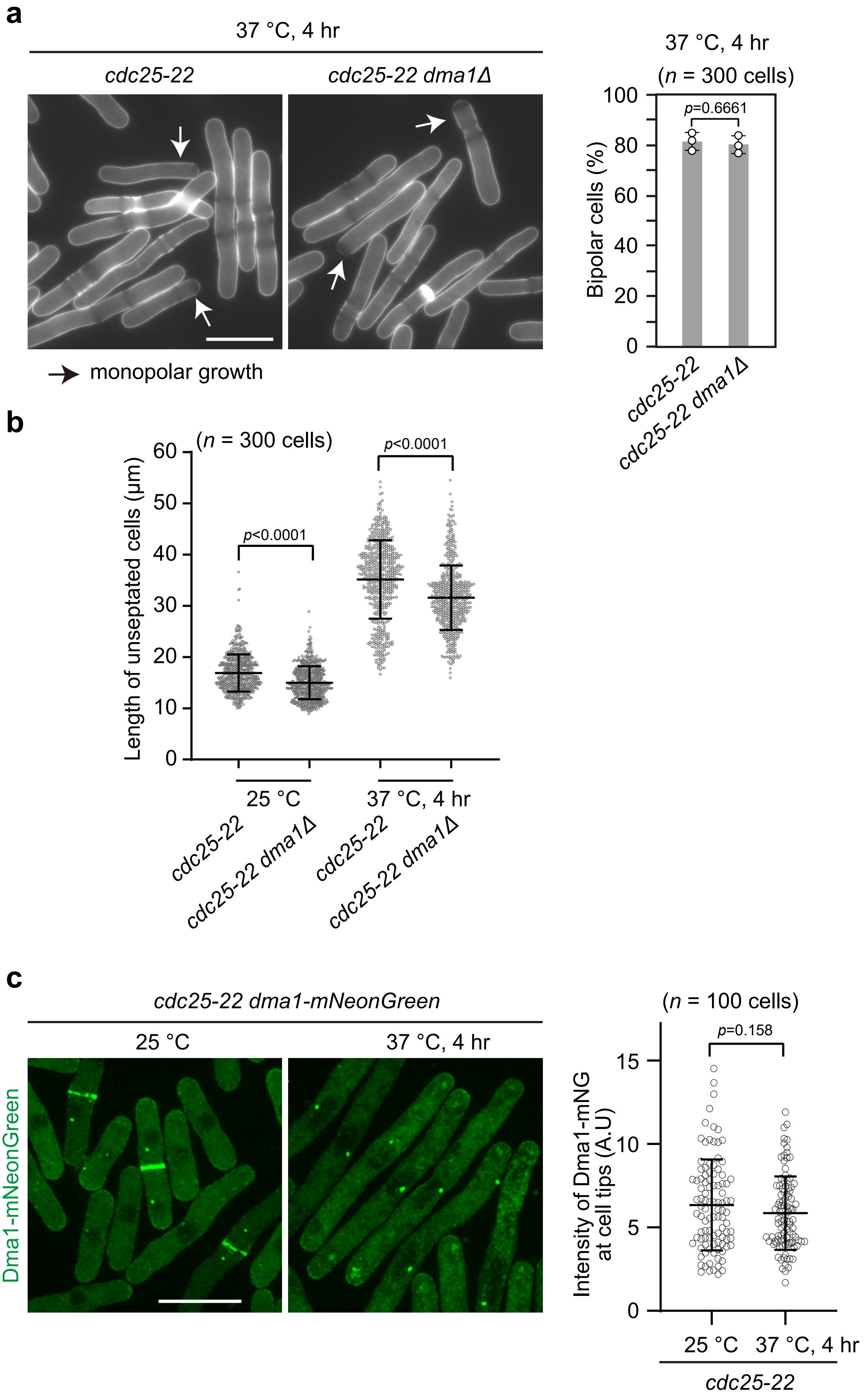

a
37 °C, 4 hr
cdc25-22
cdc25-22 dma1Δ
monopolar growth
37 °C, 4 hr
(n = 300 cells)
p=0.6661
Bipolar cells (%)
100
80
60
40
20
0
cdc25-22
cdc25-22 dma1Δ
b
(n = 300 cells)
p<0.0001
p<0.0001
Length of unseptated cells (μm)
60
50
40
30
20
10
0
25 °C
37 °C, 4 hr
cdc25-22
cdc25-22 dma1Δ
cdc25-22
cdc25-22 dma1Δ
c
cdc25-22 dma1-mNeonGreen
25 °C
37 °C, 4 hr
Dma1-mNeonGreen
(n = 100 cells)
p=0.158
Intensity of Dma1-mNG at cell tips (A.U)
15
10
5
0
25 °C
37 °C, 4 hr
cdc25-22

Supplementary Figure 6. Dma1 is not involved in inhibiting NETO.

(**a**) Deletion of *dma1*$^+$ does not accelerate NETO. The yeast strains with indicated mutations were either cultured at 25 ℃ or shifted from 25 ℃ to 37 ℃ for 4 hours. Cells were collected, fixed and stained with calcofluor (*Left*). Polar growth pattern was scored (*Right*). Arrows indicate monopolar cells. Note that *cdc25-22* mutants were arrested as bipolar (after NETO) cells at 37 ℃. The experiment was replicated three times.

(**b**) Cell length is not increased in *dma1Δ* cells at NETO. Cells were grown and arrested as in (**a**). Live images were captured and cell length of unseptated cells was measured.

(**c**) NETO does not promote accumulation of Dma1 at cell ends. Yeast strain of *cdc25-22 dma1-mNeonGreen* was either cultured at 25 ℃ or shifted from 25 ℃ to 37 ℃ for 4 hours. Cell images were captured after being fixed (*Left*) and fluorescence intensities of Dma1-mNeonGreen at cell ends were quantified (*Right*). A.U., arbitrary units.

For graphs in **a**, **b**, and **c**, pooled data from three independent experiments are shown as mean ± SD; *n* indicates total cell numbers counted or measured for each strain; *p* values were determined by two-tailed unpaired *t*-test.

Scale bars, 10 μm.

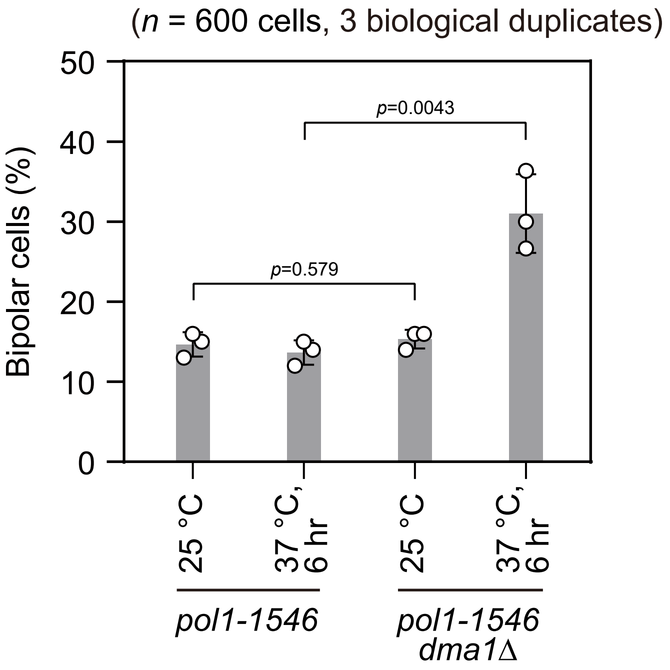

(n = 600 cells, 3 biological duplicates)
Bipolar cells (%)
50
40
30
20
10
0
p=0.0043
p=0.579
25 °C
37 °C, 6 hr
25 °C
37 °C, 6 hr
pol1-1546
pol1-1546 dma1Δ

Supplementary Figure 7. Related to Figure 4b.

Inhibition of bipolar growth is compromised in *dma1Δ* cells when defective DNA replication is induced by *pol1-1546* mutation. The yeast strains with indicated mutations were either cultured at 25 ℃ or shifted from 25 ℃ to 37 ℃ for 6 hours. Cells were collected, fixed and stained with calcofluor, and then polar growth pattern was scored. Pooled data from three independent experiments are shown in graph as mean ± SD; *n* indicates total cell numbers analyzed for each strain; *p* values were determined by two-tailed unpaired *t*-test.

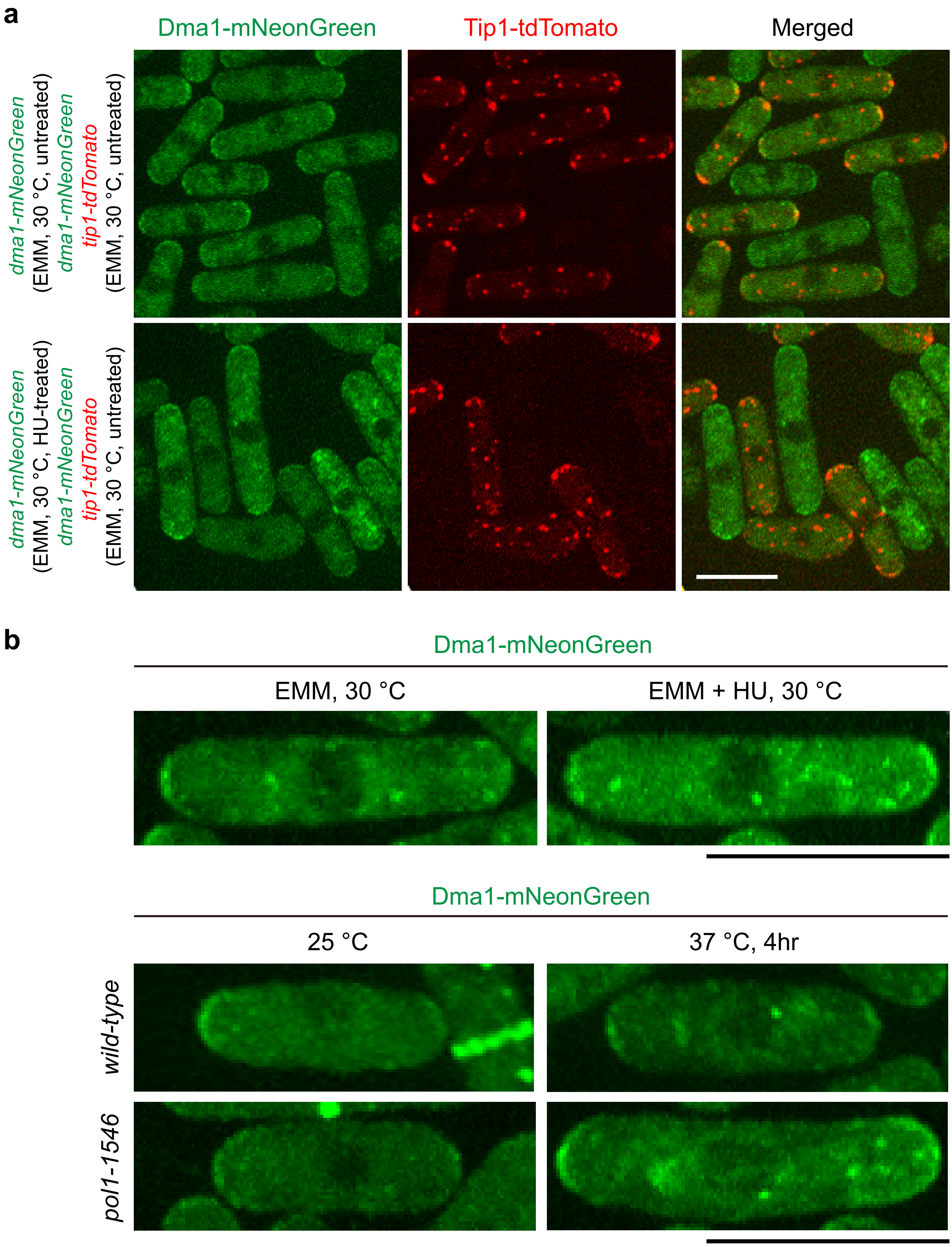
a
Dma1-mNeonGreen
Tip1-tdTomato
Merged
dma1-mNeonGreen
(EMM, 30 °C, untreated)
dma1-mNeonGreen
tip1-tdTomato
(EMM, 30 °C, untreated)
dma1-mNeonGreen
(EMM, 30 °C, HU-treated)
dma1-mNeonGreen
tip1-tdTomato
(EMM, 30 °C, untreated)
b
Dma1-mNeonGreen
EMM, 30 °C
EMM + HU, 30 °C
Dma1-mNeonGreen
25 °C
37 °C, 4hr
wild-type
pol1-1546

Supplementary Figure 8. Related to Figure 4c.

(**a**) To rule out the possible artifacts with fluorescence intensities caused by different imaging settings when Dma1-mNeonGreen was separately visualized before and after HU treatment, localization of Dma1-mNeonGreen at cell ends was simultaneously compared. Two yeast strains *dma1-mNeonGreen* and *dma1-mNeonGreen tip1-tdTomato* were treated similarly (both were not treated with HU) (*Left panel*) or differently (one treated with HU and the other not) (*Right panel*) after being grown in EMM at 30 ℃, then mixed at roughly 1:1 ratio before being prepared for imaging. It confirmed that HU treatment indeed promoted accumulation of Dma1 at cell ends.

(**b**) The representative cells outlined in Figure 4c are enlarged for better visualization of Dma1-mNeonGreen signals at cell ends.

Scale bars, 10 μm.

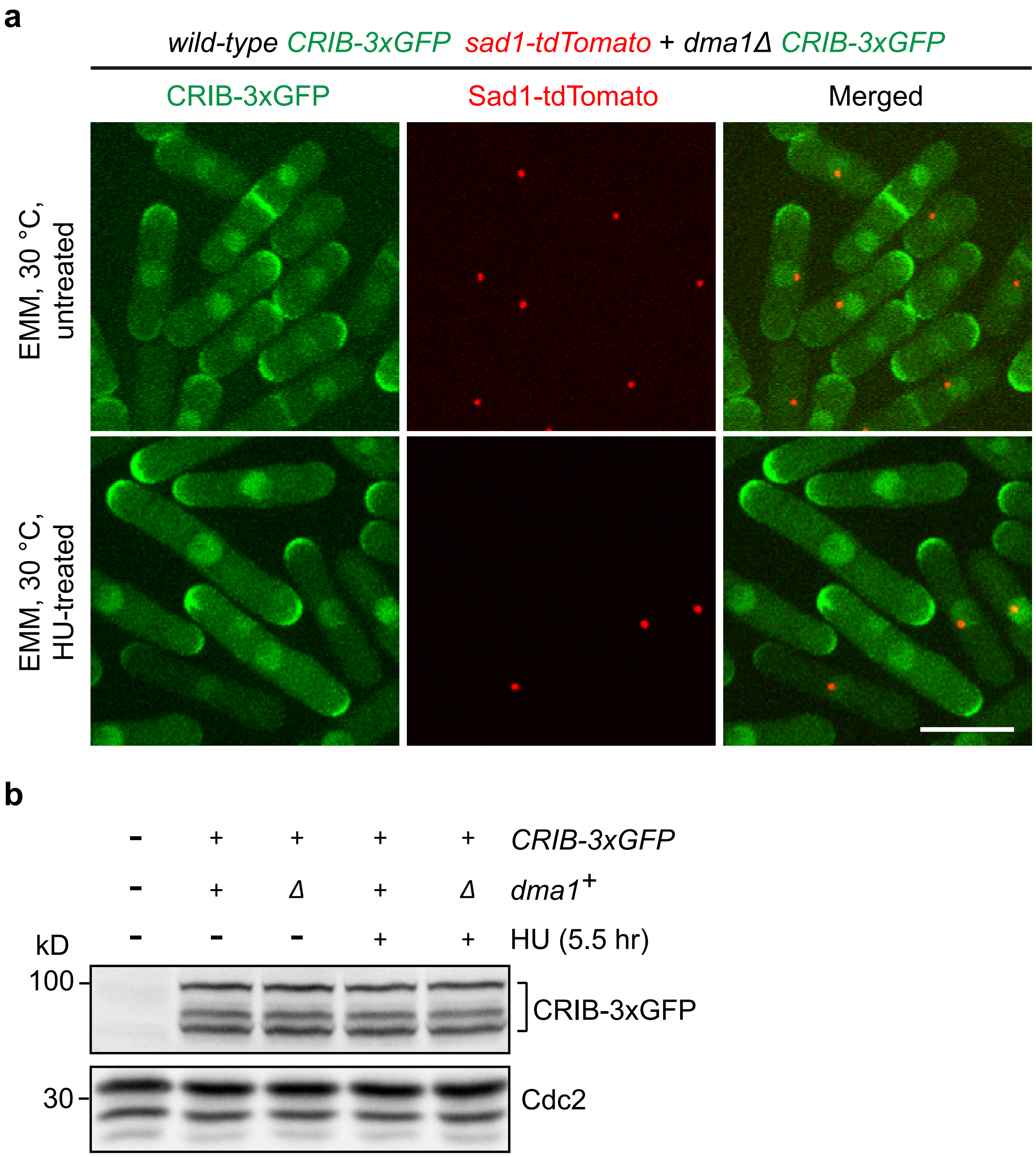
a
wild-type CRIB-3xGFP sad1-tdTomato + dma1Δ CRIB-3xGFP
CRIB-3xGFP
Sad1-tdTomato
Merged
EMM, 30 °C, untreated
EMM, 30 °C, HU-treated
b
CRIB-3xGFP
dma1+
HU (5.5 hr)
kD
100
30
CRIB-3xGFP
Cdc2

Supplementary Figure 9. Related to Figure 4d.

(**a**) To rule out the possible artifacts with fluorescence intensities caused by different imaging settings when CRIB-3xGFP was separately visualized before and after HU treatment in wild-type and *dma1Δ* cells, localization of CRIB-3xGFP at cell ends was simultaneously compared. Two yeast strains *CRIB-3xGFP sad1-tdTomato* and *dma1Δ CRIB-3xGFP* were treated similarly without (*Left panel*) or with (*Right panel*) HU after being grown in EMM at 30 ℃, then mixed at roughly 1:1 ratio before being prepared for imaging. *dma1Δ* cells were identified by cells not containing Sad1-tdTomato signals. It confirmed that more CRIB-3xGFP is accumulated at cell ends in *dma1Δ* cells compared to wild-type cells upon defective DNA replication induced by HU. Scale bar, 10 μm.

(**b**) Intracellular levels of CRIB-3xGFP are comparable in HU-treated *wild-type* and *dma1Δ* cells. Whole cell lysates were prepared from *wild-type* or *dma1Δ* cells grown in EMM5S and treated without or with 12 mM HU for 5.5 hr. Samples were subjected to Western blot analyses using anti-GFP and anti-Cdc2 antibodies.

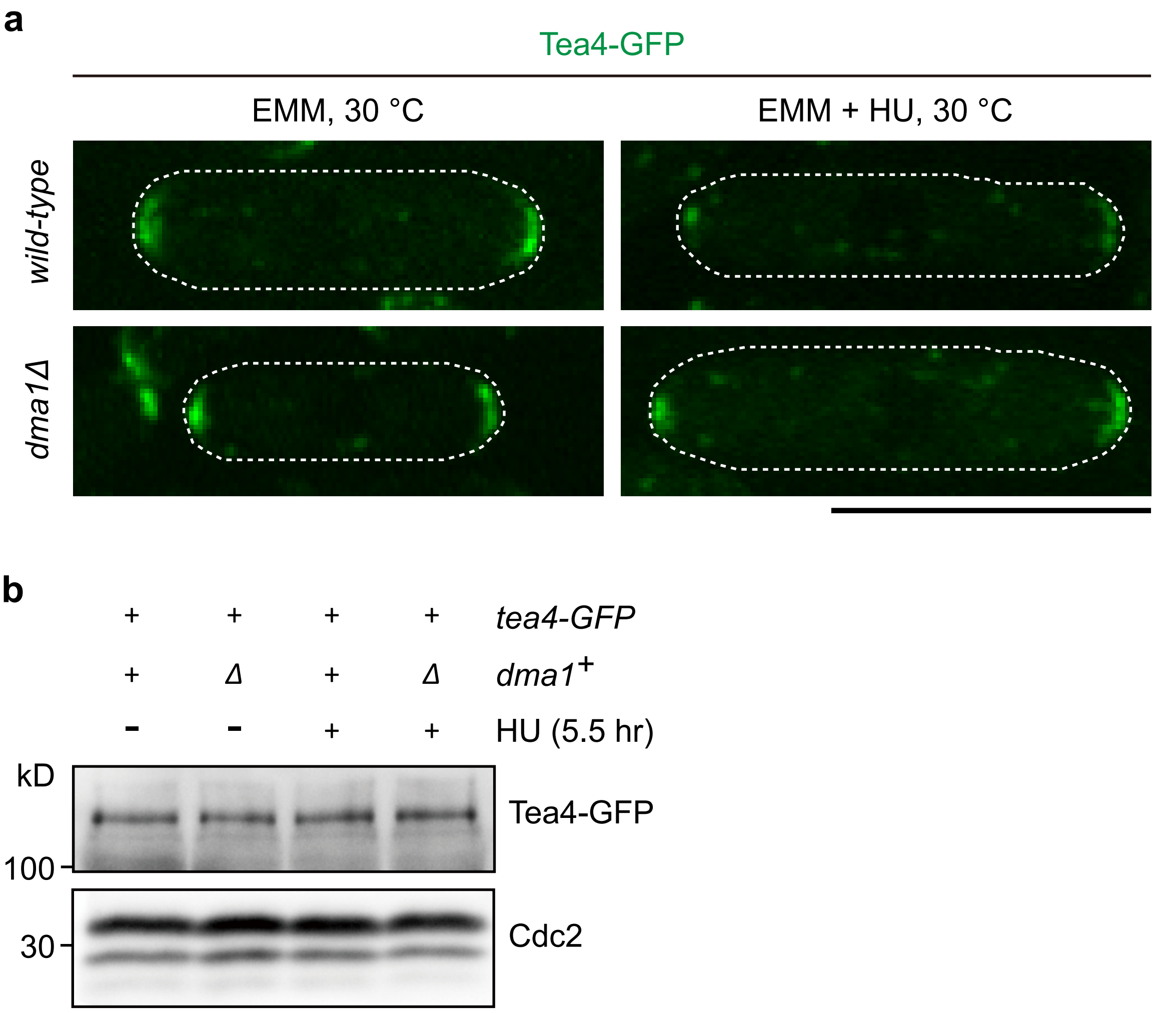

a
Tea4-GFP
EMM, 30 °C
EMM + HU, 30 °C
wild-type
dma1Δ
b
+ + + + tea4-GFP
+ Δ + Δ dma1+
- - + + HU (5.5 hr)
kD
100
30
Tea4-GFP
Cdc2

Supplementary Figure 10. Related to Figure 4e.

(**a**) The representative cells outlined in Figure 4e are enlarged for better visualization of Tea4-GFP signals at cell ends. Scale bar, 10 μm.

(**b**) Intracellular levels of Tea4-GFP are comparable in HU-treated *wild-type* and *dma1Δ* cells. Whole cell lysates were prepared from *wild-type* or *dma1Δ* cells grown in EMM5S and treated without or with 12 mM HU for 5.5 hr. Samples were subjected to Western blot analyses using anti-GFP and anti-Cdc2 antibodies.

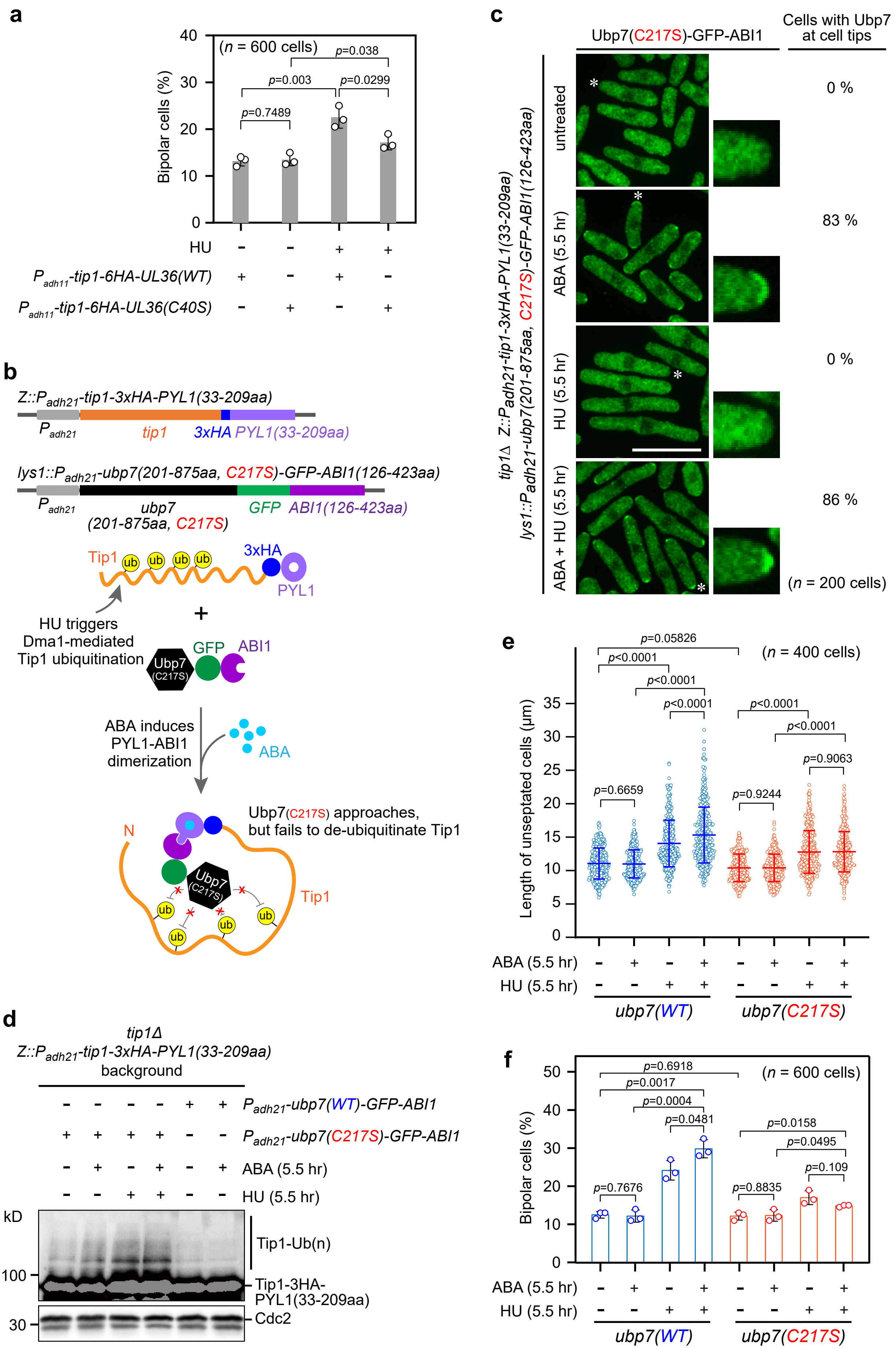

a
(n = 600 cells)
Bipolar cells (%)
p=0.7489
p=0.003
p=0.0299
p=0.038
HU
P adh11-tip1-6HA-UL36(WT)
P adh11-tip1-6HA-UL36(C40S)
b
Z::P adh21-tip1-3xHA-PYL1(33-209aa)
P adh21
tip1
3xHA
PYL1(33-209aa)
lys1::P adh21-ubp7(201-875aa, C217S)-GFP-ABI1(126-423aa)
ubp7
(201-875aa, C217S)
GFP
ABI1(126-423aa)
Tip1
ub
3xHA
PYL1
HU triggers
Dma1-mediated
Tip1 ubiquitination
Ubp7
(C217S)
GFP
ABI1
ABA induces
PYL1-ABI1
dimerization
ABA
N
Ubp7(C217S) approaches,
but fails to de-ubiquitinate Tip1
Tip1
c
Ubp7(C217S)-GFP-ABI1
Cells with Ubp7
at cell tips
tip1Δ Z::P adh21-tip1-3xHA-PYL1(33-209aa)
lys1::P adh21-ubp7(201-875aa, C217S)-GFP-ABI1(126-423aa)
untreated
0 %
ABA (5.5 hr)
83 %
HU (5.5 hr)
0 %
ABA + HU (5.5 hr)
86 %
(n = 200 cells)
d
tip1Δ
Z::P adh21-tip1-3xHA-PYL1(33-209aa)
background
P adh21-ubp7(WT)-GFP-ABI1
P adh21-ubp7(C217S)-GFP-ABI1
ABA (5.5 hr)
HU (5.5 hr)
kD
100
30
Tip1-Ub(n)
Tip1-3HA-
PYL1(33-209aa)
Cdc2
e
(n = 400 cells)
Length of unseptated cells (µm)
p=0.05826
p<0.0001
p<0.0001
p<0.0001
p=0.6659
p<0.0001
p<0.0001
p=0.9063
p=0.9244
ABA (5.5 hr)
HU (5.5 hr)
ubp7(WT)
ubp7(C217S)
f
(n = 600 cells)
Bipolar cells (%)
p=0.6918
p=0.0017
p=0.0004
p=0.0481
p=0.7676
p=0.0158
p=0.0495
p=0.109
p=0.8835
ABA (5.5 hr)
HU (5.5 hr)
ubp7(WT)
ubp7(C217S)

Supplementary Figure 11. Related to Figure 5c and Figure 5d-h.

(**a**) Fusing an active but not an inactive (C40S) deubiquitinating enzyme module of UL36 *in cis* with Tip1 promotes bipolar growth. The yeast strains with indicated genotypes were grown in EMM5S and treated without or with 12 mM HU for 5.5 hr. Cells were then collected, fixed and stained with calcofluor, and polar growth pattern was scored. Pooled data from three independent experiments are shown in graph as mean ± SD; *n* indicates total cell numbers analyzed for each strain; *p* values were determined by two-tailed unpaired *t*-test.

(**b-f**) Forced targeting of a catalytically dead version of deubiquitinating enzyme module of Ubp7 (i.e. Ubp7(C217S)) to cell ends fails to suppress Tip1 hyper-ubiquitination and is unable to promote cell bipolar growth. (**b**) Scheme of the design of the Tip1-PYL and Ubp7-ABI constructs, in which Ubp7(201-875aa)-GFP-ABI1(126-423aa) was replaced by Ubp7(201-875aa, C217S)-GFP-ABI1(126-423aa), which is presumably unable to de-ubiquitinate Tip1 even in its proximity. (**c**) Ubp7(201-875aa)-GFP-ABI1(126-423aa) fusion protein is sufficiently recruited to cell tips by Tip1-PYL in the presence of ABA. Both Tip1-3xHA-PYL1(33-209aa) and Ubp7(201-875aa)-GFP-ABI1(126-423aa) were ectopically expressed in *tip1Δ* strain. Cells were grown in EMM5S liquid medium at 30 ℃, then treated with 12 mM HU or 250 μM ABA separately or simultaneously for 5.5 hr. Images of fixed cells were captured and the percentages of cells with Ubp7-GFP-ABI signals at cell ends were scored. Asterisks indicate cell tips enlarged in insets. Scale bar, 10 μm. (**d-f**) Cells were grown and treated without or with HU or ABA as in (**c**) and then Tip1-3xHA-PYL1(33-209aa) was detected by immunoblotting (**d**), cell length of live unseptated cells was measured (**e**), and fixed cells were stained with calcofluor to quantify polar growth pattern (**f**). In graphs in **e** and **f**, pooled data from three independent experiments are shown as mean ± SD; *n* indicates total cell numbers measured or analyzed for each strain; *p* values were determined by two-tailed unpaired *t*-test.

Note that the yeast strains used in experiments shown in Supplementary Fig. **11c-f** were all with prototrophic genotypes (ade^+ leu^+ ura^+ his^+ lys^+), and the data for cell length measurements in a prototrophic strain (JY10459) (shown in this Supplementary Fig. **11e**) were almost identical to those obtained in the strain (JY10458) with auxotrophic genotypes (see Fig. **5h**).

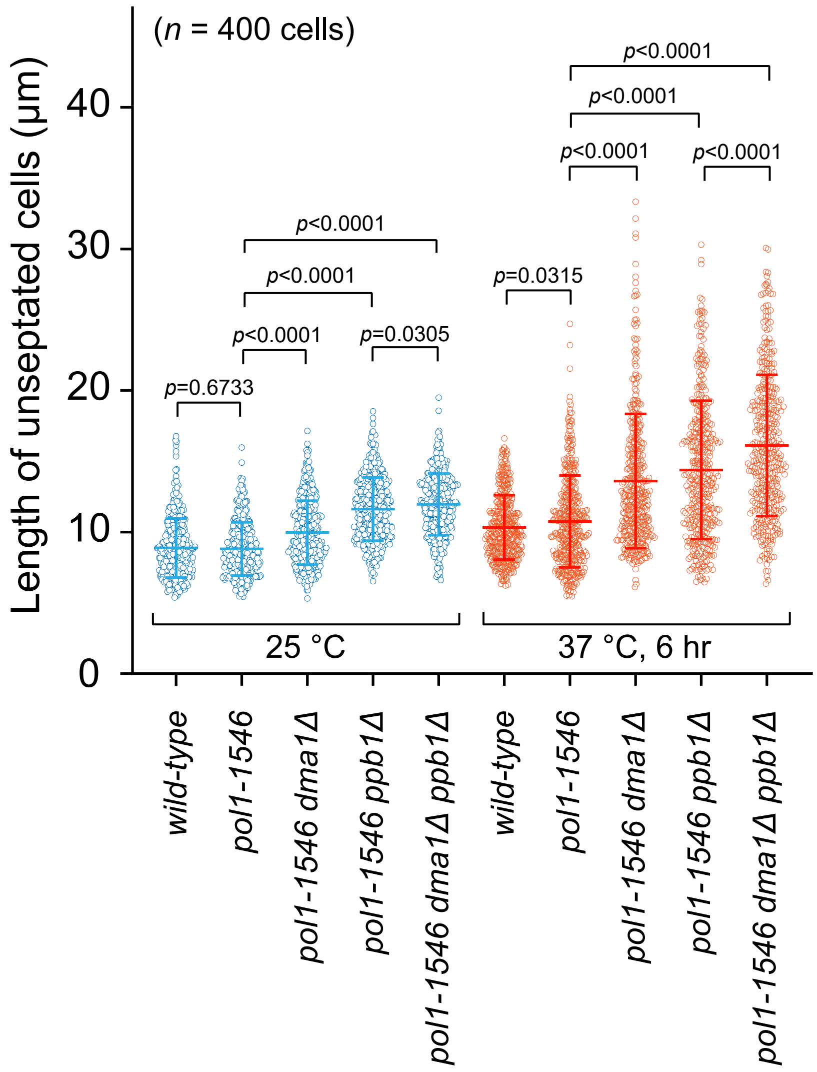
(n = 400 cells)
Length of unseptated cells (μm)
0
10
20
30
40
p=0.6733
p<0.0001
p<0.0001
p=0.0305
p<0.0001
p=0.0315
p<0.0001
p<0.0001
p<0.0001
p<0.0001
25 °C
37 °C, 6 hr
wild-type
pol1-1546
pol1-1546 dma1Δ
pol1-1546 ppb1Δ
pol1-1546 dma1Δ ppb1Δ
wild-type
pol1-1546
pol1-1546 dma1Δ
pol1-1546 ppb1Δ
pol1-1546 dma1Δ ppb1Δ

Supplementary Figure 12. Related to Figure 6b.

To exclude the possible impact of different auxotroph requirements on cell length, *wild-type* or *pol1-1546* yeast strains harboring *dma1Δ* or *ppb1Δ* mutations with all prototrophic genotypes were constructed or chosen for cell length measurements. Cells with indicated genotypes were either cultured at 25 ℃ or shifted from 25 ℃ to 37 ℃ for 6 hr. Live images were captured and cell length of unseptated cells was measured. Data are presented in graph as mean ± SD from three independent experiments; *n* indicates total cell numbers measured for each strain; *p* values were determined by two-tailed unpaired *t*-test.

Note that the data for cell length measurements in prototrophic strains (shown in this Figure) were almost identical to those obtained in strains with auxotrophic genotypes (see Fig. **6b**). The data in this Figure still supports the conclusion that *dma1Δ* and *ppb1Δ* have additive effect on cell length upon defective DNA replication induced by *pol1-1546* mutation. Yeast strains used in this Figure include: JY9489 (*wild-type*), JY4557 (*pol1-1546*), JY10149 (*pol1-1546 dma1Δ*), JY10200 (*pol1-1546 ppb1Δ*) and JY10198 (*pol1-1546 dma1Δ ppb1Δ*).

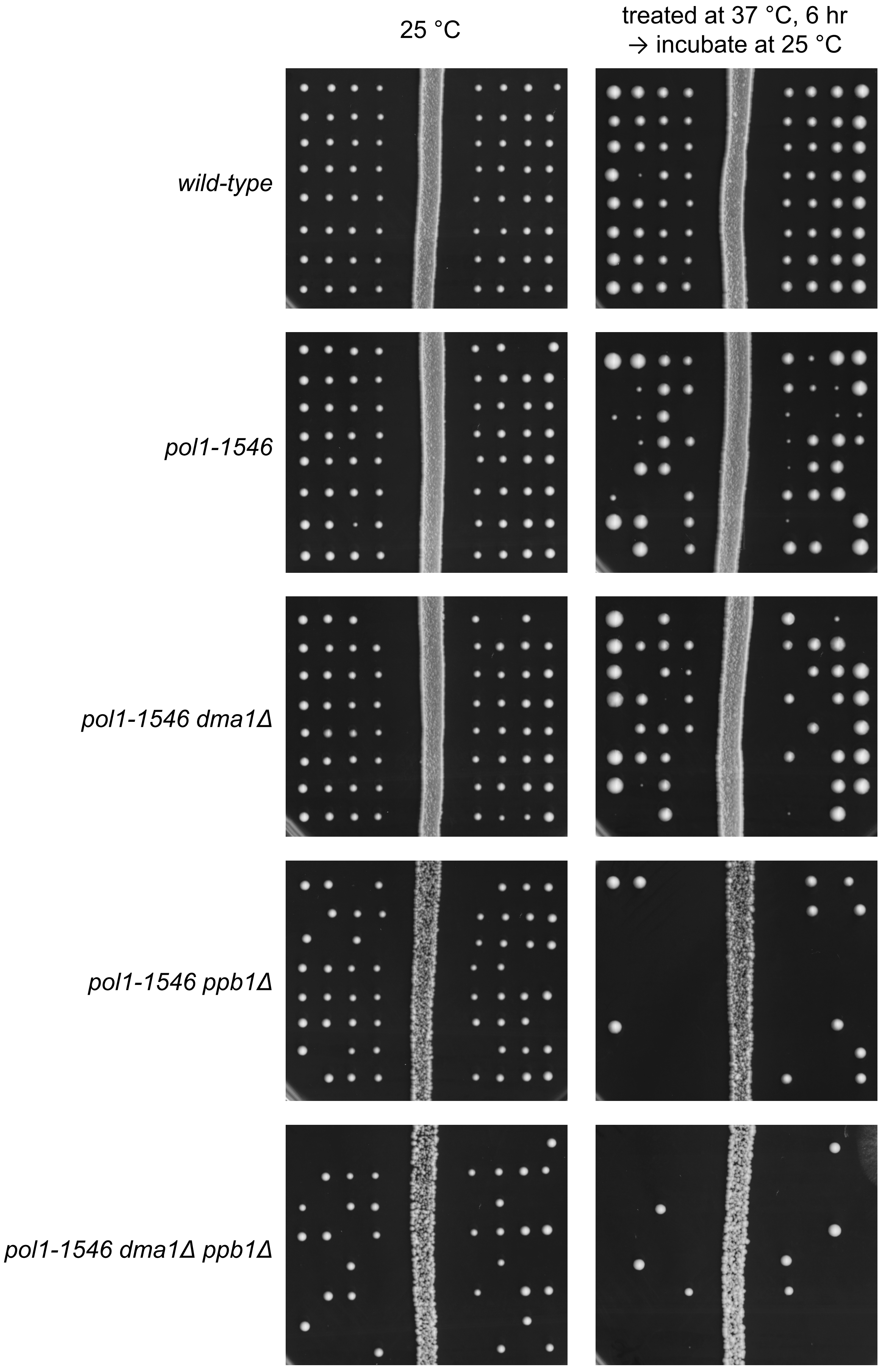
25 °C
treated at 37 °C, 6 hr
→ incubate at 25 °C
wild-type
pol1-1546
pol1-1546 dma1Δ
pol1-1546 ppb1Δ
pol1-1546 dma1Δ ppb1Δ

Supplementary Figure 13. Related to Figure 6d.

Examples of YES plates with colonies after growth at 25 ℃. Yeast strains with indicated genotypes were first cultured in liquid YES at either 25 ℃ or shifted from 25 ℃ to 37 ℃ for 6 hours. Normal-looking cells of each strain were then picked and placed on YES plates using a micromanipulator, the plates were incubated at 25 ℃ for > 3 days.

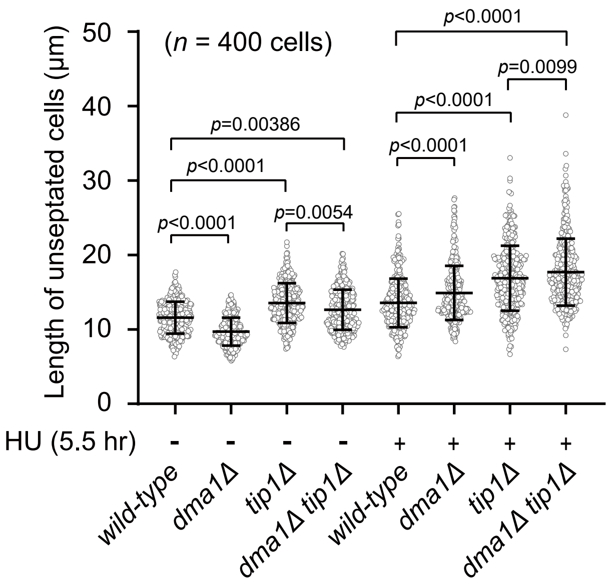

(n = 400 cells)
Length of unseptated cells (µm)
0
10
20
30
40
50
p<0.0001
p<0.0001
p=0.00386
p=0.0054
p<0.0001
p=0.0099
p<0.0001
p<0.0001
HU (5.5 hr)
- - - - + + + +
wild-type
dma1Δ
tip1Δ
dma1Δ tip1Δ
wild-type
dma1Δ
tip1Δ
dma1Δ tip1Δ

Supplementary Figure 14. Deletion of *tip1*$^{+}$ does not rescue the increased growth phenotype of HU-treated *dma1*Δ cells.

Cells of indicated genotypes grown in EMM5S liquid medium at 30 ℃ before or after being treated with 12 mM HU for 5.5 hr were collected. Live images were captured and cell length of unseptated cells was measured (data from each individual cells are represented by circles). Data are presented in graph as mean ± SD from three independent experiments; *n* indicates total cell numbers measured for each strain; *p* values were determined by two-tailed unpaired *t*-test.

Supplementary Table 1. Yeast strains used in this study.

Strain No.	Genotype	Source
JY1	*h*$^{-}$ *leu1-32 ura4-D18 ade6-210*	Lab stock
JY2	*h*$^{+}$ *leu1-32 ura4-D18 ade6-210*	Lab stock
JY69	*h*$^{-}$ *dma1-GFP::kanMX6 leu1-32 ura4-D18 ade6-21x*	(Guertin et al., 2002)
JY78	*h*$^{-}$ *dma1Δ::ura4*$^{+}$ *ura4-D18 leu1-32 ade6-21x*	(Guertin et al., 2002)
JY494	*h*$^{+}$ *tea1Δ::ura4*$^{+}$ *ura4-D18 leu*$^{-}$	Lab stock
JY496	*h*$^{-}$ *tea2Δ::his3*$^{+}$ *his3-D1 leu-32 ura4-D18 ade6-M21x*	Lab stock
JY564	*h*$^{-}$ *tea1-GFP::kanMX6 ade6 leu1-32 ura4-D18*	Lab stock
JY1387	*h*$^{+}$ *mCherry-atb2::hphMX6 leu1-32 ura4-D18 ade6-M21x*	Lab stock
JY1480	*h*$^{+}$*tip1-tdTomato::hphMX6 leu1-32 ura4-D18 ade6-M21x*	(Martin-Garcia and Mulvihill, 2009)
JY1482	*h*$^{+}$ *dma1Δ::kanMX6 tip1-tdTomato::hphMX6 leu1-32 ura4-D18 ade6-M21x*	This study
JY1737	*h*$^{+}$ *tip1-6HA::kanMX6 ura4-D18 leu1-32 ade6-210*	(Grallert et al., 2006)
JY1788	*h*$^{+}$ *dma1Δ::ura4*$^{+}$ *tip1-6HA::kanMX6 ura*$^{-}$ *leu*$^{-}$ *ade*$^{-}$	This study
JY1789	*h*$^{-}$ *dma1Δ::ura4*$^{+}$ *tip1-6HA::kanMX6 ura*$^{-}$ *leu*$^{-}$ *ade*$^{-}$	This study
JY1798	*h*$^{-}$ *GFP-atb2*$^{+}$*::kanMX6 tip1-tdTomato::hphMX6 ura4-D18 leu1-32*	This study
JY2742	*h*$^{+}$ *mts3-1 dma1Δ::ura4*$^{+}$ *tip1-6HA::kanMX6 ura*$^{-}$ *leu*$^{-}$	This study
JY2744	*h*$^{+}$ *mts3-1 tip1-6HA::kanMX6 ura*$^{-}$ *leu*$^{-}$	This study
JY2818	*h*$^{-}$ *dma1Δ::ura4*$^{+}$ *tip1-tdTomato::hphMX6 GFP-atb2*$^{+}$*-kanMX6 ura4-D18 leu1-32*	This study
JY3232	*h*$^{+}$ *mts3-1 dma1-I194A-GFP::kanMX6 tip1-6HA::kanMX6 ura*$^{-}$ *leu*$^{-}$	This study
JY3261	*h*$^{-}$ *cdc25-22 tip1-6HA::kanMX6 ura*$^{-}$ *leu*$^{-}$ *ade*$^{-}$	This study
JY3477	*h*$^{+}$ *mts3-1 dma1-R64A-GFP::kanMX6 tip1-6HA::kanMX6 ura*$^{-}$ *leu*$^{-}$	This study
JY3479	*h*$^{+}$ *dma1-R64A-GFP::kanMX6 tip1-6HA::kanMX6 ura*$^{-}$ *leu*$^{-}$	This study
JY3534	*h*$^{?}$ *dma1-I194A-GFP::kanMX6 tip1-6HA::kanMX6 ura*$^{-}$ *leu*$^{-}$	This study
JY3551	*h*$^{-}$ *lys1Δ::P*$_{adh21}$*-CFP-mod5::hphMX6::lys1* dma1Δ::ura4*$^{+}$ *leu1-32 ade6-210*	This study
JY3560	*h*$^{-}$ *mts3-1 tea1Δ::ura4*$^{+}$ *tip1-6HA::kanMX6 ura*$^{-}$ *leu1*$^{-}$	This study
JY3762	*h? nda3-KM311 tip1-6HA::kanMX6 ura*$^{-}$ *leu*$^{-}$ *ade*$^{-}$	This study
JY3826	*h*$^{-}$ *tip1-tdTomato::hphMX6 tea2Δ::his3*$^{+}$ *his3-D1 leu-32 ura4-D18 ade6-M21x*	This study

JY3827	$h^{?}$ tip1-6HA::kanMX6 mal3Δ::kanMX6 ade6-M210 ura^{-} leu^{-}	This study
JY3828	h^{-} tea2Δ::$his3^{+}$ tip1-6HA::kanMX6 his3-D1 leu^{-} $ura4^{-}$ ade^{-}	This study
JY3835	$h^{?}$ mts3-1 mal3Δ::kanMX6 tip1-6HA::kanMX6 ade6-M210 ura^{-} leu^{-}	This study
JY3860	$h^{?}$ mal3Δ::kanMX6 GFP-$atb2^{+}$::kanMX6 tip1-tdTomato::hphMX6 leu1-32 ade6-M210	This study
JY3884	$h^{?}$ mts3-1 tea2Δ::$his3^{+}$ tip1-6HA::kanMX6 leu^{-} $ura4^{-}$	This study
JY3899	h^{-} tip1-tdTomato::hphMX6 GFP-$atb2^{+}$::kanMX6 tea2Δ::$his3^{+}$ his3-D1 leu-32 ura4-D18 ade6-M21x	This study
JY4192	$h^{?}$ lys1Δ::P_{adh21}-dma1-CFP::hphMX6::lys1* dma1Δ::$ura4^{+}$ tip1-6HA::kanMX6 leu1-32 ura4-D18 ade6-216	This study
JY4193	$h^{?}$ lys1Δ::P_{adh21}-dma1(R64A)-CFP-mod5::hphMX6::lys1* dma1Δ::$ura4^{+}$ tip1-6HA::kanMX6 $leu1^{-}$ $ura4^{-}$ $ade6^{-}$	This study
JY4278	$h^{?}$ lys1Δ::P_{adh21}-dma1-CFP-mod5::hphMX6::lys1* dma1Δ::$ura4^{+}$ tip1-6HA::kanMX6 ura^{-} leu^{-} ade^{-}	This study
JY4209	h^{+} pol1-1546 tip1Δ::kan^{R} leu1-32::pKK28 integrated (P_{tip1}-$tip1^{+}$-GFP-nat^{R}, $leu1^{+}$) his2	(Kume et al., 2011)
JY4321	$h^{?}$ pol1-1546 tip1-6HA::KanMX6 leu-	This study
JY4322	h^{+} pol1-1546 dma1Δ::$ura4^{+}$ tip1-6HA::KanMX6	This study
JY4466	h^{-} lys1Δ::P_{adh21}-dma1(R64A)-CFP::hphMX6::lys1* dma1Δ::$ura4^{+}$ leu1-32 ade6-210	This study
JY4468	h^{-} lys1Δ::P_{adh21}-dma1(R64A)-CFP-mod5::hphMX6::lys1* dma1Δ:$ura4^{+}$ ade6-210 leu1-32	This study
JY4470	h^{-} lys1Δ::P_{adh21}-dma1(I194A)-CFP-mod5::hphMX6::lys1* dma1Δ:$ura4^{+}$ leu1-32 ade6-210	This study
JY4472	h^{+} lys1Δ::P_{adh21}-dma1-CFP::hphMX6::lys1* dma1Δ:$ura4^{+}$ leu1-32 ade6-210	This study
JY4474	h^{-} lys1Δ::P_{adh21}-dma1(R64A;I194A)-CFP-mod5::hphMX6::lys1* dma1Δ::$ura4^{+}$ leu1-32 ade6-210	This study
JY4476	h^{-} lys1Δ::P_{adh21}-dma1-CFP-mod5::hphMX6::lys1* dma1Δ:$ura4^{+}$ leu1-32 ade6-210	This study
JY4504	h^{+} lys1Δ::P_{adh21}-dma1-R64A-CFP::hphMX6::lys1* dma1Δ::$ura4^{+}$ tip1-6HA::kanMX6 leu1-32 ura4-D18 ade6-216	This study
JY4506	h^{-} lys1Δ::P_{adh21}-dma1-I194A-CFP-mod5::hphMX6::lys1* dma1Δ::$ura4^{+}$ tip1-6HA::kanMX6 $leu1^{-}$ $ura4^{-}$ $ade6^{-}$	This study
JY4507	h^{-} 1ysΔ::P_{adh21}-dma1-(R64A,I194A)-CFP-mod5::hphMX6::lys1* dma1Δ::$ura4^{+}$ tip1-6HA::kanMX6 $leu1^{-}$ $ura4^{-}$ $ade6^{-}$	This study
JY4961	h^{+} dma1Δ::$ura4^{+}$ tip1-6HA::kanMX6 leu1-32::P_{nmt1}-6His-myc-Ubiquitin::leu^{+} ura^{-} leu1-32 ade^{-}	This study
JY4972	h^{+} tip1-6HA::kanMX6 leu1-32::P_{nmt1}-6His-myc-Ubiquitin::leu^{+} ura^{-} leu1-32 ade^{-}	This study

JY5082	h^+ tip1-6HA::kanMX6 leu1-32::P_{nmt1}-6His-myc-Ubiquitin(K0)::leu^+ ura^- leu^-ade^-	This study
JY5083	h^+ dma1Δ::$ura4^+$ tip1-6HA::kanMX6 leu1-32::P_{nmt1}-6His-myc-Ubiquitin(K0)::leu^+ ura^- leu^-ade^-	This study
JY5084	h^+ tip1-6HA::kanMX6 leu1-32::P_{nmt1}-6His-myc-Ubiquitin(K48)::leu^+ ura^- leu^- ade^-	This study
JY5085	h^+ dma1Δ::$ura4^+$ tip1-6HA::kanMX6 leu1-32::P_{nmt1}-6His-myc-Ubiquitin(K48)::leu^+ ura^- leu^- ade^-	This study
JY5086	h^+ tip1-6HA::kanMX6 leu1-32::P_{nmt1}-6His-myc-Ubiquitin(K63)::leu^+ ura^- leu^- ade^-	This study
JY5087	h^+ dma1Δ::$ura4^+$ tip1-6HA::kanMX6 leu1-32::P_{nmt1}-6His-myc-Ubiquitin(K63)::leu^+ ura^- leu^- ade^-	This study
JY5267	h^+ ura4-294::[shk1 promotor:ScGIC2 CRIB:GFP3:kan^R] leu1-32	(Tatebe et al., 2008)
JY5335	$h^?$ tip1-6HA::hphMX6 dma1-GFP::kanMX6 leu1-32 ura4-D18 ade6-21x	This study
JY5800	h^- dma1-GFP::kanMX6 tip1-tdTomato::hphMX6 ura4-D18 leu1-32 ade6-210	Lab stock
JY5804	h^+ lys1Δ::P_{adh21}-dma1(I194A)-CFP::hphMX6::lys1* dma1Δ::$ura4^+$ ura4-D18 leu1-32 ade6-21x	This study
JY5805	h^- lys1Δ::P_{adh21}-dma1(I194A)-CFP::hphMX6::lys1* dma1Δ::$ura4^+$ ura4-D18 leu1-32 ade6-21x	This study
JY6233	$h^?$ tip1-6HA::hygMX6 tea1-GFP:: kanMX6 ade6 leu1-32 ura4-D18	This study
JY6678	h^+ lys1Δ::P_{adh11}- dma1-GFP::hphMX6::lys1* tip1-6HA::kanMX6 ura4-D18 leu1-32 ade6-210	This study
JY6682	h^+ lys1Δ::P_{adh11}-$dma1^{N(1\text{-}173aa)FHA}$-GFP::hphMX6::lys1* tip1-6HA::kanMX6 ura4-D18 leu1-32 ade6-210	This study
JY6684	h^+ lys1Δ::P_{adh11}-$dma1^{C(167\text{-}267aa)RF}$-GFP::hphMX6::lys1* tip1-6HA::kanMX6 ura4-D18 leu1-32 ade6-210	This study
JY6689	h^+ lys1Δ::P_{adh11}-$dma1^{R64A}$-GFP::hphMX6::lys1* tip1-6HA::kanMX6 ura4-D18 leu1-32 ade6-210	This study
JY7057	h^- lys1Δ::P_{adh11}-$dma1^{N(1\text{-}173aa)FHA}$-GFP::hygMX6 tip1-6HA::KanMX6 ura4-D18 leu1-32 ade6-210	This study
JY7058	h^+ lys1Δ::P_{adh11}-$dma1^{N(1\text{-}173aa)FHA}$-GFP::hygMX6 ura4-D18 leu1-32 ade6-210	This study
JY7370	$h^?$ mts3-1 lys1Δ::P_{adh11}-$dma1^{N(1\text{-}173aa)FHA}$-GFP::hygMX6 tip1-6HA::KanMX6 ura4-D18 leu1-32 ade6-210 ?	This study
JY7516	h^- Z::P_{adh11}-$tip1^{(245\text{-}314aa)}$-6HA::kanMX6 lys1::$P_{adh11}$-$dma1^{N(1\text{-}173aa)FHA}$-GFP::hphMX6::lys1* tip1Δ::$ura4^+$ leu1-32 ura4-D18 ade6-M21x	This study
JY7523	h^+ Z::P_{adh11}-$tip1^{(135\text{-}244aa)}$-6HA::kanMX6 lys1::$P_{adh11}$-$dma1^{N(1\text{-}173aa)FHA}$-GFP::hphMX6::lys1* tip1Δ::$ura4^+$ leu1-32 ura4-D18 ade6-M21x	This study
JY7524	h^+ Z::P_{adh11}-$tip1^{(135\text{-}461aa)}$-6HA::kanMX6 lys1::$P_{adh11}$-$dma1^{N(1\text{-}173aa)FHA}$-GFP::hphMX6::lys1* tip1Δ::$ura4^+$leu1-32 ura4-D18 ade6-M21x	This study

JY7526	h^{+} Z::P$_{adh11}$-tip1$^{(315-461aa)}$-6HA::kanMX6 lys1::P$_{adh11}$-dma1$^{N(1-173aa)FHA}$-GFP::hphMX6::lys1* tip1Δ::ura4^{+}leu1-32 ura4-D18 ade6-M21x	This study
JY7534	h$^{?}$ Z:: P$_{adh11}$-tip1$^{(full\ length)}$-6HA::kanMX6 lys1::P$_{adh11}$- dma1$^{N(1-173aa)FHA}$-GFP::hphMX6::lys1* tip1Δ::ura4^{+} leu1-32 ura4-D18 ade6-M21x	This study
JY7535	h$^{?}$ Z::P$_{adh11}$- tip1$^{(1-134aa)}$-6HA::kanMX6 lys1::P$_{adh11}$-dma1$^{N(1-173aa)FHA}$-GFP::hphMX6::lys1* tip1Δ::ura4^{+} leu1-32 ura4-D18 ade6-M21x	This study
JY7536	h^{+} Z::P$_{adh11}$-tip1$^{(1-244aa)}$-6HA::kanMX6 lys1::P$_{adh11}$-dma1$^{N(1-173aa)FHA}$-GFP::hphMX6::lys1* tip1Δ::ura4^{+} leu1-32 ura4-D18 ade6-M21x	This study
JY7780	h$^{?}$ pol1-1546 dma1-mNeonGreen::kanMX6 ade6 leu1-32 ura4-D18	This study
JY8169	h^{-} dma1-mNeonGreen::kanMX6 leu1-32 ura4-D18 ade6-210	This study
JY8202	h$^{?}$ dma1-mNeonGreen::kanMX6 ags1Δ3'UTR ags1::ags1-RFP::leu1^{+}:ura4^{+}	This study
JY8233	h$^{?}$ mal3Δ::kan^{R} dma1-mNeonGreen::kanMX6 mCherry-atb2::hph^{R} ade6- leu1-32 ura4-D18	This study
JY8251	h$^{?}$ tea1D::ura4^{+} dma1-mNeonGreen::kanMX6 tip1-tdTomato::hphMX6 ura4-D18 leu^{-}	This study
JY8252	h$^{?}$ dma1-mNeonGreen::kanMX6 tip1-tdTomato::hphMX6 ura4-D18 leu^{-}	This study
JY8263	h$^{?}$ cdc25-22 dma1-mNeonGreen::kanMX6 leu1-32 ura4-D18 ade6-210	This study
JY9819	h$^{?}$ dma1Δ::ura4^{+} ura4-294::[shk1 promotor:ScGIC2 CRIB:GFP3:kanMX6]	This study
JY9871	h$^{?}$ tea4-GFP::kanMX6 tip1-6HA::KanMX6 ade6 leu1-32 ura4-D18	This study
JY9872	h$^{?}$ dma1Δ::ura4^{+} tea4-GFP::kanMX6 tip1-6HA::KanMX6 ade6 leu1-32 ura4-D18	This study
JY10082	h$^{?}$ sad1-tdTomato::nat^{R} ura4-294::[shk1 promotor:ScGIC2 CRIB:GFP3:kanMX6]	This study
JY4556	h^{-} pol1-1546 ura4-D18 leu1-32 ade-?	Lab stock
JY5445	h$^{?}$ ppb1Δ::ura4+ tip1-6HA::Kan^{R} ura4-D18 leu1-32	This study
JY5446	h$^{?}$ ppb1Δ::ura4+ tip1-6HA::Kan^{R} dma1Δ::ura4+ ura4-D18 leu1-32	This study
JY10150	h^{-} pol1-1546 dma1Δ::kan^{R} ura4-D18 leu1-32	This study
JY10197	h$^{?}$ pol1-1546 ppb1Δ::ura4 ura4-D18 leu1-32	This study
JY10199	h$^{?}$ pol1-1546 dma1Δ::kanR ppb1Δ::ura4 ura4-D18 leu1-32	This study
JY3536	h- lys1Δ::P$_{adh21}$-CFP-mod5::hygMX6 dma1::ura4+ tip1-6HA::kan^{R} leu1^{-} ura4^{-} ade6^{-}	This study
JY10329	h^{-} lys1Δ::P$_{adh21}$-dma1(I194A)-CFP-mod5::hphMX6::lys1* dma1Δ::ura4^{+} tip1-6HA::kanMX6 leu1^{-} ura4^{-} ade6^{-}	This study

JY10406	h$^{?}$ tip1Δ::kan^{R} Z::P$_{adh11}$-tip1$^{(full\ length)}$-6HA-linker-UL36$^{15\text{-}260aa}$::kan^{R} leu1^{-} ura4^{-} ade6^{-}	This study
JY10407	h$^{?}$ tip1Δ::kan^{R} Z::P$_{adh11}$-tip1$^{(full\ length)}$-6HA-linker-UL36$^{15\text{-}260aa\ (C40S)}$::kan^{R} leu1^{-} ura4^{-} ade6^{-}	This study
JY10458	h^{+} tip1Δ::kan^{R} Z::P$_{adh21}$-tip1-3xHA-PYL1(33-209aa)::kan^{R} lys1Δ::P$_{adh21}$-ubp7(201-875aa)-GFP-ABI1(126-423a)::hyg^{R} ura4-D18 leu1-32 ade6-M210 his3-D1	This study
JY10459	h^{+} tip1Δ::kan^{R} Z::P$_{adh21}$-tip1-3xHA-PYL1(33-209aa)::kan^{R} lys1Δ::P$_{adh21}$-ubp7(201-875aa)-GFP-ABI1(126-423a)::hyg^{R} ade^{+} leu^{+} ura^{+} his^{+} lys^{+}	This study
JY10580	h$^{?}$ tip1Δ::kan^{R} Z::P$_{adh21}$-tip1-3xHA-PYL1(33-209aa)::kan^{R} lys1Δ::P$_{adh21}$-ubp7(201-875aa,C217S)-GFP-ABI1(126-423aa)::hyg^{R} ade^{+} leu^{+} ura^{+} his^{+} lys^{+}	This study
JY221	h^{-} tip1Δ::kan^{R} ura4-D18 leu1-32 ade6-210	Lab stock
JY7607	h^{+} dma1Δ::ura4^{+} tip1Δ::kanR ura4-D18 leu1-32 ade6-21x	This study
JY9489	h^{+} ade^{+} leu^{+} ura^{+} his^{+} lys^{+}	Lab stock
JY4557	h^{+}pol1-1546 ura4^{+} leu1^{+} ade6^{+}	This study
JY10149	h^{-} pol1-1546 dma1Δ::ura4^{+} ura4^{+} leu1^{+} ade6^{+}	This study
JY10200	h$^{?}$ pol1-1546 ppb1Δ::ura4^{+} ura4^{+} leu1^{+} ade6^{+}	This study
JY10198	h$^{?}$ pol1-1546 dma1Δ::ura4^{+} ppb1Δ::ura4^{+} ura4^{+} leu1^{+} ade6^{+}	This study
JY10633	h^{+} dma1Δ::ura4^{+} leu1^{+}::P$_{nmt41}$-NTAP-dma1^{+} tip1-6HA::kan^{R}	This study
JY10634	h^{+} dma1Δ::ura4^{+} leu1^{+}::P$_{nmt41}$-NTAP-dma1-5xGly-Ub(7K-->7R) tip1-6HA::kan^{R}	This study

Supplementary Table 2. A list of DNA oligonucleotides/primers.

Name	Sequence (5'→ 3')
dma1-5'-NdeI-F	GGAATTCCATATGACAAAATCTGTTGAG
dma1-ΔRF-BamH1-R	CGCATGGATCCTTATTCCGGAGGACCCGA
dma1-5'-SalI-F	ACGCGTCGACAAATGACAAAATCTG
dma1-3'-NotI-R	GTTTAGCGGCCGCCTCAGATGCATC
dma1-3'-BamHI-R	ATGCGGATCCTCCTCAGATGCATCGTC
dma1-5'-Sall-F	GCGCGTCGACTATGACAAAATCTGTTGAGGG
dma1-5'-BamHI-F	CGCGGATCCGCGATGACAAAATCTGAGGG
dma1-3'-Pst1-R	GAACTGCAGTTACTCAGATGCATCGTCTTTTAC
dma1-5'-SalI-F	GCAGCGTCGACT ATGACAAAATCTGTTGAGGG
dma1-3'-BamHI-R	CGCGGATCCCTCAGATGCATCGTCTTTTAC
dma1(173aa)-3'-BamHI-R	CGCGGATCCGAATTCGTTAAGGTTGTAAGG
dma1(167aa)-5'-SalI-F	GCAGCGTCGACTCCTTACAACCTTAACGAATTC
dma1-R267aa-BamHI	CGCATGGATCCTTACTCAGATGCATCGTC
dma1-R64A-F	ATTTACATAGGAGCATACACAGAGCGATACAACGG
dma1-R64A-R	GCTCTGTGTATGCTCCTATGTAAATGGGTAGATTAT
tea4-5'-EcoRI-F	CGCGAATTCATGTTACACATGAATAGTGC
tea4-3'-SalI-R	CGCGTCGACTCAGCGATTCGTATTTAAG
dma1-TAA up 257bp-F(w)	GCGGATCTTCTGAATCGGGTCC
dma1-TAA up 23bp-R(pFA6a-1'x)	CCGTCGACCTGCAGCGTACGACTCAGATGCATCGTCTTTT ACCG
dma1-TAA down 40bp-F(pFA6a-2'y)	GTTTAAACGAGCTCGAATTCATCGATCCTCAAGTTCTTCC TTTAATGACC
dma1-TAA down 331bp-R(z)	GATCAGGTGTGAAGGACGACAG
mod5-5'-KpnI-F	GGGGTACCATGTCGGCTTTATCTGAAAG
mod5-3'-SmaI-R	GCCCCCGGGTTACATCAAAATACAACAAAAC
dma1-R64A-F	ATTTACATAGGAGCATACACAGAGCGATACAACGG
dma1-R64A-R	GCTCTGTGTATGCTCCTATGTAAATGGGTAGATTAT
dma1-I194A mut-R	GGAATGTTGTGCTTGTCTTATGCCTGTTTTACCT
dma1-I194A mut-F	CAAGCACAACATTCCGGAGGACCCGATTCAGAAG
tip1-ATG-no tail-F	ATGTTTCCTCTTGGCAGTGTCG
tip1-Stop w/o TAA-no tail-R	AGCTTCGTCTGTGCTGCCAAATAC

tip1-ATG-tail-F	AGGAATTCCGTGACCATGTTTCCTCTTGGCAGTGTCG
tip1-Stop w/o TAA-tail-R	GAGCTCCAGTCGTGCAGCTTCGTCTGTGCTGCCAAATAC
tip1-402bp downstream of ATG -no tail-R	GGTTGGCGTTAAGGCAGTAG
tip1-402bp downstream of ATG-tail-R	GAGCTCCAGTCGTGCGGTTGGCGTTAAGGCAGTAG
tip1-732bp downstream of ATG-no tail-R	TTTTCCAGACATATACATCTCAAGTTG
tip1-732bp downstream of ATG-tail-R	GAGCTCCAGTCGTGCTTTTCCAGACATATACATCTCAAGTTG
tip1-403bp downstream of ATG -no tail-F	GAAAAAATTCTTCAGAAACGTATTG
tip1-403bp downstream of ATG-tail-F	AGGAATTCCGTGACCGAAAAAATTCTTCAGAAACGTATTG
tip1-733bp downstream of ATG-no tail-F	TCCGAAGATGATTTACTTTTTAGCC
tip1-942bp downstream of ATG-no tail-R	ATCGTTAGAACCTTTACCACG
tip1-733bp downstream of ATG-tail-F	AGGAATTCCGTGACCTCCGAAGATGATTTACTTTTTAGCC
tip1-942bp downstream of ATG-tail-R	GAGCTCCAGTCGTGCATCGTTAGAACCTTTACCACG
tip1-943bp downstream of ATG-no tail-F	TTACCTGAAAACCATCCTCAAC
tip1-943bp downstream of ATG-tail-F	AGGAATTCCGTGACCTTACCTGAAAACCATCCTCAAC
UL36-C40S-F	GTATCGTCCATGCGCTCGTCGCTGTCCTTTC
UL36-C40S-R	GCGCATGGACGATACCGACCCCCCGGCTC
Ubp7-C217S mut-F	GCCACATCGTTTTTTAATTCGACTTTACAAGTC
Ubp7-C217S mut-R	AAAAAACGATGTGGCACCCAAATTTTTTAAACC
Ubiquitin-K33R mut-F	CGTTAGAGCTAGAATTCAAGACAGAGAAGGC
Ubiquitin-K33R mut-R	GTTGATCAGGTGGAATGCCTTCTCTGTCTTG
Ubiquitin-R48K mut-F	CAAAGATTGATCTTTGCCGGTAAGCAGCTCG
Ubiquitin-R48K mut-R	GTTCTACCGTCCTCGAGCTGCTTACCGGCA
Ubiquitin-R63K mut-F	CTGTCTGATTACAACATTCAGAAGGAGTCG
Ubiquitin-R63K mut-R	GACAAGATGTAAGGTCGACTCCTTCTGAATG

Supplementary Table 3. A list of primary and secondary antibodies used in the study.

Antibody	Supplier	Catalog No.	Application	Usage
Peroxidase-anti-peroxidase (PAP) soluble complex	Sigma-Aldrich	P1291	WB	1:800
Rabbit polyclonal anti-Myc	GeneScript	A00172-40	WB	1:1000
Mouse monoclonal anti-GFP (clone 7.1/13.1)	Roche	11814460001	IP	1 μg
Mouse monoclonal anti-GFP (clone ME11)	Beijing Ray Antibody Biotech	RM1008	WB	1:1000
Rat monoclonal anti-HA (clone 3F10)	Roche	11867423001	WB/IP	1:1000/ 1 μg
Mouse monoclonal anti-Cig2 (clone 3A11/5)	Santa Cruz Biotechnology	sc-53223	WB	1:1000
Rabbit polyclonal anti-PSTAIRE (Cdc2)	Santa Cruz Biotechnology	sc-53	WB	1:1000
Goat anti-mouse polyclonal IgG (H+L) HRP conjugate	Thermo Fisher Scientific	#31430	WB	1:5000-10000
Goat anti-rabbit polyclonal IgG (H+L) HRP conjugate	Thermo Fisher Scientific	#32460	WB	1:5000-10000

Supplementary References:

1. Guertin, D.A., Venkatram, S., Gould, K.L. & McCollum, D. Dma1 prevents mitotic exit and cytokinesis by inhibiting the septation initiation network (SIN). *Dev Cell* **3**, 779-790 (2002).
2. Martin-Garcia, R. & Mulvihill, D.P. Myosin V spatially regulates microtubule dynamics and promotes the ubiquitin-dependent degradation of the fission yeast CLIP-170 homologue, Tip1. *Journal of Cell Science* **122**, 3862-3872 (2009).
3. Grallert, A. et al. S. pombe CLASP needs dynein, not EB1 or CLIP170, to induce microtubule instability and slows polymerization rates at cell tips in a dynein-dependent manner. *Genes Dev* **20**, 2421-2436 (2006).
4. Kume, K., Koyano, T., Kanai, M., Toda, T. & Hirata, D. Calcineurin ensures a link between the DNA replication checkpoint and microtubule-dependent polarized growth. *Nat Cell Biol* **13**, 234-242 (2011).
5. Tatebe, H., Nakano, K., Maximo, R. & Shiozaki, K. Pom1 DYRK regulates localization of the Rga4 GAP to ensure bipolar activation of Cdc42 in fission yeast. *Curr Biol* **18**, 322-330 (2008).
