# Supplementary material for: Ubiquitination of CLIP-170 family protein restrains polarized growth upon DNA replication stress
Source: Nat Commun. 2022 Sep 22;13:5565. doi: 10.1038/s41467-022-33311-y (PMC9499959; doi:10.1038/s41467-022-33311-y)
Supplement: Supplementary file 3 — Description of Additional Supplementary Files [file 41467_2022_33311_MOESM3_ESM.pdf]

Description of Additional Supplementary Files

File Name: Supplementary Data 1
Description: The list of potential Dma1-interacting proteins identified by mass spectrometry.
