# Supplementary material for: Ubiquitination of CLIP-170 family protein restrains polarized growth upon DNA replication stress
Source: Nat Commun. 2022 Sep 22;13:5565. doi: 10.1038/s41467-022-33311-y (PMC9499959; doi:10.1038/s41467-022-33311-y)
Supplement: Supplementary file 4 — Supplementary Data 1 [file 41467_2022_33311_MOESM4_ESM.pdf]

dma1 deletion
+ pREP41-NTAP-dma1-5xGly-Ub(7K->7R)

DTASelect v1.9
SEQUEST 2.7 in SQT format.
Jump to the summary table.

sequest.params modifications:

*	STY	0.0
#	C	0.0
@	M	0.0
Static	C	57.0

true	Use criteria
1.8	Minimum +1 XCorr
2.5	Minimum +2 XCorr
3.5	Minimum +3 XCorr
0.08	Minimum DeltCN
1	Minimum charge state
3	Maximum charge state
0.0	Minimum ion proportion
1000	Maximum Sp rank
-1.0	Minimum Sp score
Include	Modified peptide inclusion
Any	Tryptic status requirement
true	Multiple, ambiguous IDs allowed
Ignore	Peptide validation handling
XCorr	Purge duplicate peptides by protein
false	Include only loci with unique peptide
false	Remove subset proteins
Ignore	Locus validation handling
0	Minimum modified peptides per locus
10	Minimum redundancy for low coverage loci
2	Minimum peptides per locus

Locus Key:

Validation Status	Locus	Sequence Count	Spectrum Count	Sequence Coverage	Length	MolWt	pI	Descriptive Name

Similarity Key:

Locus	# of identical peptides	# of differing peptides

U **contaminant_gi|7463016|pir||S77957** 9 15 ? Not found in database

	Filename	XCorr	DeltCN	ObsM+H+	CalcM+H+	SpR	SpScore	Ion%	#	Sequence	
*	100303-DM1159-p620-03.0726.0726.1	1.8832	0.1792	725.38	725.82	1	522.1	75.0%	1	R.SVAAYSK.Q	1
*	100303-DM1159-p620-05.2400.2400.1	1.925	0.3267	1051.46	1052.175	1	582.2	60.0%	1	R.VLGQLHGGPSS.C	1
*	100303-DM1159-p620-05.2401.2401.2	2.9981	0.3438	1052.19	1052.175	1	681.3	80.0%	1	R.VLGQLHGGPSS.C	2
*	100303-DM1159-p620-04.1698.1698.2	3.8527	0.3941	1428.06	1428.544	1	1617.7	76.9%	2	R.VFTSWTGGGTSATR.L	2
*	100303-DM1159-p620-03.1212.1212.2	4.7051	0.5323	2093.98	2094.159	1	1483.2	56.8%	1	P.GSSSSGANGDGSLAQSQTGAVVR.A	2
*	100303-DM1159-p620-02.1294.1294.2	4.8308	0.5475	2191.4	2191.276	1	1056.7	54.3%	1	A.PGSSSSGANGDGSLAQSQTGAVVR.A	2
*	100303-DM1159-p620-03.1230.1230.2	5.3209	0.4504	2261.96	2262.355	1	1278.6	54.2%	1	R.APGSSSSGANGDGSLAQSQTGAVVR.A	2
*	100303-DM1159-p620-03.3715.3715.2	3.5658	0.2866	2505.8	2506.685	1	839.1	37.5%	1	R.LSDWLDAAGTGAQFIDGLDSTGTPP.V	2
*	100303-DM1159-p620-02.4119.4119.2	6.2335	0.4729	2605.93	2605.818	1	2253.0	56.0%	6	R.LSDWLDAAGTGAQFIDGLDSTGTPPV.-	2

U **contaminant_KERATIN22** 2 2 ? Not found in database

Filename	XCorr	DeltCN	ObsM+H+	CalcM+H+	SpR	SpScore	Ion%	#	Sequence	
100303-DM1159-p620-03.1494.1494.1	1.8916	0.1826	973.38	974.102	1	309.6	78.6%	1	K.IEISELNR.V	11
100303-DM1159-p620-03.1471.1471.2	2.7488	0.153	1478.83	1476.673	1	1270.2	72.7%	1	R.FLEQQNQVLQTK.W	22

Similarities: contaminant_KERATIN13(2:0)

U **contaminant_KERATIN13** 6 6 ? Not found in database

	Filename	XCorr	DeltCN	ObsM+H+	CalcM+H+	SpR	SpScore	Ion%	#	Sequence	
	100303-DM1159-p620-03.1494.1494.1	1.8916	0.1826	973.38	974.102	1	309.6	78.6%	1	K.IEISELNR.V	11
*	100303-DM1159-p620-03.1147.1147.2	3.0875	0.3422	1126.15	1126.208	1	906.0	77.8%	1	K.AEAESLYQSK.Y	2
*	100303-DM1159-p620-03.1640.1640.2	2.7208	0.2272	1266.25	1266.393	2	664.6	70.0%	1	R.TNAENEFVTIK.K	2

*	100303-DM1159-p620-03.2395.2395.2	2.8868	0.2854	1385.31	1384.532	2	575.2	59.1%	1	K.SLNNQFASFIDK.V	2
	100303-DM1159-p620-03.1471.1471.2	2.7488	0.153	1478.83	1476.673	1	1270.2	72.7%	1	R.FLEQQNQVLQTK.W	22
*	100303-DM1159-p620-03.1162.1162.2	5.7814	0.3264	2383.98	2385.298	1	1103.6	40.0%	1	R.GGGGGGYGSGGSSYGSGGGSYGSGGGGGGGR.G	2

Similarities: contaminant_KERATIN22(2:4)

U **contaminant_KERATIN06** 2 2 ? Not found in database

	Filename	XCorr	DeltCN	ObsM+H+	CalcM+H+	SpR	SpScore	Ion%	#	Sequence	
	100303-DM1159-p620-03.1871.1871.1	1.9048	0.1296	1029.61	1030.21	29	250.3	62.5%	1	R.VLDELTLAR.A	11
*	100303-DM1159-p620-04.1554.1554.2	3.269	0.1262	1204.74	1203.381	5	879.7	70.0%	1	M.ATTIDNSRVIL.E	2

Similarities: contaminant_KERATIN05(1:1)

U **KAP123,SPBC14F5.03C** 2 3 ? Not found in database

	Filename	XCorr	DeltCN	ObsM+H+	CalcM+H+	SpR	SpScore	Ion%	#	Sequence	
*	100303-DM1159-p620-02.1691.1691.1	2.0044	0.143	840.34	840.819	87	77.3	50.0%	2	D.DDEGYEL.R	1
*	100303-DM1159-p620-05.2260.2260.2	2.8448	0.1624	1215.51	1215.436	25	664.7	58.3%	1	I.ASGAIISKALGNI.I	2

U **contaminant_KERATIN05** 2 2 ? Not found in database

	Filename	XCorr	DeltCN	ObsM+H+	CalcM+H+	SpR	SpScore	Ion%	#	Sequence	
	100303-DM1159-p620-03.1871.1871.1	1.9048	0.1296	1029.61	1030.21	29	250.3	62.5%	1	R.VLDELTLAR.A	11
*	100303-DM1159-p620-02.2302.2302.2	2.5319	0.1508	1565.57	1564.819	5	481.4	54.2%	1	M.QIESLKEELAYLK.K	2

Similarities: contaminant_KERATIN06(1:1)

U **PGK1,SPBC14F5.04C** 4 4 ? Not found in database

	Filename	XCorr	DeltCN	ObsM+H+	CalcM+H+	SpR	SpScore	Ion%	#	Sequence	
*	100303-DM1159-p620-05.2802.2802.1	2.0127	0.089	913.59	912.161	1	507.9	62.5%	1	R.IVGALPTIK.Y	1
*	100303-DM1159-p620-05.2873.2873.1	1.8916	0.1049	1041.47	1042.264	1	637.0	65.0%	1	K.ALPGVVALSSK.-	1
*	100303-DM1159-p620-03.1490.1490.2	5.3667	0.5287	1920.82	1920.093	1	1529.5	68.4%	1	K.TCEAGNVVIVGGGDTATVAK.K	2
*	100303-DM1159-p620-03.2599.2599.2	3.0893	0.3152	2247.41	2248.435	1	376.6	47.6%	1	K.VGSATAEEGIPDGWMGLDCGPK.S	2

U **contaminant_KERATIN02** 3 3 ? Not found in database

	Filename	XCorr	DeltCN	ObsM+H+	CalcM+H+	SpR	SpScore	Ion%	#	Sequence	
*	100303-DM1159-p620-03.1744.1744.1	1.8612	0.0849	1060.52	1061.18	26	223.0	56.2%	1	K.TLLDIDNTR.M	1
*	100303-DM1159-p620-04.1127.1127.2	2.8528	0.4248	1232.66	1233.283	1	996.0	66.7%	1	T.SGGGGGGGLGSGGSIR.S	2
*	100303-DM1159-p620-03.1038.1038.2	2.6729	0.3143	1235.84	1236.24	1	849.3	70.8%	1	R.FSSSSGYGGGSSR.V	2

U **RPS3,SPBC16G5.14C** 2 2 ? Not found in database

	Filename	XCorr	DeltCN	ObsM+H+	CalcM+H+	SpR	SpScore	Ion%	#	Sequence	
*	100303-DM1159-p620-03.1258.1258.2	2.7617	0.3348	1515.33	1515.54	1	1057.3	75.0%	1	R.ELSEEGYSGCEVR.V	2
*	100303-DM1159-p620-02.2006.2006.2	2.512	0.0877	1721.52	1722.004	17	388.9	42.9%	1	A.QCESLRYKLLAGLAV.R	2

U **contaminant_GR78_SCHPO** 2 2 ? Not found in database

	Filename	XCorr	DeltCN	ObsM+H+	CalcM+H+	SpR	SpScore	Ion%	#	Sequence	
	100303-DM1159-p620-03.1896.1896.2	2.9012	0.41	1528.7	1528.659	1	692.4	66.7%	1	R.ITPSYVAFTEDER.L	22
	100303-DM1159-p620-04.3334.3334.2	5.1456	0.5639	2122.68	2120.28	1	1767.9	76.5%	1	R.IEIESFFNGQDFSETLSR.A	22

Similarities: BIP,SPAC22A12.15C(2:0)

U **SPF38,SPBC1289.11** 2 2 ? Not found in database

	Filename	XCorr	DeltCN	ObsM+H+	CalcM+H+	SpR	SpScore	Ion%	#	Sequence	
*	100303-DM1159-p620-03.1916.1916.2	3.262	0.3467	1596.89	1594.695	1	462.8	60.7%	1	R.FDPSGSYFASGGMDR.Q	2
*	100303-DM1159-p620-03.1780.1780.2	2.8052	0.3667	1597.27	1596.709	1	768.7	60.7%	1	K.DGSSLLSNSMDNTVR.I	2

U **DMA1,SPAC17G8.10C** 26 94 48.3% 267 30597 6.9 : SPINDLE ASSEMBLY CHECKPOINT COMPONENT; PROTEIN TO PREVENT SEPTUM FORMATION AND PREMATURE EXIT FROM MITOSIS IF SPINDLE FUNCTION IS COMPROMISED: Q10322;

	Filename	XCorr	DeltCN	ObsM+H+	CalcM+H+	SpR	SpScore	Ion%	#	Sequence	
*	100303-DM1159-p620-03.1370.1370.2	2.7821	0.192	1052.97	1053.157	1	961.5	81.2%	1	K.SVEGYLKEQ.E	2
*	100303-DM1159-p620-03.0526.0526.2	3.4645	0.4748	1823.72	1823.819	1	544.9	46.7%	1	K.EQELAAETDSEKDDDK.I	2
*	100303-DM1159-p620-03.1362.1362.3	3.7715	0.329	2293.18	2293.403	1	588.5	32.9%	1	K.EQELAAETDSEKDDDKISIR.L	3
*	100303-DM1159-p620-05.2582.2582.2	5.2431	0.4564	2293.59	2293.403	1	1059.7	52.6%	14	K.EQELAAETDSEKDDDKISIR.L	2

*	100303-DM1159-p620-04.1411.1411.2	3.304	0.334	2409.16	2406.563	1	855.5	45.0%	1	K.EQELAAETDSEKDDDKISIRL.T	2
*	100303-DM1159-p620-04.1327.1327.2	4.8084	0.4817	2036.34	2036.157	1	1931.2	67.6%	1	Q.ELAAETDSEKDDDKISIR.L	2
*	100303-DM1159-p620-04.1414.1414.2	3.2491	0.3432	1070.39	1070.193	1	1087.1	94.4%	1	R.LTNFVGPNAH.S	2
*	100303-DM1159-p620-04.1420.1420.1	1.9889	0.2284	1071.38	1070.193	1	301.6	61.1%	2	R.LTNFVGPNAH.S	1
*	100303-DM1159-p620-05.2922.2922.2	3.2004	0.1707	1304.48	1304.448	1	1069.5	81.8%	1	R.LTNFVGPNAHSF.S	2
*	100303-DM1159-p620-04.1916.1916.2	2.7937	0.292	1472.64	1472.734	1	847.6	72.7%	1	N.RKQNNLPIYIGR.Y	2
*	100303-DM1159-p620-04.1914.1914.1	2.2563	0.1099	1187.58	1188.372	126	58.1	55.6%	1	K.QNNLPIYIGR.Y	1
*	100303-DM1159-p620-04.1946.1946.2	2.6788	0.2242	1187.84	1188.372	4	744.9	88.9%	1	K.QNNLPIYIGR.Y	2
*	100303-DM1159-p620-03.2178.2178.1	2.2277	0.2881	1298.54	1298.441	1	257.3	59.1%	8	R.YNGGDVSAIVFR.S	1
*	100303-DM1159-p620-03.2163.2163.2	3.8635	0.3407	1301.35	1298.441	1	1626.7	86.4%	11	R.YNGGDVSAIVFR.S	2
*	100303-DM1159-p620-02.2572.2572.2	3.5645	0.4849	1136.15	1135.265	1	1326.9	90.0%	2	Y.NGGDVSAIVFR.S	2
*	100303-DM1159-p620-02.2580.2580.1	2.4942	0.3029	1136.63	1135.265	4	257.4	60.0%	1	Y.NGGDVSAIVFR.S	1
*	100303-DM1159-p620-02.2508.2508.1	2.055	0.2059	1020.54	1021.161	29	258.3	61.1%	5	N.GGDVSAIVFR.S	1
*	100303-DM1159-p620-04.2719.2719.2	5.4208	0.4784	1952.09	1950.158	1	1355.3	75.0%	5	K.PYPISNNDILQLGADYR.G	2
*	100303-DM1159-p620-04.0880.0880.1	2.1962	0.1334	933.36	931.983	1	328.2	64.3%	8	R.GGHEVNYR.C	1
*	100303-DM1159-p620-05.2616.2616.2	3.4354	0.2868	1218.59	1217.37	1	881.1	83.3%	4	R.ARVELNNSWK.I	2
*	100303-DM1159-p620-03.1382.1382.2	3.0463	0.126	989.46	990.104	1	697.3	92.9%	1	R.VELNNSWK.I	2
*	100303-DM1159-p620-03.1431.1431.1	2.0011	0.2012	990.4	990.104	1	366.3	78.6%	2	R.VELNNSWK.I	1
*	100303-DM1159-p620-05.2788.2788.2	2.6616	0.244	1382.4	1381.574	3	382.1	70.0%	1	K.LSPYNLNEFKR.M	2
*	100303-DM1159-p620-05.2792.2792.1	1.9173	0.1824	1382.83	1381.574	1	273.1	60.0%	1	K.LSPYNLNEFKR.M	1
*	100303-DM1159-p620-03.1999.1999.2	2.9537	0.1932	1024.85	1025.149	5	639.1	85.7%	1	S.PYNLNEFK.R	2
*	100303-DM1159-p620-03.2324.2324.2	5.93	0.5104	2317.47	2315.455	1	1764.3	71.1%	18	K.YHDLEAPVEEGDESLNDLLR.N	2

U **RPL31,SPAC890.08** 8 13 47.8% 113 13261 10.2 : 60S RIBOSOMAL PROTEIN L31: O14384;Q9URX6;

	Filename	XCorr	DeltCN	ObsM+H+	CalcM+H+	SpR	SpScore	Ion%	#	Sequence	
*	100303-DM1159-p620-05.2272.2272.1	2.1375	0.2214	1115.58	1116.306	1	174.2	61.1%	1	K.KSAINQVVTR.D	1
*	100303-DM1159-p620-05.2268.2268.2	3.2324	0.4243	1116.56	1116.306	1	878.2	83.3%	1	K.KSAINQVVTR.D	2
*	100303-DM1159-p620-03.1222.1222.2	3.1433	0.3875	987.75	988.132	1	1213.7	87.5%	2	K.SAINQVVTR.D	2
*	100303-DM1159-p620-05.3653.3653.2	3.3038	0.3599	1375.45	1374.669	1	1287.8	77.3%	2	P.RAIKEIVAFAQK.H	2
*	100303-DM1159-p620-03.1435.1435.1	2.7183	0.1126	907.4	906.07	1	347.1	71.4%	2	K.EIVAFAQK.H	1
*	100303-DM1159-p620-05.2689.2689.2	2.5486	0.248	1314.95	1315.512	2	279.7	75.0%	1	R.VDPSLNKEVWK.R	2
*	100303-DM1159-p620-05.3269.3269.2	6.1818	0.617	2342.19	2342.521	1	2532.3	72.5%	3	R.SDEDDKALYTYVQAVDVANPK.M	2
*	100303-DM1159-p620-04.2203.2203.2	4.5083	0.3419	1655.28	1652.888	1	997.1	64.3%	1	K.ALYTYVQAVDVANPK.M	2

U **RPS17-1,SPBC839.05C** 6 8 39.7% 131 15514 10.3 : 40S RIBOSOMAL PROTEIN S17: ;N0ACC;

	Filename	XCorr	DeltCN	ObsM+H+	CalcM+H+	SpR	SpScore	Ion%	#	Sequence	
	100303-DM1159-p620-04.1858.1858.2	2.7248	0.2361	1080.08	1080.226	1	820.0	87.5%	1	R.LTLDFQTNK.R	22
	100303-DM1159-p620-05.2910.2910.1	2.4312	0.1746	1080.49	1080.226	3	316.0	68.8%	1	R.LTLDFQTNK.R	11
	100303-DM1159-p620-03.1842.1842.2	3.8222	0.3949	1158.58	1158.381	1	1170.5	90.0%	3	R.IVDEVAIIASK.R	22
	100303-DM1159-p620-04.1892.1892.1	2.352	0.1596	1159.5	1158.381	1	662.1	65.0%	1	R.IVDEVAIIASK.R	11
*	100303-DM1159-p620-05.3138.3138.2	3.4029	0.315	2578.93	2578.749	1	475.9	42.9%	1	K.DQYVPEVSELEVDRVNVDQDTK.D	2
*	100303-DM1159-p620-03.1687.1687.1	1.9414	0.1378	1147.6	1148.304	1	317.7	66.7%	1	K.SLGYDQIPVR.V	1

Similarities: RPS17-2,SPCC24B10.09(4:2)

U **RPL23-1,SPAC3G9.03** 3 7 34.5% 139 14882 10.2 : 60S RIBOSOMAL PROTEIN L23: O42867;

U **RPL23-2,SPCC1322.11** 3 7 34.5% 139 14882 10.2 : 60S RIBOSOMAL PROTEIN L23: O42867;

Filename	XCorr	DeltCN	ObsM+H+	CalcM+H+	SpR	SpScore	Ion%	#	Sequence	
100303-DM1159-p620-05.3589.3589.2	4.3307	0.5231	2091.54	2092.399	1	935.7	63.2%	3	R.MTLGLPVQAIMNCADNSGAK.N	2
100303-DM1159-p620-04.3111.3111.2	3.4086	0.2733	1398.31	1397.617	1	1515.5	75.0%	1	K.NLYIVSVFGTGAR.L	2
100303-DM1159-p620-04.1975.1975.2	3.8805	0.583	1533.14	1533.79	1	1397.9	71.4%	3	R.LPAASCGDMVLATVK.K	2

U **RPL38-1,SPBC577.02** 2 3 33.8% 74 8492 10.2 : 60S RIBOSOMAL PROTEIN L38: Q9USR7;

	Filename	XCorr	DeltCN	ObsM+H+	CalcM+H+	SpR	SpScore	Ion%	#	Sequence	
	100303-DM1159-p620-05.3201.3201.1	2.1577	0.153	1255.56	1256.485	1	907.2	65.0%	2	K.YLYTLVVADAK.K	11
*	100303-DM1159-p620-03.1778.1778.2	3.0521	0.3288	1484.51	1484.69	1	543.1	61.5%	1	R.QSLPPDLTVTEVGK.K	2

Similarities: RPL38-2,SPAC30D11.12(1:1)

U **RPL38-2,SPAC30D11.12** 2 3 33.8% 74 8339 10.5 : 60S RIBOSOMAL PROTEIN L38: Q09900;

	Filename	XCorr	DeltCN	ObsM+H+	CalcM+H+	SpR	SpScore	Ion%	#	Sequence	
	100303-DM1159-p620-05.3201.3201.1	2.1577	0.153	1255.56	1256.485	1	907.2	65.0%	2	R.YLYTLVVADAK.K	11
*	100303-DM1159-p620-03.1870.1870.2	2.6463	0.3116	1440.56	1440.68	5	323.8	53.8%	1	R.QSLPPALTVTEVGK.K	2

Similarities: RPL38-1,SPBC577.02(1:1)

U **RPS13,SPAC6F6.07C** 5 6 32.5% 151 16953 10.4 : 40S RIBOSOMAL PROTEIN S13: P28189;

	Filename	XCorr	DeltCN	ObsM+H+	CalcM+H+	SpR	SpScore	Ion%	#	Sequence	
*	100303-DM1159-p620-05.2906.2906.1	1.8328	0.1763	1133.65	1134.321	99	89.3	40.0%	1	K.GIASSALPYVR.S	1
*	100303-DM1159-p620-04.1846.1846.2	3.3377	0.4379	1133.98	1134.321	1	894.9	80.0%	1	K.GIASSALPYVR.S	2
*	100303-DM1159-p620-03.2678.2678.2	3.9115	0.2773	1288.46	1288.44	1	1490.4	86.4%	1	K.ADADSVVEQILK.F	2
*	100303-DM1159-p620-05.3925.3925.2	3.4106	0.3667	1871.71	1871.141	1	834.5	65.6%	2	K.ANGLAPELPEDLYNLIK.K	2
*	100303-DM1159-p620-05.2786.2786.2	2.5064	0.2771	969.25	969.172	7	395.8	75.0%	1	K.VGALPPTWK.Y	2

U **RPS29,SPBC1685.09** 4 6 30.4% 56 6658 10.7 : 40S RIBOSOMAL PROTEIN S29: O74329;

	Filename	XCorr	DeltCN	ObsM+H+	CalcM+H+	SpR	SpScore	Ion%	#	Sequence	
*	100303-DM1159-p620-04.1467.1467.1	1.9391	0.1022	822.32	822.939	33	129.9	66.7%	1	K.YGLNISR.Q	1
*	100303-DM1159-p620-04.1470.1470.2	2.5502	0.1591	822.35	822.939	4	801.6	83.3%	1	K.YGLNISR.Q	2
*	100303-DM1159-p620-03.1718.1718.2	2.5824	0.2653	1156.2	1156.28	12	356.4	50.0%	1	R.EYANDIGFVK.Y	2
*	100303-DM1159-p620-03.1807.1807.1	2.8579	0.3511	1156.54	1156.28	1	231.3	66.7%	3	R.EYANDIGFVK.Y	1

U **RPS18-1,SPBC16D10.11C** 7 10 29.6% 152 17435 10.5 : 40S RIBOSOMAL PROTEIN S18: O94754;

U **RPS18-2,SPCC1259.01C,SPCC825.06C** 7 10 29.6% 152 17435 10.5 : RIBOSOMAL PROTEIN SUBUNIT S18 40S RIBOSOMAL PROTEIN S18: O94754;Q9USH4;

Filename	XCorr	DeltCN	ObsM+H+	CalcM+H+	SpR	SpScore	Ion%	#	Sequence	
100303-DM1159-p620-03.1191.1191.1	2.1639	0.2289	973.49	974.102	1	367.1	68.8%	1	R.LLNTNVDGK.V	1
100303-DM1159-p620-04.1352.1352.2	3.1548	0.3147	1403.92	1404.52	1	626.4	77.3%	1	K.RAGELTTEELER.I	2
100303-DM1159-p620-03.1350.1350.2	3.3252	0.3205	1248.34	1248.332	1	1211.2	85.0%	1	R.AGELTTEELER.I	2
100303-DM1159-p620-05.2974.2974.2	4.6119	0.4175	1389.43	1388.65	1	2023.4	86.4%	2	R.IVTIIQNPSQFK.I	2
100303-DM1159-p620-05.2980.2980.1	3.4924	0.277	1389.55	1388.65	2	516.5	54.5%	2	R.IVTIIQNPSQFK.I	1
100303-DM1159-p620-03.1886.1886.2	3.9257	0.1466	1335.62	1336.488	1	1411.5	86.4%	2	K.SFQLLANNVDSK.L	2
100303-DM1159-p620-03.1891.1891.1	3.5649	0.207	1336.55	1336.488	1	820.2	72.7%	1	K.SFQLLANNVDSK.L	1

U **RPL37A-2,SPBC83.02C,RPL43-2** 3 5 28.7% 94 10380 11.0 : 60S RIBOSOMAL PROTEIN L37A/L43: O13673;O94686;

	Filename	XCorr	DeltCN	ObsM+H+	CalcM+H+	SpR	SpScore	Ion%	#	Sequence	
	100303-DM1159-p620-05.1858.1858.1	1.9193	0.1109	1124.77	1125.273	1	199.6	62.5%	1	R.KIEVQQHSR.Y	11
	100303-DM1159-p620-05.1824.1824.2	2.8993	0.3235	1124.87	1125.273	1	1004.5	87.5%	3	R.KIEVQQHSR.Y	22
*	100303-DM1159-p620-05.3024.3024.2	4.2171	0.4764	1708.79	1706.896	1	612.2	52.9%	1	K.TLAGGAWTVTTAAATSAR.S	2

Similarities: RPL37A-1,SPBC800.04C,RPL43-1(2:1)

U **PFK1,SPBC16H5.02** 28 58 28.6% 942 102555 6.2 : 6-PHOSPHOFRUCTOKINASE BETA SUBUNIT; SIMILAR TO S. CEREVISIAE PFK2: O42938;

	Filename	XCorr	DeltCN	ObsM+H+	CalcM+H+	SpR	SpScore	Ion%	#	Sequence	
*	100303-DM1159-p620-04.1126.1126.2	3.4346	0.4475	1455.45	1455.524	1	1318.8	73.1%	1	K.KVASFGTSDSDNAR.V	2
*	100303-DM1159-p620-03.1070.1070.2	3.3668	0.302	1327.18	1327.35	1	1118.1	75.0%	2	K.VASFGTSDSDNAR.V	2
*	100303-DM1159-p620-04.2487.2487.2	4.3039	0.4246	1959.45	1957.147	1	898.4	73.5%	6	R.LVPGELSLSPAAEDSEWR.G	2
*	100303-DM1159-p620-02.3248.3248.2	3.3612	0.2817	1844.42	1843.988	2	431.9	46.9%	1	L.VPGELSLSPAAEDSEWR.G	2
*	100303-DM1159-p620-04.2483.2483.2	4.5632	0.4339	1744.62	1744.855	1	1026.3	73.3%	3	V.PGELSLSPAAEDSEWR.G	2
*	100303-DM1159-p620-02.3239.3239.1	1.8329	0.1865	1061.55	1061.096	4	308.6	62.5%	1	S.PAAEDSEWR.G	1
*	100303-DM1159-p620-03.1252.1252.1	2.0833	0.1767	996.41	997.095	1	502.4	78.6%	1	K.YQAFPNEK.K	1
*	100303-DM1159-p620-04.2624.2624.2	3.8879	0.4454	2289.95	2290.486	1	744.5	45.5%	1	K.KSEESGAASNLESGLATPVVETL.K	2
*	100303-DM1159-p620-03.2582.2582.2	6.2874	0.5632	2289.74	2290.486	1	1947.7	61.4%	3	K.SEESGAASNLESGLATPVVETLK.K	2
*	100303-DM1159-p620-03.1726.1726.2	5.6219	0.597	1762.5	1763.037	1	1891.7	67.6%	2	K.IAVMTSGGDSPGMNAVVR.A	2
*	100303-DM1159-p620-04.3454.3454.2	5.0372	0.4952	2393.84	2394.624	1	1496.4	59.5%	3	R.GCDAFAIYEGYEGLVQGGDMIK.Q	2

*	100303-DM1159-p620-03.1695.1695.2	2.7046	0.3077	1002.49	1002.118	1	742.4	92.9%	1	K.QLQWGDVR.G	2
*	100303-DM1159-p620-05.3280.3280.2	5.2018	0.4404	1401.82	1402.593	1	2929.1	88.5%	2	R.GWLAEGGTLIGTAR.C	2
*	100303-DM1159-p620-05.3200.3200.1	2.2015	0.2742	1021.61	1022.251	3	336.8	68.8%	1	R.AFIVEVMGR.H	1
*	100303-DM1159-p620-03.2006.2006.2	4.0834	0.4612	1594.54	1594.687	1	1523.2	83.3%	3	K.WQDELCNSLSSVR.K	2
*	100303-DM1159-p620-05.2129.2129.2	2.5568	0.3482	1009.65	1011.169	1	863.8	81.2%	1	R.VTTLGHVQR.G	2
*	100303-DM1159-p620-05.2353.2353.1	2.3043	0.2597	915.55	916.167	1	408.3	71.4%	1	R.KPLMEAVK.L	1
*	100303-DM1159-p620-04.1322.1322.2	3.0197	0.4327	1226.5	1226.372	1	1016.9	80.0%	2	K.LTHEVADAIEK.K	2
*	100303-DM1159-p620-03.2739.2739.2	3.3604	0.5106	1458.1	1458.626	1	761.3	79.2%	2	R.DTPDLDMGFTAFK.F	2
*	100303-DM1159-p620-03.3619.3619.2	2.8207	0.287	2242.81	2243.623	1	734.4	47.6%	1	R.IPMAIIPATISNNVPGTEFSLG.C	2
*	100303-DM1159-p620-03.1319.1319.1	1.8214	0.2337	1150.5	1151.274	18	227.0	55.6%	1	R.VFVCEVQGGR.S	1
*	100303-DM1159-p620-03.1315.1315.2	2.6401	0.1865	1151.29	1151.274	1	1487.8	94.4%	1	R.VFVCEVQGGR.S	2
*	100303-DM1159-p620-02.2059.2059.1	2.1134	0.1577	1052.43	1052.141	1	274.6	68.8%	1	V.FVCEVQGGR.S	1
*	100303-DM1159-p620-02.2060.2060.1	2.2702	0.2269	905.33	904.965	66	253.5	64.3%	1	F.VCEVQGGR.S	1
*	100303-DM1159-p620-03.1515.1515.1	2.293	0.2364	935.46	936.056	2	312.1	68.8%	4	K.ATFALEAGR.N	1
*	100303-DM1159-p620-03.1578.1578.1	1.9458	0.1616	708.36	708.789	16	335.7	66.7%	1	A.TFALEAG.R	1
*	100303-DM1159-p620-03.1906.1906.2	5.457	0.5136	1698.71	1698.874	1	1674.1	78.1%	5	R.ANDLGNDPSSAVVIGIR.G	2
*	100303-DM1159-p620-05.3213.3213.2	5.9462	0.4903	2372.62	2372.523	1	1721.8	61.9%	6	R.GTGVSFSSVADVENNETEIEMR.R	2

U **RPL36-1,SPCC970.05** 3 4 28.3% 99 11263 11.8 : 60S RIBOSOMAL PROTEIN L36.1/L36A: Q92365;
U **RPL36-2,SPBC405.07** 3 4 28.3% 99 11258 11.8 : 60S RIBOSOMAL PROTEIN L36: O94658;

Filename	XCorr	DeltCN	ObsM+H+	CalcM+H+	SpR	SpScore	Ion%	#	Sequence	
100303-DM1159-p620-05.2766.2766.1	2.2911	0.2824	967.64	968.184	1	537.4	66.7%	1	M.APGLVVGLNK.G	1
100303-DM1159-p620-03.1591.1591.1	1.8821	0.0971	760.47	760.97	43	165.9	80.0%	1	R.VMELIR.N	1
100303-DM1159-p620-03.1846.1846.2	4.3656	0.3577	1362.57	1362.523	1	1905.5	81.8%	2	K.IEELTSVIQSSR.L	2

U **RPL35,SPCC613.05C** 3 3 27.9% 122 14302 10.7 : 60S RIBOSOMAL PROTEIN L35: O74904;

	Filename	XCorr	DeltCN	ObsM+H+	CalcM+H+	SpR	SpScore	Ion%	#	Sequence	
*	100303-DM1159-p620-03.2290.2290.2	3.7121	0.3397	1687.32	1686.819	1	1131.6	73.1%	1	K.QSQENLAEQLQELR.Q	2
*	100303-DM1159-p620-03.1483.1483.2	3.2832	0.3487	1158.84	1159.328	1	1276.4	88.9%	1	R.ILTVINESNR.L	2
*	100303-DM1159-p620-05.2309.2309.2	3.3325	0.3311	1221.45	1221.359	1	942.8	77.8%	1	R.RALTPYEQSR.K	2

U **RPS15A-1,SPAC22A12.04C,RPS22-1** 3 4 27.7% 130 14760 10.3 : 40S RIBOSOMAL PROTEIN S15A/S22A: O14469;
U **RPS15A-2,SPAC5D6.01,RPS22-2** 3 4 27.7% 130 14760 10.3 : 40S RIBOSOMAL PROTEIN S15A(S22): O14469;

Filename	XCorr	DeltCN	ObsM+H+	CalcM+H+	SpR	SpScore	Ion%	#	Sequence	
100303-DM1159-p620-03.1980.1980.2	4.1937	0.5557	1817.59	1816.975	1	1221.1	66.7%	2	R.QSVLADCLNNIVNAER.R	2
100303-DM1159-p620-05.3045.3045.2	2.9783	0.1183	1112.49	1113.305	1	1149.1	87.5%	1	K.WVNQLLPSR.Q	2
100303-DM1159-p620-05.2902.2902.2	2.8626	0.2755	1172.49	1173.398	3	415.7	60.0%	1	R.QVGVIVLTTSR.G	2

U **RPS7,SPAC18G6.14C** 3 14 26.7% 195 21947 10.1 : 40S RIBOSOMAL PROTEIN S7: Q10101;

	Filename	XCorr	DeltCN	ObsM+H+	CalcM+H+	SpR	SpScore	Ion%	#	Sequence	
*	100303-DM1159-p620-04.3355.3355.2	5.5517	0.4834	2661.21	2659.827	1	819.3	50.0%	11	R.SSSQPTETDLLVAQCLYDLESSSK.D	2
*	100303-DM1159-p620-05.2726.2726.2	3.3716	0.4306	1670.35	1670.819	1	690.0	73.1%	1	K.VFLDNRDANTVDYK.L	2
*	100303-DM1159-p620-02.3238.3238.2	3.1938	0.3089	1480.81	1481.645	2	444.0	53.8%	2	K.NVTFEFPVATGEGL.-	2

U **RPL28-2,SPCC5E4.07** 5 8 25.7% 148 16653 11.1 : 60S RIBOSOMAL PROTEIN L27A.2/L28A: P36585;

	Filename	XCorr	DeltCN	ObsM+H+	CalcM+H+	SpR	SpScore	Ion%	#	Sequence	
*	100303-DM1159-p620-04.2574.2574.2	2.6712	0.1796	1212.9	1213.422	12	541.1	72.2%	1	R.LWTLLPNEAR.D	2
	100303-DM1159-p620-04.2576.2576.1	1.8129	0.0985	586.36	586.625	7	303.7	87.5%	1	L.PNEAR.D	1
	100303-DM1159-p620-04.2522.2522.2	5.2146	0.537	1789.76	1790.026	1	1410.1	78.1%	3	K.NTEVAPVINVLQSGYGK.V	22
*	100303-DM1159-p620-04.1699.1699.1	1.9215	0.213	1252.61	1253.484	1	358.3	70.0%	1	R.LPETPVIVQTR.Y	1
*	100303-DM1159-p620-04.1691.1691.2	3.2969	0.2098	1253.09	1253.484	1	798.3	80.0%	2	R.LPETPVIVQTR.Y	2

Similarities: RPL28-1,SPBC776.11(1:4)

U **RPL26,SPBC29B5.03C** 3 4 25.4% 126 14341 11.2 : 60S RIBOSOMAL PROTEIN L26: P78946;

Filename	XCorr	DeltCN	ObsM+H+	CalcM+H+	SpR	SpScore	Ion%	#	Sequence

*	100303-DM1159-p620-04.1516.1516.1	1.8343	0.0846	947.5	946.193	92	197.0	50.0%	1	R.VLMSAPLSK.E	1
*	100303-DM1159-p620-03.1292.1292.2	3.1664	0.283	1116.56	1116.263	1	1448.9	93.8%	2	R.RDDQITVIR.G	2
*	100303-DM1159-p620-03.1158.1158.2	3.0956	0.3519	1259.13	1258.374	1	819.5	76.9%	1	K.ANGASAPVGIDASK.V	2

U **SMD2,SPAC2C4.03C,CWF9** 3 5 25.2% 115 13095 9.7 : SMALL NUCLEAR RIBONUCLEOPROTEIN; PUTATIVE PRE- MRNA SPLICING FACTOR; SIMILAR TO S. CEREVISIAE SMD2: O14036;

	Filename	XCorr	DeltCN	ObsM+H+	CalcM+H+	SpR	SpScore	Ion%	#	Sequence	
*	100303-DM1159-p620-03.1816.1816.1	2.0339	0.1258	1146.47	1147.271	24	226.5	55.6%	1	R.SELSEIELAR.L	1
*	100303-DM1159-p620-03.1815.1815.2	3.7002	0.2585	1147.38	1147.271	1	1781.2	94.4%	1	R.SELSEIELAR.L	2
*	100303-DM1159-p620-05.3834.3834.2	3.8004	0.3739	2107.92	2109.384	1	982.4	58.3%	3	R.LEEYEFSAGPLSVLQQAVK.N	2

U **RPS17-2,SPCC24B10.09** 5 7 25.0% 132 15534 10.1 : 40S RIBOSOMAL PROTEIN S17: Q9P7J6;

	Filename	XCorr	DeltCN	ObsM+H+	CalcM+H+	SpR	SpScore	Ion%	#	Sequence	
	100303-DM1159-p620-04.1858.1858.2	2.7248	0.2361	1080.08	1080.226	1	820.0	87.5%	1	R.LTLDFQTNK.R	22
	100303-DM1159-p620-05.2910.2910.1	2.4312	0.1746	1080.49	1080.226	3	316.0	68.8%	1	R.LTLDFQTNK.R	11
	100303-DM1159-p620-03.1842.1842.2	3.8222	0.3949	1158.58	1158.381	1	1170.5	90.0%	3	R.IVDEVAIIASK.R	22
	100303-DM1159-p620-04.1892.1892.1	2.352	0.1596	1159.5	1158.381	1	662.1	65.0%	1	R.IVDEVAIIASK.R	11
*	100303-DM1159-p620-05.2810.2810.2	4.566	0.2302	1565.19	1564.733	1	1121.5	87.5%	1	R.KDQYVPEVSELEK.D	2

Similarities: RPS17-1,SPBC839.05C(4:1)

U **RPL13,SPAC664.05** 5 15 24.5% 208 23529 10.7 : 60S RIBOSOMAL PROTEIN L13: O74175;

	Filename	XCorr	DeltCN	ObsM+H+	CalcM+H+	SpR	SpScore	Ion%	#	Sequence	
*	100303-DM1159-p620-04.1727.1727.1	2.368	0.0817	1007.54	1006.109	2	201.2	71.4%	3	K.TWFNQPGR.K	1
*	100303-DM1159-p620-05.2633.2633.2	3.56	0.5057	1268.18	1265.455	1	674.0	72.7%	2	R.VASTIGIPVDHR.R	2
*	100303-DM1159-p620-03.0786.0786.2	2.5555	0.1948	1119.2	1119.18	5	664.2	81.2%	1	R.NRSEESLQR.N	2
*	100303-DM1159-p620-02.1388.1388.1	1.861	0.3524	1026.44	1027.206	4	188.1	55.6%	1	E.QTDVAAVLPI.T	1
*	100303-DM1159-p620-04.2098.2098.2	4.0141	0.4109	1400.26	1400.491	1	2077.1	86.4%	8	K.NFNAFSTLSNER.A	2

U **RPS5,SPAC8C9.08** 4 4 24.1% 203 22225 9.9 : 40S RIBOSOMAL PROTEIN S5: O14277;

	Filename	XCorr	DeltCN	ObsM+H+	CalcM+H+	SpR	SpScore	Ion%	#	Sequence	
*	100303-DM1159-p620-02.1914.1914.1	2.6001	0.2696	1216.44	1216.334	1	448.8	68.2%	1	T.PGVSLDENGSIK.L	1
	100303-DM1159-p620-05.1822.1822.2	2.604	0.3216	917.03	917.056	1	817.9	81.2%	1	T.RIGSAGTVR.R	22
	100303-DM1159-p620-05.3401.3401.2	2.596	0.27	1341.04	1340.609	1	1529.2	75.0%	1	R.VNQALALITIGAR.E	22
	100303-DM1159-p620-05.3716.3716.2	2.5254	0.1856	1648.35	1648.817	1	774.7	53.6%	1	K.SISECLAEEIINAAK.G	22

Similarities: RPS5-2,SPAC328.10C(3:1)

U **RPS11-1,SPAC31G5.03** 4 4 22.4% 152 17500 10.4 : 40S RIBOSOMAL PROTEIN S11: P79013;

U **RPS11-2,SPAC144.11** 4 4 22.4% 152 17500 10.4 : 40S RIBOSOMAL PROTEIN S11: P79013;

Filename	XCorr	DeltCN	ObsM+H+	CalcM+H+	SpR	SpScore	Ion%	#	Sequence	
100303-DM1159-p620-03.1592.1592.1	2.3919	0.1523	736.48	735.858	1	583.5	83.3%	1	K.DVGLGFK.T	1
100303-DM1159-p620-05.2825.2825.1	2.2639	0.417	1162.41	1163.328	1	152.2	66.7%	1	K.CPFVGQVSIR.G	1
100303-DM1159-p620-05.2829.2829.2	2.5953	0.2027	1163.21	1163.328	1	887.1	88.9%	1	K.CPFVGQVSIR.G	2
100303-DM1159-p620-05.2716.2716.2	2.799	0.308	1872.67	1873.083	1	337.8	43.8%	1	R.INEGDVVTVGQCRPLSK.T	2

U **RPL44,SPAC1687.06C,RPL28** 4 5 21.6% 134 14755 11.2 : 60S RIBOSOMAL PROTEIN L28/L44; NO APPARENT S. CEREVISIAE ORTHOLOG: O14069;P36585;

	Filename	XCorr	DeltCN	ObsM+H+	CalcM+H+	SpR	SpScore	Ion%	#	Sequence	
*	100303-DM1159-p620-03.1195.1195.1	2.0237	0.1205	940.31	940.995	1	454.3	71.4%	1	R.FSGLCNDK.A	1
*	100303-DM1159-p620-04.0978.0978.2	3.3034	0.2916	999.39	999.115	2	1073.1	83.3%	2	K.AVGVQANSPR.G	2
*	100303-DM1159-p620-04.0956.0956.1	2.0224	0.3011	999.44	999.115	2	194.7	66.7%	1	K.AVGVQANSPR.G	1
*	100303-DM1159-p620-05.2218.2218.2	2.9026	0.1665	1161.34	1158.343	48	435.2	60.0%	1	R.ASAILSSQRPK.K	2

U **RPL25A,SPBC106.18** 2 2 20.6% 141 15836 10.5 : 60S RIBOSOMAL PROTEIN L25: Q10330;

U **RPL25B,SPBC4F6.04,RPL23A-2** 2 2 20.6% 141 15768 10.5 : 60S RIBOSOMAL PROTEIN L25: O74391;

Filename	XCorr	DeltCN	ObsM+H+	CalcM+H+	SpR	SpScore	Ion%	#	Sequence	
100303-DM1159-p620-05.2492.2492.2	2.6511	0.2099	1228.09	1227.45	1	1639.0	85.0%	1	K.INTLIRPNGTK.K	2
100303-DM1159-p620-03.3491.3491.2	2.6093	0.3103	2033.01	2033.335	1	672.7	55.6%	1	T.KKAFVKLSADADALDVANR.I	2

U **RPL20-1,SPAC3A12.10,YL17B,RPL18A-2** 4 4 19.9% 176 20599 10.4 : 60S RIBOSOMAL PROTEIN L20A: P05732;

U **RPL20-2,SPAC26A3.04** 4 4 19.9% 176 20599 10.4 : 60S RIBOSOMAL PROTEIN L20: P05732;

Filename	XCorr	DeltCN	ObsM+H+	CalcM+H+	SpR	SpScore	Ion%	#	Sequence	
100303-DM1159-p620-05.2092.2092.2	2.5059	0.2741	1260.96	1261.464	4	474.5	70.0%	1	R.KVPTEHEPVPK.L	2
100303-DM1159-p620-04.2347.2347.2	2.6481	0.3363	1454.81	1455.687	1	891.6	57.7%	1	R.VGAVEAMYADMAAR.H	2

	100303-DM1159-p620-04.1287.1287.1	1.9667	0.269	801.51	801.962	1	386.2	62.5%	1	R.TGVVGLAGK.K	1
	100303-DM1159-p620-05.2257.2257.2	2.5185	0.2884	930.58	930.136	11	570.2	72.2%	1	R.TGVVGLAGKK.V	2

U **CKA1,SPAC23C11.11,ORB5** 6 11 19.3% 332 39527 7.8 : CASEIN KINASE II, ALPHA CHAIN; INVOLVED IN CELL GROWTH REGULATION: P40231;

	Filename	XCorr	DeltCN	ObsM+H+	CalcM+H+	SpR	SpScore	Ion%	#	Sequence	
*	100303-DM1159-p620-04.1294.1294.2	2.8949	0.3677	1344.55	1345.516	1	793.7	75.0%	1	R.VYAHVNEEMPR.E	2
*	100303-DM1159-p620-04.2718.2718.2	4.106	0.5079	1713.73	1713.885	1	1692.5	75.0%	3	K.YSEVFEGLNVLNNSK.C	2
*	100303-DM1159-p620-04.2355.2355.2	3.1789	0.3595	1458.62	1457.623	1	832.5	81.8%	2	R.TLYPTLSDYDIR.Y	2
*	100303-DM1159-p620-04.2774.2774.2	4.3107	0.4796	1483.55	1483.704	1	1602.9	79.2%	2	K.VLGTDELFAYVQK.Y	2
*	100303-DM1159-p620-04.2272.2272.2	3.9603	0.3181	1444.77	1444.585	1	1873.7	79.2%	2	R.SLANDEAIDLLNR.L	2
*	100303-DM1159-p620-02.2622.2622.2	3.0666	0.2371	1358.7	1357.506	1	602.4	72.7%	1	S.LANDEAIDLLNR.L	2

U **RPL11-1,SPAC26A3.07C** 3 5 19.0% 174 19891 10.1 : 60S RIBOSOMAL PROTEIN L11: Q10157;

U **RPL11-2,SPBC17G9.10** 3 5 19.0% 174 19891 10.1 : 60S RIBOSOMAL PROTEIN L11: Q10157;

Filename	XCorr	DeltCN	ObsM+H+	CalcM+H+	SpR	SpScore	Ion%	#	Sequence	
100303-DM1159-p620-04.1926.1926.2	4.2135	0.4319	1532.67	1533.765	1	1449.2	76.9%	2	K.VLEQLSGQTPVFSK.A	2
100303-DM1159-p620-05.2100.2100.2	2.5489	0.2557	956.34	956.099	1	784.4	85.7%	1	K.IACHVTVR.G	2
100303-DM1159-p620-04.2710.2710.2	3.9279	0.4155	1352.7	1351.502	1	1561.6	90.0%	2	K.INAEDTINWFK.Q	2

U **RPL28-1,SPBC776.11** 3 5 18.9% 148 16685 11.1 : 60S RIBOSOMAL PROTEIN LL28B 27A: P57728;

	Filename	XCorr	DeltCN	ObsM+H+	CalcM+H+	SpR	SpScore	Ion%	#	Sequence	
	100303-DM1159-p620-04.2522.2522.2	5.2146	0.537	1789.76	1790.026	1	1410.1	78.1%	3	K.NTEVAPVINVLQSGYGK.V	22
*	100303-DM1159-p620-04.1815.1815.1	2.2422	0.2946	1252.7	1253.484	3	234.1	60.0%	1	R.LPDTPVIIQTR.Y	1
*	100303-DM1159-p620-04.1810.1810.2	2.8226	0.2991	1252.75	1253.484	1	562.0	70.0%	1	R.LPDTPVIIQTR.Y	2

Similarities: RPL28-2,SPCC5E4.07(1:2)

U **RPS5-2,SPAC328.10C** 3 3 18.2% 203 22357 9.8 : 40S RIBOSOMAL PROTEIN S5: Q9P3T6;

Filename	XCorr	DeltCN	ObsM+H+	CalcM+H+	SpR	SpScore	Ion%	#	Sequence	
100303-DM1159-p620-05.1822.1822.2	2.604	0.3216	917.03	917.056	1	817.9	81.2%	1	T.RIGSAGTVR.R	22
100303-DM1159-p620-05.3401.3401.2	2.596	0.27	1341.04	1340.609	1	1529.2	75.0%	1	R.VNQALALITIGAR.E	22
100303-DM1159-p620-05.3716.3716.2	2.5254	0.1856	1648.35	1648.817	1	774.7	53.6%	1	K.SISECLAEEIINAAK.G	22

Similarities: RPS5,SPAC8C9.08(3:0)

U **RPS14-1,SPAC3H5.05C** 3 3 18.0% 139 14699 10.7 : 40S RIBOSOMAL PROTEIN S14: O14150;

U **RPS14-2,SPBC18H10.13** 3 3 18.0% 139 14699 10.7 : 40S RIBOSOMAL PROTEIN S14: O14150;

Filename	XCorr	DeltCN	ObsM+H+	CalcM+H+	SpR	SpScore	Ion%	#	Sequence	
100303-DM1159-p620-05.2216.2216.2	3.4378	0.4719	1038.88	1039.18	1	1148.5	85.0%	1	K.TPGPGAQAALR.A	2
100303-DM1159-p620-03.1403.1403.2	3.6646	0.383	1445.46	1444.582	1	987.5	83.3%	1	R.IEDVTPIPTDSTR.R	2
100303-DM1159-p620-03.1352.1352.2	2.6272	0.2273	1600.18	1600.769	38	261.4	42.3%	1	R.IEDVTPIPTDSTRR.K	2

U **RPS30-1,SPAC19B12.04** 2 2 18.0% 61 6910 11.7 : 40S RIBOSOMAL PROTEIN S30: O14314;O42952;

U **RPS30-2,SPBC19G7.03C,YSPRS30** 2 2 18.0% 61 6910 11.7 : 40S RIBOSOMAL PROTEIN S30: O14314;O42952;

Filename	XCorr	DeltCN	ObsM+H+	CalcM+H+	SpR	SpScore	Ion%	#	Sequence	
100303-DM1159-p620-04.1847.1847.2	3.4822	0.3174	1165.59	1166.381	1	1214.1	85.0%	1	R.FVNVTNMVGGK.R	2
100303-DM1159-p620-04.1848.1848.1	2.6197	0.2243	1166.58	1166.381	1	654.1	70.0%	1	R.FVNVTNMVGGK.R	1

U **ECM2,SPCC550.02C,CWF5** 3 5 15.3% 354 39576 9.0 : PUTATIVE PRE-MRNA SPLICING FACTOR; 40S SNRNP- CONTAINING COMPLEX; RNA-BINDING PROTEIN; SIMILAR TO S. CEREVISIAE ECM2: O59800;Q9USC0;

	Filename	XCorr	DeltCN	ObsM+H+	CalcM+H+	SpR	SpScore	Ion%	#	Sequence	
*	100303-DM1159-p620-02.1348.1348.2	5.9439	0.57	1957.35	1955.045	1	1812.1	72.2%	1	R.LLSNGETAYDSQEASAAAR.N	2
*	100303-DM1159-p620-04.2319.2319.2	6.0471	0.5634	2061.51	2062.244	1	1476.1	67.5%	3	R.SSAEIAAAASPDGNVVIEGFR.L	2
*	100303-DM1159-p620-04.1754.1754.2	3.4496	0.337	1475.73	1475.688	1	560.0	69.2%	1	K.SPAVAIPIDPNQPR.Y	2

U **CKB1,SPAC1851.03** 4 8 15.2% 231 26677 6.3 : CASEIN KINASE II BETA CHAIN: P40232;Q9US19;

	Filename	XCorr	DeltCN	ObsM+H+	CalcM+H+	SpR	SpScore	Ion%	#	Sequence	
*	100303-DM1159-p620-03.3139.3139.2	5.762	0.5708	2023.59	2021.07	1	1888.4	76.7%	4	K.GNEFFCEVDEDFIQDR.F	2
*	100303-DM1159-p620-05.2985.2985.2	3.3997	0.3755	1169.71	1170.394	1	857.5	88.9%	1	R.YILTAQGLYK.M	2
*	100303-DM1159-p620-05.2988.2988.1	2.053	0.0844	1171.49	1170.394	2	458.4	55.6%	2	R.YILTAQGLYK.M	1
*	100303-DM1159-p620-05.2364.2364.1	2.3097	0.309	1039.36	1040.164	1	570.7	81.2%	1	K.VHSYSATFK.K	1

U	**RPL8-1,SPAC1F7.13C,RPK5A,RPL2-1,SPAC21E1**	3	7	15.0%	253	27100	10.9	: 60S RIBOSOMAL PROTEIN L2A: P08093;P14067;P36593;Q9UU31;
U	**RPL8-3,SPBC839.04,RPK5-B,RPKD4**	3	7	15.0%	253	27100	10.9	: 60S RIBOSOMAL PROTEIN L8: P08093;P14067;P36593;Q9UU31;
U	**RPL8-2,SPBC2F12.07C,RPK37,RPK5B**	3	7	15.0%	253	27100	10.9	: 60S RIBOSOMAL PROTEIN L8 OR L2: P08093;P14067;P36593;Q9UU31;

	Filename	XCorr	DeltCN	ObsM+H+	CalcM+H+	SpR	SpScore	Ion%	#	Sequence	
	100303-DM1159-p620-05.3577.3577.2	4.8544	0.3558	2784.96	2783.166	1	540.2	42.3%	2	K.NAALTVGNVLPVGEMPEGTIISNVEEK.A	2
	100303-DM1159-p620-04.1458.1458.2	3.5792	0.4322	941.87	942.106	1	1636.1	90.0%	2	R.GVVGIVAGGGR.I	2
	100303-DM1159-p620-04.1462.1462.1	2.8464	0.3434	943.49	942.106	1	308.5	65.0%	3	R.GVVGIVAGGGR.I	1

U	**CWF2,SPAC3A12.11C**	3	4	14.4%	388	44281	5.7	: PRE-MRNA SPLICING COMPONENT; 40S SNRNP- CONTAINING COMPLEX; ZINC FINGER PROTEIN; ZF-CCCH TYPE; RRM DOMAIN; RNP-CONTAINING PROTEIN; SIMILAR TO S. CEREVISAE YDL209: CAC41387;P87126;Q9P8I7;

	Filename	XCorr	DeltCN	ObsM+H+	CalcM+H+	SpR	SpScore	Ion%	#	Sequence	
*	100303-DM1159-p620-05.3689.3689.2	2.7782	0.2961	2485.64	2484.722	2	237.6	28.6%	1	R.QNYTLYVGGITPTDDIEEIVSR.H	2
*	100303-DM1159-p620-05.2498.2498.2	2.8204	0.1251	2205.99	2205.216	2	498.9	39.5%	1	L.AANEFPNKSQSEEGSNDDHK.S	2
*	100303-DM1159-p620-04.2546.2546.2	4.063	0.3502	1562.48	1562.806	1	1442.6	80.8%	2	K.FVNSQILSDLQVAK.Q	2

U	**RPL4,SPBC1711.06**	3	7	14.3%	363	39910	10.7	: 60S RIBOSOMAL PROTEIN L2: O94253;P35679;Q9P784;

	Filename	XCorr	DeltCN	ObsM+H+	CalcM+H+	SpR	SpScore	Ion%	#	Sequence	
*2	100303-DM1159-p620-04.3306.3306.2	2.8637	0.3923	1697.48	1697.926	1	945.7	60.0%	1	K.DGSVSSETLALPFVFK.A	22
	100303-DM1159-p620-03.3651.3651.2	4.5281	0.4672	2255.24	2256.558	1	1096.1	68.4%	3	R.IEEIPEVPLVVDDAVQSFQK.T	22
	100303-DM1159-p620-04.2866.2866.2	4.8238	0.449	1867.07	1867.068	1	1441.7	80.0%	3	K.NYFLPENIISNADVTR.L	22

Similarities: RPL4-2,SPBP8B7.03C(3:0)

U	**RPL4-2,SPBP8B7.03C**	3	7	14.3%	363	39767	10.8	: 60S RIBOSOMAL PROTEIN L2: O94253;P35679;

	Filename	XCorr	DeltCN	ObsM+H+	CalcM+H+	SpR	SpScore	Ion%	#	Sequence	
*2	100303-DM1159-p620-04.3306.3306.2	2.8637	0.3923	1697.48	1697.926	1	945.7	60.0%	1	K.DGSVSSETIALPFVFK.A	22
	100303-DM1159-p620-03.3651.3651.2	4.5281	0.4672	2255.24	2256.558	1	1096.1	68.4%	3	R.IEEIPEVPLVVDDAVQSFQK.T	22
	100303-DM1159-p620-04.2866.2866.2	4.8238	0.449	1867.07	1867.068	1	1441.7	80.0%	3	K.NYFLPENIISNADVTR.L	22

Similarities: RPL4,SPBC1711.06(3:0)

U	**RPS23,SPAC23C11.02C**	2	3	13.3%	143	15655	10.3	: 40S RIBOSOMAL PROTEIN S23: P79057;
U	**RPS23-2,SPBP4H10.13**	2	3	13.3%	143	15655	10.3	: 40S RIBOSOMAL PROTEIN S23: P79057;

Filename	XCorr	DeltCN	ObsM+H+	CalcM+H+	SpR	SpScore	Ion%	#	Sequence	
100303-DM1159-p620-04.1090.1090.1	1.8556	0.166	769.56	769.879	10	115.1	57.1%	1	K.PAGLNAAR.K	1
100303-DM1159-p620-05.1992.1992.1	2.3423	0.3301	1063.32	1062.127	1	370.1	55.0%	2	K.SSPFGGSSHAK.G	1

U	**SPAC56F8.05C**	2	2	13.2%	295	32724	5.8	: HYPOTHETICAL PROTEIN; SIMILAR TO S. CEREVISIAE YPR148C AND YIL041W; POSSIBLY FUNGAL SPECIFIC: Q10253;

	Filename	XCorr	DeltCN	ObsM+H+	CalcM+H+	SpR	SpScore	Ion%	#	Sequence	
*	100303-DM1159-p620-04.3592.3592.2	4.1653	0.3843	2464.25	2463.698	1	689.2	57.5%	1	R.VEELSLPEDYVELEQQVDSLK.E	2
*	100303-DM1159-p620-05.3330.3330.2	2.9675	0.3559	1852.77	1853.126	1	291.1	44.1%	1	R.NLGNLTPLEQTPLASVGK.N	2

U	**ISY1,SPBC32F12.05C**	2	2	12.9%	217	25633	6.3	: PUTATIVE PRE-MRNA SPLICING FACTOR; SIMILAR TO S. CEREVISIAE ISY1: O74370;

	Filename	XCorr	DeltCN	ObsM+H+	CalcM+H+	SpR	SpScore	Ion%	#	Sequence	
*	100303-DM1159-p620-03.1724.1724.2	2.5833	0.081	1418.74	1419.621	2	562.5	68.2%	1	R.IQSATLPEYQIR.D	2
*	100303-DM1159-p620-03.2295.2295.2	5.7726	0.5634	1944.57	1945.003	1	1806.7	73.3%	1	K.LFEDEGEQISDIDDYR.Y	2

U	**GPD1,SPBC32F12.11,TDH1**	4	6	11.3%	336	35870	6.7	: GLYCERALDEHYDE 3-PHOSPHATE DEHYDROGENASE: P78958;

	Filename	XCorr	DeltCN	ObsM+H+	CalcM+H+	SpR	SpScore	Ion%	#	Sequence	
	100303-DM1159-p620-05.3157.3157.2	3.7606	0.4794	1469.53	1469.718	1	1228.7	80.8%	3	R.VPTPDVSVVDLTVK.L	22
	100303-DM1159-p620-04.1276.1276.2	2.5401	0.1713	1292.26	1292.475	3	401.7	70.0%	1	K.LAKPTNYEDIK.A	22
*	100303-DM1159-p620-04.1871.1871.1	2.361	0.3931	1150.62	1150.36	1	558.9	60.0%	1	R.VVDLVAYTAAK.D	1
*	100303-DM1159-p620-03.1811.1811.2	3.0274	0.354	1379.28	1379.553	1	1501.9	79.2%	1	R.VVDLVAYTAAKDN.-	2

Similarities: GPD3,SPBC354.12(2:2)

U	**HSP70,SPCC1739.13**	5	8	10.4%	647	70233	5.2	: HEAT SHOCK PROTEIN 70 FAMILY: O59855;
U	**SPAC13G7.02C**	5	8	10.4%	644	70143	5.2	: HEAT SHOCK PROTEIN 70 FAMILY: Q10265;

Filename	XCorr	DeltCN	ObsM+H+	CalcM+H+	SpR	SpScore	Ion%	#	Sequence	
100303-DM1159-p620-03.1742.1742.2	2.7631	0.3877	1487.68	1488.594	1	1080.2	79.2%	1	R.TTPSYVAFTDTER.L	2

	Filename	XCorr	DeltCN	ObsM+H+	CalcM+H+	SpR	SpScore	Ion%	#	Sequence	
	100303-DM1159-p620-05.3454.3454.2	3.4512	0.3514	1212.85	1212.435	1	1541.8	86.4%	2	K.DAGLIAGLNVLR.I	2
	100303-DM1159-p620-04.2426.2426.2	5.157	0.547	1691.38	1688.921	1	1323.5	73.3%	3	R.IINEPTAAAIAYGLDR.S	2
	100303-DM1159-p620-03.1295.1295.2	3.7905	0.427	1649.87	1649.671	1	1004.2	66.7%	1	K.ATAGDTHLGGEDFDSR.L	2
	100303-DM1159-p620-03.2638.2638.2	2.5516	0.2174	1300.42	1300.42	1	1316.6	83.3%	1	R.FEELCADLFR.K	2

U **RPS2,SPCC576.08C** 2 2 10.3% 253 27637 10.1 : 40S RIBOSOMAL PROTEIN S2: O74892;

	Filename	XCorr	DeltCN	ObsM+H+	CalcM+H+	SpR	SpScore	Ion%	#	Sequence	
*	100303-DM1159-p620-04.1307.1307.1	2.5144	0.4379	983.38	984.184	1	697.8	70.0%	1	R.GAGLVAAPVTK.R	1
*	100303-DM1159-p620-04.2498.2498.2	4.3527	0.4098	1801.65	1801.96	1	2440.2	89.3%	1	R.FLQLAGIEDCYTQSR.G	2

U **RPS25-1,SPBC3D6.15** 2 2 10.2% 88 9820 10.4 : 40S RIBOSOMAL PROTEIN S25: O74172;Q9UU26;
U **RPS25-2,SPAC694.05C** 2 2 10.1% 89 9959 10.4 : 40S RIBOSOMAL PROTEIN S25 (S31): P79009;Q9P7T5;

	Filename	XCorr	DeltCN	ObsM+H+	CalcM+H+	SpR	SpScore	Ion%	#	Sequence	
	100303-DM1159-p620-04.1211.1211.1	1.9575	0.2115	1016.4	1017.13	1	742.2	68.8%	1	K.AQHATVFDK.S	1
	100303-DM1159-p620-04.1208.1208.2	2.7928	0.257	1017.11	1017.13	1	885.3	81.2%	1	K.AQHATVFDK.S	2

U **NOC1,SPAC3C7.12,TIP1** 4 5 10.0% 461 52607 4.6 : SIMILAR TO EUKARYOTIC INTERMEDIATE FILAMENT PROTEINS; INVOLVED IN ORGANIZATION OF CYTOSKELETON: P79065;

	Filename	XCorr	DeltCN	ObsM+H+	CalcM+H+	SpR	SpScore	Ion%	#	Sequence	
*	100303-DM1159-p620-03.1800.1800.1	1.9476	0.1124	1050.37	1051.185	7	336.9	78.6%	1	R.IEDLLYER.Q	1
*	100303-DM1159-p620-03.1408.1408.2	3.0057	0.3284	1440.35	1439.485	1	926.2	68.2%	1	K.TTIECLEESSNR.A	2
*	100303-DM1159-p620-03.2559.2559.2	3.3822	0.242	1579.95	1580.692	1	1503.2	79.2%	1	K.SEDDLLFSLQQER.D	2
*	100303-DM1159-p620-03.2314.2314.2	4.8733	0.2961	1591.23	1591.761	1	2235.1	87.5%	2	R.DYALNQVEILQER.V	2

U **SPBC4C3.07** 2 2 9.9% 302 33251 6.4 : MOV34 DOMAIN PROTEIN; PUTATIVE COP9/SIGNALOSOME COMPLEX SUBUNIT 6; NO APPARENT S. CEREVISIAE ORTHOLOG: O43060;

	Filename	XCorr	DeltCN	ObsM+H+	CalcM+H+	SpR	SpScore	Ion%	#	Sequence	
*	100303-DM1159-p620-05.2856.2856.1	1.8561	0.2586	929.63	930.136	1	194.8	62.5%	1	R.VIGTLLGTR.S	1
*	100303-DM1159-p620-03.2338.2338.2	3.8087	0.4699	2147.26	2148.337	1	1361.9	55.0%	1	R.VSDYVQNVIDGSSPANVAVGR.Y	2

U **RPL17,SPBC2F12.04** 2 2 9.6% 187 20814 10.4 : 60S RIBOSOMAL PROTEIN L17: O14339;O59794;
U **RPL17-2,SPCC364.03** 2 2 9.6% 187 20827 10.3 : 60S RIBOSOMAL PROTEIN L17: ;N0ACC;

	Filename	XCorr	DeltCN	ObsM+H+	CalcM+H+	SpR	SpScore	Ion%	#	Sequence	
	100303-DM1159-p620-04.2696.2696.1	2.7494	0.193	1068.46	1067.273	1	766.1	75.0%	1	K.AFIFLDNVK.E	1
	100303-DM1159-p620-05.1828.1828.2	3.0619	0.3154	964.25	964.112	1	939.8	93.8%	1	K.HVQVNAAPK.Q	2

U **RPL37A-1,SPBC800.04C,RPL43-1** 2 4 9.6% 94 10422 10.8 : 60S RIBOSOMAL PROTEIN L37A/L43A: Q9HGL8;

	Filename	XCorr	DeltCN	ObsM+H+	CalcM+H+	SpR	SpScore	Ion%	#	Sequence	
	100303-DM1159-p620-05.1858.1858.1	1.9193	0.1109	1124.77	1125.273	1	199.6	62.5%	1	R.KIEVQQHSR.Y	11
	100303-DM1159-p620-05.1824.1824.2	2.8993	0.3235	1124.87	1125.273	1	1004.5	87.5%	3	R.KIEVQQHSR.Y	22

Similarities: RPL37A-2,SPBC83.02C,RPL43-2(2:0)

U **SPBC2D10.11C** 3 3 9.5% 379 43371 4.6 : PUTATIVE NUCLEOSOME ASSEMBLY PROTEIN: P78920;

	Filename	XCorr	DeltCN	ObsM+H+	CalcM+H+	SpR	SpScore	Ion%	#	Sequence	
*	100303-DM1159-p620-05.2734.2734.1	1.9152	0.1745	1183.59	1184.294	1	332.3	50.0%	1	K.SAHDEVSGLLR.N	1
*	100303-DM1159-p620-03.1820.1820.2	2.501	0.3299	1592.43	1592.749	1	551.5	60.7%	1	K.SSGYIESLAPAVQNR.I	2
*	100303-DM1159-p620-03.1784.1784.2	2.596	0.2772	1318.16	1317.364	1	1242.1	83.3%	1	K.DCDAIQYEFR.Q	2

U **DSK1,SPBC530.14C** 3 5 7.9% 544 61056 8.1 : SERINE/THREONINE PROTEIN KINASE; PRE-MRNA SPLICING FACTOR SPECIFIC KINASE: O59749;P36616;

	Filename	XCorr	DeltCN	ObsM+H+	CalcM+H+	SpR	SpScore	Ion%	#	Sequence	
*	100303-DM1159-p620-03.1768.1768.2	3.309	0.3218	1277.11	1277.389	1	1418.2	90.0%	1	K.IADLGNACWTR.K	2
*	100303-DM1159-p620-05.3074.3074.2	3.8683	0.377	1497.23	1497.668	1	1988.7	83.3%	2	R.TNAGYMSNSPWLR.E	2
*	100303-DM1159-p620-03.2204.2204.2	5.4566	0.4837	2003.85	2004.161	1	1182.3	72.2%	2	K.IETTGATGEDVPGWATEIR.-	2

U **ENO1,SPBC1815.01** 2 2 7.5% 439 47436 6.7 : ENOLASE: P40370;Q12703;Q9Y7J7;

	Filename	XCorr	DeltCN	ObsM+H+	CalcM+H+	SpR	SpScore	Ion%	#	Sequence	
*	100303-DM1159-p620-04.1947.1947.2	4.3385	0.5352	1588.08	1587.773	1	1480.5	75.0%	1	K.VNQIGSVTESLNAVR.M	2
*	100303-DM1159-p620-04.1483.1483.2	4.4808	0.4264	1817.61	1817.95	1	1947.0	73.5%	1	R.IEEELGSEGVYAGAHAGK.Y	2

U **GPD3,SPBC354.12** 2 4 7.5% 335 35675 7.9 : GLYCERALDEHYDE 3-PHOSPHATE DEHYDROGENASE: O43026;

	Filename	XCorr	DeltCN	ObsM+H+	CalcM+H+	SpR	SpScore	Ion%	#	Sequence	
	100303-DM1159-p620-05.3157.3157.2	3.7606	0.4794	1469.53	1469.718	1	1228.7	80.8%	3	R.VPTPDVSVVDLTVK.L	22
	100303-DM1159-p620-04.1276.1276.2	2.5401	0.1713	1292.26	1292.475	3	401.7	70.0%	1	K.LAKPTNYEDIK.A	22

Similarities: GPD1,SPBC32F12.11,TDH1(2:0)

U	**CDC5,SPAC644.12**	4	5	7.1%	757	86844	7.9	: CELL DIVISION CONTROL PROTEIN; TRANSCRIPTIONAL REGULATOR; MYB FAMILY DNA-BINDING PROTEIN; ESSENTIAL; REQUIRED FOR PRE-MRNA SPLICING; REQUIRED FOR G2/M PHASE PROGRESSION AND NUCLEAR DIVISION; FUNCTIONAL HOMOLOG OF S. CEREVISIAE CEF1: P39964;

	Filename	XCorr	DeltCN	ObsM+H+	CalcM+H+	SpR	SpScore	Ion%	#	Sequence	
*	100303-DM1159-p620-04.1623.1623.1	1.9607	0.082	789.44	787.978	42	102.8	66.7%	2	R.ISSLLVR.K	1
*	100303-DM1159-p620-04.3276.3276.2	3.9921	0.3803	2282.9	2283.364	1	977.7	55.0%	1	R.FADETDVEATVGVLEEDATDR.E	2
*	100303-DM1159-p620-05.2565.2565.2	2.9665	0.1973	1384.6	1383.588	2	406.8	59.1%	1	R.DLIRPSVTQPEK.W	2
*	100303-DM1159-p620-03.1868.1868.2	4.103	0.2534	1543.62	1543.714	1	1023.2	76.9%	1	R.SLENEDPTANVLLK.E	2

U	**SPBC2G5.02C**	2	2	7.1%	254	29061	5.1	: PUTATIVE CASEIN KINASE II BETA CHAIN: O94281;

	Filename	XCorr	DeltCN	ObsM+H+	CalcM+H+	SpR	SpScore	Ion%	#	Sequence	
*	100303-DM1159-p620-04.1191.1191.2	2.9486	0.3268	1376.28	1376.509	1	585.5	72.7%	1	K.LNHDPTVTYSTK.D	2
2	100303-DM1159-p620-03.0530.0530.1	1.8425	0.0887	662.39	662.761	3	383.3	80.0%	1	K.VSELSK.T	1

U	**RPL7A,SPBC29A3.04**	2	2	6.9%	259	28607	10.4	: 60S RIBOSOMAL PROTEIN L7A (L8): O13672;P17937;

	Filename	XCorr	DeltCN	ObsM+H+	CalcM+H+	SpR	SpScore	Ion%	#	Sequence	
*	100303-DM1159-p620-03.1686.1686.2	2.5783	0.2789	1190.07	1190.341	1	777.4	75.0%	1	R.SFGIGQDIQPK.R	2
*	100303-DM1159-p620-03.1138.1138.1	2.2101	0.2416	744.34	744.91	1	616.5	83.3%	1	K.TQALLAK.R	1

U	**BIP,SPAC22A12.15C**	2	2	4.7%	663	73227	5.0	: 78 KD GLUCOSE REGULATED PROTEIN HOMOLOG ; HSP70 FAMILY: O13906;P36604;

Filename	XCorr	DeltCN	ObsM+H+	CalcM+H+	SpR	SpScore	Ion%	#	Sequence	
100303-DM1159-p620-03.1896.1896.2	2.9012	0.41	1528.7	1528.659	1	692.4	66.7%	1	R.ITPSYVAFTEDER.L	22
100303-DM1159-p620-04.3334.3334.2	5.1456	0.5639	2122.68	2120.28	1	1767.9	76.5%	1	R.IEIESFFNGQDFSETLSR.A	22

Similarities: contaminant_GR78_SCHPO(2:0)

U	**SFC6,SPBC21H7.05**	2	2	3.3%	582	66203	6.5	: RNA POLYMERASE III TRANSCRIPTION FACTOR TFIIIC SUBUNIT; WD REPEAT PROTEIN: O60174;

	Filename	XCorr	DeltCN	ObsM+H+	CalcM+H+	SpR	SpScore	Ion%	#	Sequence	
*	100303-DM1159-p620-03.4306.4306.1	1.8323	0.1651	842.49	839.921	75	70.5	58.3%	1	Q.QLLSYSE.N	1
*	100303-DM1159-p620-02.2271.2271.2	2.5118	0.0869	1518.57	1517.768	1	1302.6	72.7%	1	Y.IYPWQIQVNKVE.W	2

U	**CTF18,SPBC902.02C,CHL12**	2	3	3.2%	960	108650	7.0	: RFC-1 RELATED PROTEIN; REQUIRED FOR CHROMOSOME TRANSMISSION; SIMILAR TO S. CEREVISIAE CHL12 PROTEIN INVOLVED IN RECOMBINATION AND TELOMERE LENGTH REGULATION; POSSIBLY INVOLVED IN SISTER CHROMATID COHESION: Q9USQ1;

	Filename	XCorr	DeltCN	ObsM+H+	CalcM+H+	SpR	SpScore	Ion%	#	Sequence	
*	100303-DM1159-p620-03.2210.2210.2	2.6685	0.0928	1792.41	1793.885	19	517.8	46.4%	1	F.EDEENYLLELRNANA.L	2
*	100303-DM1159-p620-02.3620.3620.2	2.8396	0.0945	1838.68	1836.095	10	486.2	46.7%	2	S.TSSLIQSLFLQLDNKQ.I	2

U	**RPB2,SPAC23G3.01,SPAC521.06**	2	2	3.1%	1210	137849	6.8	: DNA-DIRECTED RNA POLYMERASE II 138 KD POLYPEPTIDE: Q02061;Q9P7B1;Q9P7T2;

	Filename	XCorr	DeltCN	ObsM+H+	CalcM+H+	SpR	SpScore	Ion%	#	Sequence	
*	100303-DM1159-p620-02.3634.3634.2	3.3464	0.091	1473.52	1473.67	8	626.6	58.3%	1	L.DISAEVSIVRDIR.E	2
*	100303-DM1159-p620-03.3008.3008.2	2.7196	0.2511	2427.86	2428.655	1	339.6	34.8%	1	K.VSALSGFEGDATPFTDVTVEAVSK.L	2

U	**FAS1,SPAC926.09C**	3	6	2.5%	2073	230558	6.4	: FATTY ACID SYNTHASE, SUBUNIT BETA: P78799;Q9UUG0;

	Filename	XCorr	DeltCN	ObsM+H+	CalcM+H+	SpR	SpScore	Ion%	#	Sequence	
*	100303-DM1159-p620-03.2995.2995.2	5.572	0.5155	2179.85	2179.391	1	2322.9	72.2%	3	K.TTLIENFEDLNDPYPVAAR.F	2
*	100303-DM1159-p620-03.3167.3167.2	5.29	0.3114	2046.87	2047.186	1	2549.7	75.0%	2	K.DSLWQSEDLAAVVGEDVGR.V	2
*	100303-DM1159-p620-03.1406.1406.2	2.6768	0.2587	1524.13	1524.673	1	706.1	70.8%	1	R.FVETYAAQNVPER.V	2

U	**LSD1,SPAC4A8.11C,FAS2**	3	3	1.7%	1842	202168	6.2	: FATTY ACID SYNTHASE, SUBUNIT ALPHA: O14163;P78973;Q10289;Q96WT6;Q96WT7;Q96WT8;

	Filename	XCorr	DeltCN	ObsM+H+	CalcM+H+	SpR	SpScore	Ion%	#	Sequence	
*	100303-DM1159-p620-04.1214.1214.2	2.5493	0.0986	1152.89	1152.293	2	696.6	61.5%	1	E.TAAAAAPAATPAPA.P	2
*	100303-DM1159-p620-03.0290.0290.1	1.8135	0.1849	884.5	887.067	39	104.8	64.3%	1	P.GRLESVLL.L	1
*	100303-DM1159-p620-04.2176.2176.1	2.0766	0.1542	996.4	996.235	1	501.2	68.8%	1	K.TPLPIVDLK.F	1

U	**SPP42,SPAC4F8.12C,CWF6**	2	2	1.3%	2363	274554	8.9	: U5 SNRNA-ASSOCIATED SPLICING FACTOR; PRE-MRNA SPLICING FACTOR; SIMILAR TO S. CEREVISIAE PRP8: O14187;

	Filename	XCorr	DeltCN	ObsM+H+	CalcM+H+	SpR	SpScore	Ion%	#	Sequence	
*	100303-DM1159-p620-03.3496.3496.2	3.5686	0.3924	2364.86	2363.529	1	614.2	52.8%	1	R.MDLDEEDDAPVMDWFYENK.A	2
*	100303-DM1159-p620-03.3140.3140.2	3.3089	0.3792	1493.06	1492.582	1	1219.2	77.3%	1	R.LTLEDLEDSWDR.G	2

	Proteins	Peptide IDs	Copies
Unfiltered	4332	11890	18790
Redundant	89	283	487
Nonredundant	73	262	453

mts3-1 dma1 deletion dnt1 deletion
+ pREP41-NTAP-dma1-5xGly-Ub(7K-->7R)

DTASelect v1.9
SEQUEST 2.7 in SQT

format.sequest.params modifications:

*	S	80.0
#	T	80.0
@	Y	80.0
Static	C	57.0

true	Use criteria
1.8	Minimum +1 XCorr
2.5	Minimum +2 XCorr
3.5	Minimum +3 XCorr
4.5	Minimum +4 XCorr
0.01	Minimum DeltCN
1	Minimum charge state
9	Maximum charge state
0.0	Minimum ion proportion
1000	Maximum Sp rank
-1.0	Minimum Sp score
Require	Modified peptide inclusion
Any	Tryptic status requirement
true	Multiple, ambiguous IDs allowed
Ignore	Peptide validation handling
XCorr	Purge duplicate peptides by protein
false	Include only loci with unique peptide
false	Remove subset proteins
Ignore	Locus validation handling
0	Minimum modified peptides per locus
10	Minimum redundancy for low coverage loci
2	Minimum peptides per locus

Locus Key:

Validation Status	Locus	Sequence Count	Spectrum Count	Sequence Coverage	Length	MolWt	pI	Descriptive Name

Similarity Key:

Locus	# of identical peptides	# of differing peptides

U	SPBC215.13	38	43	47.4%	534	53362	6.5	|||glycoprotein |Schizosaccharomyces pombe|chr 2|||Manual

	Filename	XCorr	DeltCN	ObsM+H+	CalcM+H+	SpR	SpScore	Ion%	#	Sequence	
*	PARC_p629-03.12893.12893.3	3.501	0.0152	3871.43	3870.282	26	420.1	20.2%	1	F.SES*AT#PSETNSYSSPVSSY@SDPAT#SQLPS*S*T#S.F	3
*	PARC_p629-03.12369.12369.3	3.5384	0.0208	4036.88	4035.52	133	152.2	17.2%	1	E.S*AT#PSETNSY@SS*PVSS*YSDPAT#SQLPSS*TSFFS.P	3
*	PARC_p629-02.20099.20099.3	3.5792	0.0117	3837.38	3836.435	16	633.8	21.7%	1	S.YSS*PVSSYSDPATSQLPSS*TSFFSPT#SS*EY@T#.P	3
*	PARC_p629-03.14506.14506.2	2.6152	0.0544	2348.51	2347.329	3	204.2	31.0%	1	I.LPSSTSVEVSISS*SSLS*SSDPL.T	2
*	PARC_p629-02.12281.12281.2	2.6995	0.0359	1901.31	1901.688	10	244.1	37.5%	1	P.SSTSVEVSISS*S*S*LSSS.D	2
*	PARC_p629-03.11661.11661.2	2.6339	0.0102	1901.35	1901.688	15	228.0	37.5%	1	P.SSTSVEVSISS*S*SLS*SS.D	2
*	PARC_p629-03.12409.12409.3	3.7185	0.0174	3882.53	3882.538	223	159.1	17.6%	1	S.STSVEVSISSSSLSSSDPLTSS*T#FS*SLS*SS*TSSSQ.P	3
*	PARC_p629-02.14235.14235.3	3.8257	0.0671	3870.65	3870.527	120	250.0	17.9%	1	S.SSYLSSS*SVVSS*SS*SPSSSSSSTLTSSSLST#SS*IPS.T	3
*	PARC_p629-02.11836.11836.3	4.0995	0.0138	3878.51	3879.286	95	755.6	24.3%	1	S.T#LTSSSLS*T#SSIPSTSSSSSSTS*S*S*LSSSSSSST#A.S	3
*	PARC_p629-04.13837.13837.3	3.5207	0.0259	4130.36	4131.755	62	366.1	15.0%	1	S.TLTSSS*LSTSSIPS*TSSSSSSTSSS*LSSSSSSST#ASSSSSS.S	3
*2	PARC_p629-04.08295.08295.2	2.5708	0.0117	2233.93	2233.052	6	214.3	33.3%	1	L.TSSSLSTSSIPSTSSSSSST#S*S.S	2
*2	PARC_p629-04.08295.08295.2	2.5708	0.0117	2233.93	2233.052	6	214.3	33.3%	1	L.TSSSLSTSSIPSTSSSSSST#SS*.S	2
*	PARC_p629-02.14496.14496.3	3.9751	0.017	3986.45	3985.28	1	265.0	20.1%	1	S.ST#S*SSLSSSSSSST#ASSSSSSSSIISSSSSSSS*S*PT#S*.T	3
*	PARC_p629-05.11069.11069.3	3.5048	0.0185	3838.43	3839.545	238	383.3	16.4%	1	S.SLSS*SSSSST#ASSSSSS*SSIISSSSSSSSSPTSTSSTIS.S	3
*	PARC_p629-02.15311.15311.3	4.3992	0.0113	3838.46	3839.545	254	437.7	17.8%	2	S.SLSS*SSSSSTASSSSS*S*SSIISSSSSSSSSPTSTSSTIS.S	3
*	PARC_p629-03.15255.15255.3	4.4743	0.0218	3843.29	3842.466	65	479.9	18.2%	1	S.SLSSSSS*SSTASSSSSS*SS*IISSSSSSSSSPTST#SSTI.S	3
*	PARC_p629-01.17820.17820.2	2.502	0.0192	2074.95	2074.934	71	155.1	31.0%	1	S.SSSSSSTASS*SSSSSSIISSSS.S	2
*	PARC_p629-01.18217.18217.2	3.1142	0.0225	2076.01	2074.934	68	147.8	31.0%	4	S.SSSSSTASSS*SSSSSIISSSSS.S	2
*	PARC_p629-04.16978.16978.3	3.6532	0.0182	4077.32	4077.62	111	316.4	17.5%	1	S.S*SSSTASSSSS*SSSIISSSSSSSS*S*SPTSTSSTISSSSSSSSS.S	3
*	PARC_p629-01.11219.11219.2	2.5624	0.05	1621.61	1622.227	10	252.9	42.3%	1	S.S*SS*TASSSSSSSS*S*I.I	2
*	PARC_p629-02.18299.18299.3	4.0503	0.0204	4080.71	4080.542	178	141.8	16.0%	1	S.S*STASSSSSSSSIISS*SSSSSSSPT#ST#SSTISSSSSSSSSS*.P	3
*	PARC_p629-02.15693.15693.2	2.7197	0.0407	2460.01	2460.971	88	204.4	28.6%	1	A.SSSSSSSS*IISSSSSS*S*SSPT#S*.T	2
*	PARC_p629-04.15517.15517.3	3.5357	0.0167	4153.19	4151.949	186	282.0	14.9%	1	S.SSIISS*SSSSSSSPTSTSSTISSSSSSSSSPTSTSST#ISSSSS.S	3
	PARC_p629-01.19336.19336.1	1.8207	0.0371	1371.97	1371.148	42	229.3	37.5%	2	T.SSTISS*SSS*SSSS.P	1
*	PARC_p629-	3.1925	0.0105	2681.29	2680.13	11	162.7	29.5%	1	S.ST#ISSSSS*S*S*SSPTST#S*STISSS.S	2

	03.11984.11984.2										
*	PARC_p629-04.11388.11388.2	2.8436	0.0128	1781.71	1782.481	4	412.8	50.0%	1	T.SSTISSSS*S*SSSSFS*S.T	2
*	PARC_p629-02.13659.13659.2	2.5334	0.0159	1783.27	1782.481	134	187.7	36.7%	1	T.SSTISS*S*SSSSSSFSS*.T	2
*	PARC_p629-01.16896.16896.2	2.8454	0.038	1872.69	1872.481	3	324.2	43.3%	1	T.SSTISS*SSSSSSS*FS*S*.T	2
*	PARC_p629-05.10192.10192.2	2.9612	0.0116	2346.93	2346.143	6	341.8	31.8%	1	S.SSSSSSSS*FSST#LSSSSMSSSSS.F	2
*	PARC_p629-05.08645.08645.3	3.621	0.0214	3876.83	3878.275	52	527.4	20.5%	1	S.SSSS*SFSST#LS*SSSMSSSSSFS*S*SPT#SSSST#ISS.S	3
*	PARC_p629-02.11552.11552.3	4.3702	0.0128	3879.5	3879.548	146	448.5	18.8%	1	S.SS*SFSSTLSS*S*S*MSSSSSFSSSPTSSSSTISSSSSSP.S	3
*	PARC_p629-01.16940.16940.2	3.1249	0.015	1826.41	1826.595	4	544.9	50.0%	1	F.SSTLSS*SS*MSSSSSFS*.S	2
*	PARC_p629-01.17078.17078.2	2.6156	0.0973	1563.37	1562.439	2	410.9	53.8%	1	S.STLSSSSMSSSS*S*F.S	2
*	PARC_p629-01.19231.19231.3	3.5741	0.0188	2282.99	2283.13	33	799.2	31.0%	1	S.TLSSSS*MSSSSSFSSSPTSSS*S.T	3
*	PARC_p629-02.07406.07406.1	1.9621	0.1087	1047.72	1046.901	7	112.5	56.2%	1	S.PT#SSSST#IS.S	1
*	PARC_p629-02.07503.07503.1	1.988	0.0127	932.5	932.835	4	138.7	56.2%	1	S.SSST#ISSSS.S	1
*	PARC_p629-05.10865.10865.3	3.6228	0.0257	3252.38	3251.808	164	265.4	19.6%	1	S.TSSST#LTS*SS*SSSS*RPASSSSHSSSLSS*H.K	3
*	PARC_p629-02.13334.13334.2	3.0089	0.0147	1802.55	1802.48	3	296.9	46.4%	1	S.S*TLT#S*S*SSSSSRPAS.S	2

U **SPAC3A12.14** 2 2 34.0% 150 16906 4.3 |cam1||calmodulin |Schizosaccharomyces pombe|chr 1|||Manual

	Filename	XCorr	DeltCN	ObsM+H+	CalcM+H+	SpR	SpScore	Ion%	#	Sequence	
*	PARC_p629-03.18270.18270.3	3.5176	0.0118	2985.38	2984.058	2	613.5	26.0%	1	Q.DGNITS*NELGVVMRSLGQS*PTAAELQD.M	3
*	PARC_p629-02.17595.17595.3	5.2857	0.0288	4080.86	4081.406	2	446.2	21.5%	1	R.SLGQSPT#AAELQDMINEVDADGNGTIDFTEFLTMMAR.K	3

U **SPBC9B6.08** 3 3 30.1% 229 25863 4.7 |clc1||clathrin light chain|Schizosaccharomyces pombe|chr 2|||Manual

	Filename	XCorr	DeltCN	ObsM+H+	CalcM+H+	SpR	SpScore	Ion%	#	Sequence	
*	PARC_p629-02.07941.07941.2	3.1755	0.1567	2266.61	2265.453	72	184.0	38.2%	1	E.RDESSKKLRES*NIEKARK.A	2
*	PARC_p629-03.16430.16430.3	3.6485	0.0444	4092.83	4093.371	2	656.9	20.3%	1	K.AIDDFYENFNDKRDKVIAKS*RKEQEKLLEENES.K	3
*	PARC_p629-03.14650.14650.2	2.5239	0.0715	2598.03	2598.788	1	212.7	35.7%	1	E.ENESKS*TGTTSWERILKLIDLS.D	2

U **SPAC56F8.16** 7 8 29.3% 413 44798 9.0 |esc1||transcription factor |Schizosaccharomyces pombe|chr 1|||Manual

	Filename	XCorr	DeltCN	ObsM+H+	CalcM+H+	SpR	SpScore	Ion%	#	Sequence	
*	PARC_p629-03.15853.15853.2	2.9111	0.0135	2266.97	2267.047	1	438.7	50.0%	1	A.S*S*TPYS*PQQVPLTHNS*Y.P	2
*	PARC_p629-02.17107.17107.2	3.0152	0.0127	2267.75	2267.047	8	203.0	34.4%	1	A.S*S*TPY@SPQQVPLTHNS*Y.P	2
*	PARC_p629-02.10092.10092.1	2.3811	0.0134	1066.7	1065.988	19	116.6	45.0%	2	H.S*NSAAPASSSP.T	1
*	PARC_p629-03.16161.16161.3	3.6907	0.0204	3824.87	3824.684	203	325.3	17.9%	1	A.APASS*SPTSATLSTAAHPVHTNAAQVAGSSSS*Y@VY@S.V	3
*	PARC_p629-04.13838.13838.2	2.6073	0.0263	2495.55	2494.31	9	315.7	34.2%	1	H.S*AVPHRSS*QFQST#TLT#PSTT.D	2
*	PARC_p629-02.19746.19746.2	2.5028	0.0109	2558.87	2558.376	5	304.8	29.2%	1	S.TDVSS*SDSVSTSASSSNASNT#VSVT.S	23
*	PARC_p629-04.12669.12669.3	3.6519	0.11	2231.81	2231.996	1	711.0	36.1%	1	Q.QTSHSS*GASY@QNES*ANPPV.Q	3

U **SPAC11E3.15** 2 2 28.2% 117 13320 9.6 |rpl22|SPAP8A3.01|60S ribosomal protein L22|Schizosaccharomyces pombe|chr 1|||Manual

	Filename	XCorr	DeltCN	ObsM+H+	CalcM+H+	SpR	SpScore	Ion%	#	Sequence	
*	p629-05ms3.07239.07239.2	2.6853	0.0662	1366.17	1367.568	13	446.9	55.0%	1	-.MVKKNT#KVSNK.Y	2
*	p629-02ms3.10497.10497.2	2.7544	0.0324	2221.71	2222.422	6	126.7	28.6%	1	K.TGNLGS*SVVVSREGSSKIAVIA.H	2

U **SPAC17G8.10c** 6 42 28.1% 267 30597 6.9 |dma1||zinc finger protein|Schizosaccharomyces pombe|chr 1|||Manual

	Filename	XCorr	DeltCN	ObsM+H+	CalcM+H+	SpR	SpScore	Ion%	#	Sequence	
*	PARC_p629-02.12117.12117.3	4.1773	0.1552	4002.59	4003.241	1	617.6	20.6%	1	K.SVEGYLKEQELAAETDSEKDDDKISIRLT#NFVGPN.A	3
*	PARC_p629-02.18964.18964.3	6.0153	0.3214	3072.2	3073.213	1	1467.1	34.0%	20	S.VEGY@LKEQELAAETDSEKDDDKISIR.L	3
*	PARC_p629-03.11098.11098.2	3.5889	0.259	3073.89	3073.213	1	521.1	34.0%	2	S.VEGY@LKEQELAAETDSEKDDDKISIR.L	2
*	PARC_p629-02.12820.12820.3	5.4694	0.2106	3286.61	3287.477	1	1798.3	32.4%	7	S.VEGY@LKEQELAAETDSEKDDDKISIRLT.N	3
*	PARC_p629-04.11992.11992.3	4.2148	0.0372	3333.56	3332.299	1	888.9	32.0%	2	R.HAQIFYENNTWYIQDMGSS*S*GT#FLNH.V	3
*	PARC_p629-02.12152.12152.2	2.9521	0.0723	1801.49	1800.721	10	388.5	46.2%	10	S.PPS*KT#SKPY@PISNN.D	2

U **SPAC8C9.04** 11 11 28.0% 647 67036 5.6 |||sequence orphan|Schizosaccharomyces pombe|chr 1|||Manual

	Filename	XCorr	DeltCN	ObsM+H+	CalcM+H+	SpR	SpScore	Ion%	#	Sequence	
*	PARC_p629-02.17086.17086.2	2.5789	0.1153	2258.29	2259.35	176	191.7	26.2%	1	L.EPIASSALGTVEPPT#DNKPSAS.T	2
*	PARC_p629-04.13567.13567.2	2.5497	0.0115	2372.75	2374.064	1	180.1	37.5%	1	N.T#S*ITEPANSPSSS*SSSAS*TKS.T	2
*	PARC_p629-01.17557.17557.2	2.5491	0.0233	2059.49	2059.805	27	182.1	38.9%	1	N.SPSSS*S*S*SASTKSTATTQS.A	2
*	PARC_p629-02.09004.09004.3	3.5524	0.017	3281.39	3280.936	241	304.5	18.1%	1	N.TTHDQAAATTT#AAVASAS*TT#AT#AES*HAVAD.G	3
*	PARC_p629-03.16929.16929.3	3.7088	0.0345	4088.78	4089.996	120	374.0	17.1%	1	P.S*KQAS*APS*SPGTTSAAVPGGKKS*AIEAAAPIPTSADT#VE.S	3
*	PARC_p629-04.14449.14449.3	3.6192	0.0354	4207.25	4206.978	91	231.2	17.8%	1	S.ADT#VESKHAAGS*GSATT#IPS*PGS*ATTKPTPGSAT#TKPTP.V	3
*	PARC_p629-02.08614.08614.1	1.9289	0.0203	1524.69	1523.515	1	133.4	40.6%	1	H.AAGSGSATT#IPSPGSAT.T	1
*	PARC_p629-04.12661.12661.3	3.9583	0.0566	3661.4	3662.413	22	433.4	21.0%	1	S.ATTIPSPGS*AT#T#KPT#PGS*ATT#KPTPVSATEEH.A	3
*	PARC_p629-03.11596.11596.2	2.6346	0.0112	1768.25	1769.743	253	197.7	31.2%	1	P.S*PGSATTKPTPGSAT#TK.P	2
*	PARC_p629-02.12436.12436.2	2.558	0.0737	1769.53	1769.743	13	450.7	43.8%	1	P.S*PGSATTKPT#PGSATTK.P	2
*	PARC_p629-04.17423.17423.3	3.6703	0.045	3838.97	3837.802	6	614.4	18.2%	1	P.GSATTKPTPVS*AT#EEHAAGTTKPAPAAGATATAENET#A.D	3

U **SPAC13G6.11c** 11 96 25.7% 404 43407 6.9 |erg12||mevalonate kinase activity |Schizosaccharomyces pombe|chr 1|||Manual

	Filename	XCorr	DeltCN	ObsM+H+	CalcM+H+	SpR	SpScore	Ion%	#	Sequence	
*2	PARC_p629-	2.9034	0.0102	2786.57	2785.879	16	173.9	28.3%	1	H.AVVY@GATALAAAVSLRSY@CKLQTT#.N	2

	Filename	XCorr	DeltCN	ObsM+H+	CalcM+H+	SpR	SpScore	Ion%	#	Sequence	
	02.14467.14467.2										
*2	PARC_p629-02.14467.14467.2	2.9034	0.0102	2786.57	2785.879	16	173.9	28.3%	1	H.AVVY@GATALAAAVSLRSY@CKLQT#T.N	2
*	PARC_p629-05.12684.12684.2	2.5626	0.0801	2789.51	2790.893	74	157.5	21.7%	2	G.AT#ALAAAVSLRSY@CKLQTTNNNEI.V	2
*	PARC_p629-03.10324.10324.2	3.0501	0.2044	1064.33	1063.161	1	1248.5	83.3%	51	A.T#ALAAAVSLR.S	2
*	PARC_p629-02.09553.09553.1	2.8354	0.2428	1064.61	1063.161	1	238.2	72.2%	34	A.T#ALAAAVSLR.S	1
*	PARC_p629-02.16225.16225.3	4.509	0.011	3842.78	3841.888	130	483.2	17.9%	1	L.FTSLS*SPS*QGCTLTIS*SQVPLGAGLGS*SATISVVVA.T	3
*	PARC_p629-03.13154.13154.3	3.6062	0.0491	3321.2	3320.318	8	452.4	20.8%	1	S.PSQGCTLTIS*S*QVPLGAGLGSS*ATISVVVAT#.S	3
*	PARC_p629-04.12105.12105.2	3.0053	0.0103	2485.63	2486.541	35	134.7	30.4%	1	P.SQGCTLT#ISSQVPLGAGLGS*SATI.S	2
*	PARC_p629-02.20575.20575.3	3.6482	0.0112	3837.68	3839.09	95	488.0	18.1%	1	Q.VPLGAGLGS*SATIS*VVVATSLLLAFGNIEPPS*SNSLQ.N	3
*	PARC_p629-03.16290.16290.3	3.7817	0.0253	3838.37	3839.09	15	554.7	18.1%	2	Q.VPLGAGLGSSAT#IS*VVVATSLLLAFGNIEPPS*SNSLQ.N	3
*	PARC_p629-02.14098.14098.2	2.5975	0.0485	2288.97	2290.244	67	161.3	28.9%	1	D.AIDGISKSAVLALT#S*ES*DKN.S	2

U **SPCC1682.03c** 8 11 25.2% 626 69240 5.1 |||sequence orphan|Schizosaccharomyces pombe|chr 3|||Manual

	Filename	XCorr	DeltCN	ObsM+H+	CalcM+H+	SpR	SpScore	Ion%	#	Sequence	
*	PARC_p629-01.15398.15398.1	1.8771	0.0528	970.47	970.884	37	84.0	43.8%	4	L.S*DSSSLVAD.L	1
*	PARC_p629-02.17515.17515.3	3.9837	0.0128	4096.76	4095.554	10	472.4	17.6%	1	E.SSS*SVSDSS*DLSSTSDSS*SGDESSDTARQSSSDT#ISSN.N	3
*	PARC_p629-02.12762.12762.2	2.616	0.0764	2284.85	2286.004	10	305.9	33.3%	1	T.ARQSSSDTIS*SNNVS*VS*VS*.L	2
*	PARC_p629-03.16949.16949.3	4.3273	0.0129	4404.35	4403.546	18	534.7	18.2%	1	E.AVESEVAPS*SSTPPT#VEEISYLHYDEPSQYYFSSPSKL.S	3
*	PARC_p629-05.08444.08444.2	2.5319	0.0201	1527.29	1527.417	41	313.7	45.8%	1	V.EDDNTGVS*DDNKK.S	2
*	PARC_p629-02.11892.11892.3	4.0138	0.0236	3978.59	3979.137	9	410.4	18.6%	1	T.FSANES*T#VVANVPDSYLTKNDEEDSASLASLKVAPK.Y	3
*	PARC_p629-05.13566.13566.3	3.5661	0.0879	3637.01	3635.629	7	448.0	18.5%	1	A.NES*T#VVANVPDSYLT#KNDEEDSASLASLKVAP.K	3
*	PARC_p629-02.13973.13973.2	2.5326	0.0778	2024.65	2025.163	83	174.2	34.4%	1	F.TVMDLNPVT#CTPEISEK.T	2

U **SPBC20F10.01** 4 4 23.7% 194 20131 11.1 |gar1|SPBC25H2.01c|ribonucleoprotein |Schizosaccharomyces pombe|chr 2|||Manual

	Filename	XCorr	DeltCN	ObsM+H+	CalcM+H+	SpR	SpScore	Ion%	#	Sequence	
*	PARC_p629-02.21541.21541.3	3.6653	0.0143	3822.68	3824.177	63	362.5	17.7%	1	N.APIYLENKS*QIGKIDEVFGPMNQVY@FTVKPSE.G	3
*	PARC_p629-02.18643.18643.2	2.5398	0.0112	2206.63	2207.325	16	182.1	33.3%	1	T.VKPSEGIVS*S*SFKVGDKVY.L	2
*	PARC_p629-03.15986.15986.2	2.6384	0.0174	2206.73	2207.325	15	152.1	33.3%	1	T.VKPSEGIVSS*S*FKVGDKVY.L	2
*	PARC_p629-03.15845.15845.2	2.5806	0.0211	2206.79	2207.325	8	298.0	33.3%	1	T.VKPSEGIVS*SS*FKVGDKVY.L	2

U **SPCC1739.01** 8 10 23.6% 547 57978 9.6 ||SPCC1906.05|zinc finger protein|Schizosaccharomyces pombe|chr 3|||Manual

	Filename	XCorr	DeltCN	ObsM+H+	CalcM+H+	SpR	SpScore	Ion%	#	Sequence	
*	PARC_p629-03.16432.16432.3	3.6606	0.0563	3838.55	3839.863	121	471.8	18.4%	1	N.SRSVDEFNNS*SS*GFPSSLNGIPIAS*PPLATSPTSF.S	3
*	PARC_p629-03.16130.16130.3	3.7889	0.0197	3838.73	3839.863	1	660.8	20.6%	3	N.S*RS*VDEFNNSSSGFPSSLNGIPIAS*PPLATSPTSF.S	3
*	PARC_p629-02.17244.17244.3	4.0031	0.0212	4113.59	4112.127	163	300.9	15.4%	1	S.S*GFPSS*LNGIPIASPPLATSPTS*FSLASSASSTNLGGS*KG.L	3
*	PARC_p629-05.12396.12396.3	3.7724	0.0215	3060.08	3058.824	9	838.5	26.1%	1	C.S*ST#DTLS*SLSRLT#S*QDPLKAS*LPL.Q	3
*	PARC_p629-02.12804.12804.3	3.5197	0.0505	3860.96	3860.982	6	183.6	18.9%	1	R.SNSYAGAFPSVVSAS*LPT#KVDLNHQMDVSDEEQR.F	3
*	PARC_p629-03.13933.13933.3	3.8493	0.0267	3415.19	3416.534	27	403.4	21.6%	1	S.YAGAFPSVVSASLPT#KVDLNHQMDVS*DEEQ.R	3
*2	PARC_p629-05.09385.09385.2	2.7411	0.0293	2483.97	2485.468	9	182.3	31.6%	1	D.ES*ILGS*SPINRLSSSFKQY@T.S	2
*2	PARC_p629-05.09385.09385.2	2.7411	0.0293	2483.97	2485.468	9	182.3	31.6%	1	D.ES*ILGS*SPINRLSSSFKQYT#.S	2

U **SPBC16A3.08c** 3 3 23.6% 284 30949 10.1 |||nuclear telomere cap complex |Schizosaccharomyces pombe|chr 2|||Manual

	Filename	XCorr	DeltCN	ObsM+H+	CalcM+H+	SpR	SpScore	Ion%	#	Sequence	
*	PARC_p629-02.19746.19746.3	3.7356	0.0762	3837.8	3838.928	23	465.2	18.5%	1	S.VAS*KNLFDLLGEETPAATT#T#EKKT#AASRDKKR.S	23
*	PARC_p629-02.14787.14787.1	1.9431	0.0607	1535.63	1534.455	14	236.2	39.3%	1	E.NEGNTPS*GAQTPAAE.E	1
*	PARC_p629-04.18286.18286.3	3.6614	0.082	2360.48	2359.69	13	769.7	31.6%	1	S.QKKS*AAKESKPKKVLLDIEQ.T	3

U **SPCC1742.01** 27 54 23.5% 1563 157642 3.8 ||SPCC1795.13, SPCPB16A4.07c|glycoprotein |Schizosaccharomyces pombe|chr 3|||Manual

	Filename	XCorr	DeltCN	ObsM+H+	CalcM+H+	SpR	SpScore	Ion%	#	Sequence	
*2	PARC_p629-03.11497.11497.2	2.9268	0.0681	2559.17	2558.57	6	155.8	27.3%	1	N.DANYITASPVSNDASMTAIS*TFT#.S	2
*2	PARC_p629-03.11497.11497.2	2.9268	0.0681	2559.17	2558.57	6	155.8	27.3%	1	N.DANYITASPVSNDASMTAIS*T#FT.S	2
	PARC_p629-02.13437.13437.2	2.9369	0.0751	1993.79	1993.877	9	337.5	44.1%	5	G.YPGS*VT#T#TLTGAPSNGTV.I	2
	PARC_p629-02.12083.12083.3	3.5385	0.0451	3515.0	3515.067	2	374.7	23.1%	3	P.T#T#TNYGY@TTVT#T#GY@TGSTTLTTTVPHS*G.N	3
	PARC_p629-05.11259.11259.3	3.6565	0.0798	3654.02	3653.56	27	433.1	19.5%	3	P.TT#TNYGYTTVTTGY@T#GSTTLTTTVPHSGNETGP.T	3
*	PARC_p629-05.10354.10354.3	3.5155	0.0254	4083.44	4082.954	16	489.3	17.9%	1	T.T#T#TTVGYPGSVTTTLTGAPS*NGT#VIDTVEIPTTT#NY.G	3
*	PARC_p629-02.01747.01747.2	2.5175	0.021	2060.05	2059.934	12	188.4	36.7%	1	V.IDT#VEIPT#TTNYGY@TT.I	2
*	PARC_p629-01.16121.16121.2	2.9713	0.0282	2061.39	2059.934	9	213.6	40.0%	7	V.IDT#VEIPT#TTNY@GYTT.I	2
*	PARC_p629-05.12731.12731.3	3.6064	0.0714	4252.4	4252.995	135	265.1	16.0%	1	Y.VETPYPTTVTT#T#T#T#VGY@SGSVTTTLTGSGS*NSIVTET.V	3
*	PARC_p629-03.18523.18523.3	3.9836	0.0113	3839.72	3839.733	34	548.1	18.9%	1	V.TTVTTGYTGT#ET#S*T#VTVIPT#GTTGTTTVVIQTPT.T	3
	PARC_p629-02.01582.01582.2	2.9091	0.0558	1843.05	1842.83	1	431.7	44.4%	3	Q.PT#TGATGTAGTETQVTTGT.E	2
	PARC_p629-02.12331.12331.2	2.6051	0.0148	2576.61	2577.548	73	168.4	25.0%	3	A.TGTAGTET#QVTTGTETQATTATETQ.A	2
	PARC_p629-	3.5167	0.0138	3372.89	3373.232	10	430.1	21.7%	3	T.QVTTGTET#QATT#ATETQATTAT#EVQTTTGAT.G	3

	04.17451.17451.3										
	PARC_p629-05.11099.11099.3	3.5455	0.0224	4492.34	4492.303	110	216.4	14.6%	2	T.QATTAT#ETQATTAT#EVQTTT#GATGTAGT#ETQATTATEVQPTT.G	3
	PARC_p629-05.12642.12642.3	3.7506	0.0212	4173.29	4172.083	16	291.4	16.9%	6	E.VQTTTGATGTAGT#ETQATTATEVQPT#T#GATGTAGTETQVTT.A	3
*2	PARC_p629-02.13008.13008.2	2.7384	0.0129	2472.99	2472.545	6	236.1	36.4%	1	S.SSGYY@TTIVSSTVVSTVVPGS*TV.Y	2
*2	PARC_p629-02.13008.13008.2	2.7384	0.0129	2472.99	2472.545	6	236.1	36.4%	1	S.SSGYY@TTIVSSTVVSTVVPGST#V.Y	2
*	PARC_p629-02.09331.09331.1	2.0503	0.0362	1634.74	1635.393	1	392.2	54.5%	2	T.YTTS*STQYEPS*T#.V	1
*	PARC_p629-03.08493.08493.2	2.5594	0.0243	1654.47	1653.658	5	324.9	46.2%	1	T.YTT#SSTQYEPSTVV.T	2
*	PARC_p629-02.14516.14516.3	3.7311	0.0194	3860.84	3860.496	158	362.7	18.5%	1	V.VTTSY@YTTSVYT#S*APATETVSS*TEAPESS*TVT#.S	3
*	PARC_p629-03.14754.14754.3	3.9006	0.0189	4507.25	4507.153	69	262.4	17.6%	1	T.TSYYTT#SVYT#S*APATET#VSS*TEAPESST#VTSNPIYQGS.G	3
*	PARC_p629-02.10592.10592.3	3.5275	0.0134	3535.67	3535.012	46	237.6	19.4%	1	T.ETVSS*T#EAPESSTVT#SNPIY@QGS*GT#ST#W.S	3
*	PARC_p629-02.17530.17530.3	3.6915	0.0254	3823.28	3823.532	12	598.1	20.8%	1	G.SGT#STWS*T#VRQWNGSAT#YNYT#YYTTGGFTGG.N	3
*	PARC_p629-04.14945.14945.3	3.5226	0.0189	3823.52	3823.532	3	593.7	20.8%	1	G.SGT#STWS*T#VRQWNGS*ATYNYTYYTTGGFT#GG.N	3
*	PARC_p629-02.19015.19015.3	3.6966	0.0187	3823.58	3823.532	37	452.8	20.8%	1	G.SGT#S*T#WSTVRQWNGSAT#YNYTYYTTGGFT#GG.N	32
*	PARC_p629-05.11891.11891.3	3.6133	0.0141	3824.42	3823.532	7	648.5	22.5%	1	G.SGT#STWS*T#VRQWNGSAT#YNYTYYTTGGFT#GG.N	3
*	PARC_p629-02.19720.19720.3	3.8624	0.0115	3855.23	3856.48	300	351.2	17.2%	1	G.SGT#ST#WS*TVRQWNGSATY@NYTYYT#TGGFT#G.G	3

U **SPAC30D11.04c** 12 12 22.8% 1159 123974 9.5 |nup124||nucleoporin|Schizosaccharomyces pombe|chr 1|||Manual

	Filename	XCorr	DeltCN	ObsM+H+	CalcM+H+	SpR	SpScore	Ion%	#	Sequence	
*	PARC_p629-02.09515.09515.2	2.6222	0.0275	1067.15	1066.034	47	401.8	62.5%	1	T.AT#ADVQTNR.L	2
*	PARC_p629-02.18010.18010.3	3.6099	0.0842	3280.22	3281.397	26	440.7	20.7%	1	E.TEKKDNAPSAPAS*T#SGFSFGGFAPKTLEEK.E	3
*	PARC_p629-02.20011.20011.3	3.7306	0.0191	3837.17	3838.67	28	496.7	20.3%	1	S.SFGKPT#S*NIFNDKKTSENGLASST#VAS*ES*KPSA.P	3
*	PARC_p629-02.18244.18244.3	3.6471	0.0368	4098.77	4099.089	33	442.2	18.9%	1	A.S*TPFSFAKPNTENVT#TTAPQFS*FNFTKPNTDAKT#.N	3
*	PARC_p629-02.16992.16992.3	3.5914	0.0221	4095.23	4094.483	25	449.2	17.6%	1	N.FTKPNTDAKT#NLLPEKTFNEEAVKQKETEKEVPPT.G	3
*	PARC_p629-01.13964.13964.2	2.5627	0.0566	1813.93	1813.709	2	184.9	37.5%	1	N.T#ADGKDSASNLTTPS*PA.F	2
*	PARC_p629-04.13546.13546.3	3.6541	0.1083	4202.69	4204.049	168	256.1	15.4%	1	N.TADGKDS*ASNLTT#PSPAFSFGNNS*GVKASSNNNPSTNSST.A	32
*	PARC_p629-05.10455.10455.3	3.5991	0.0609	3318.17	3318.99	8	513.1	24.1%	1	A.FS*FGNNS*GVKASS*NNNPST#NSSTAPFS*F.G	3
*	PARC_p629-02.14442.14442.3	3.8397	0.0138	4106.0	4106.86	7	345.8	18.2%	1	N.KPAFS*FGSAT#S*KT#T#SEGTAPAASASAPAPTTS*AFSFGA.S	3
*	PARC_p629-03.17009.17009.3	3.6168	0.0514	3496.7	3496.4	119	243.9	16.7%	1	P.S*SAFAFGNQSGTNPAAPAGFGGITNTATNNPPS*T#.G	3
*	PARC_p629-03.10933.10933.2	2.8196	0.0162	1796.65	1796.728	7	551.8	43.8%	1	A.PAGFGGITNT#ATNNPPS*.T	2
*	PARC_p629-01.11371.11371.2	2.7939	0.0812	1726.63	1726.59	1	289.3	43.3%	1	G.SGEPSNPAS*NPPST#GF.S	2

U **SPAC9E9.09c** 5 5 22.5% 503 54768 6.7 |||alcohol dehydrogenase|Schizosaccharomyces pombe|chr 1|||Manual

	Filename	XCorr	DeltCN	ObsM+H+	CalcM+H+	SpR	SpScore	Ion%	#	Sequence	
*	PARC_p629-03.12856.12856.3	3.5721	0.0728	3855.74	3857.061	173	476.9	19.1%	1	L.ADCIEQNLEYLASIETLDNGKSIT#LARGDVQAAAD.C	3
*	PARC_p629-04.14360.14360.3	3.5729	0.0236	3825.41	3826.234	87	438.2	20.0%	1	G.QTIETDIKRFAY@TRHEPIGVCGQIIPWNFPF.L	3
*	PARC_p629-02.17098.17098.1	1.8643	0.0985	1443.84	1443.521	27	173.0	37.5%	1	A.AGVHTNNIT#NAIK.V	1
*	PARC_p629-02.19155.19155.3	3.8713	0.0648	3843.29	3842.181	3	600.5	22.0%	1	Y.NLLHHQIPFGGYKESGIGRELGSYGLT#NYTQTKA.V	3
*	PARC_p629-02.19754.19754.3	4.2499	0.039	3843.65	3842.181	1	707.5	22.7%	1	Y.NLLHHQIPFGGYKESGIGRELGSY@GLTNYTQTKA.V	3

U **SPBC646.16** 4 16 20.5% 244 26539 7.2 |||20S proteasome component |Schizosaccharomyces pombe|chr 2|||Manual

	Filename	XCorr	DeltCN	ObsM+H+	CalcM+H+	SpR	SpScore	Ion%	#	Sequence	
*	PARC_p629-02.11958.11958.3	4.657	0.0284	2213.33	2213.288	9	1156.3	40.3%	12	A.GFY@IGYKATSAGPKQTET#I.N	3
*	PARC_p629-03.11632.11632.3	4.1474	0.0144	2213.6	2213.288	7	1029.1	37.5%	2	A.GFYIGY@KATSAGPKQTET#I.N	3
*	PARC_p629-02.13635.13635.3	3.8382	0.0138	2214.2	2213.288	1	1204.2	40.3%	1	A.GFY@IGYKATSAGPKQT#ETI.N	3
*	PARC_p629-04.12797.12797.3	4.0024	0.0162	3594.41	3594.852	68	285.5	18.3%	1	E.T#GIQALMSSLSTDFKST#ELQIGVVEDDKPFR.V	3

U **SPBC1347.02** 3 3 19.7% 361 39302 4.8 |fkbp39||peptidyl-prolyl cis-trans isomerase|Schizosaccharomyces pombe|chr 2|||Manual

	Filename	XCorr	DeltCN	ObsM+H+	CalcM+H+	SpR	SpScore	Ion%	#	Sequence	
*	PARC_p629-05.11412.11412.3	3.7906	0.1517	4135.97	4137.41	12	431.7	18.6%	1	P.AVEESTDAS*IHLTMAS*IDAGEKS*NKPTTLLVKVRPR.I	3
*	PARC_p629-02.12709.12709.3	3.5458	0.0242	3014.78	3014.082	18	414.2	25.0%	1	A.VEEST#DAS*IHLTMASIDAGEKSNKPTT.L	3
*	PARC_p629-02.16967.16967.3	3.9494	0.0343	3840.5	3839.791	32	529.5	21.3%	1	A.SSNAPSS*PKT#RTLKGGVVVTDVKTGS*GAS*AT#NGKK.V	3

U **SPAC15A10.16** 13 13 19.4% 1385 154325 5.3 |fat1|SPAC15E1.01|actin interacting protein 3 homolog Fat1p|Schizosaccharomyces pombe|chr 1|||Manual

	Filename	XCorr	DeltCN	ObsM+H+	CalcM+H+	SpR	SpScore	Ion%	#	Sequence	
*	PARC_p629-02.13350.13350.2	2.536	0.0183	2392.21	2390.719	29	249.2	30.0%	1	P.LSRDAASHLIST#IETSIMKLL.V	2
*	PARC_p629-03.12254.12254.2	2.5369	0.1148	2211.93	2212.487	4	440.5	41.2%	1	T.S*IMKLLVVTKELLESLT#N.W	2
*	PARC_p629-04.11768.11768.2	2.7406	0.142	2471.63	2472.219	55	120.2	27.8%	1	E.LLES*LT#NWSKGT#VNDS*AVS*.S	2
*	PARC_p629-03.18179.18179.3	3.9339	0.0509	3840.11	3838.983	231	373.8	19.2%	1	R.S*VLEQT#LEKT#PS*SDALETNLPNVRELVMHLL.Q	3
*	PARC_p629-02.17576.17576.3	3.6855	0.0519	4098.89	4098.994	10	336.8	17.2%	1	S.RSNLPQMTREANDNDVNRRNSRGS*S*QGS*ISS*FR.N	3
*	PARC_p629-03.12575.12575.3	4.0508	0.0421	3759.62	3758.974	5	308.6	21.7%	1	D.RKFSPKLT#RTPSLT#KSLDPGT#PTSLKSPS*LR.K	3
*	PARC_p629-04.12242.12242.3	3.5314	0.0376	3759.77	3758.974	19	327.1	20.8%	1	D.RKFSPKLTRTPS*LT#KSLDPGT#PTSLKSPS*LR.K	3
*	PARC_p629-02.16933.16933.3	3.5217	0.0357	4109.21	4109.307	29	454.8	18.9%	1	S.LKSPSLRKSPSSFVQKDVYSRS*NS*LRIS*QANRSN.V	3
*	PARC_p629-03.15991.15991.3	3.511	0.0204	3471.05	3471.317	2	672.9	23.1%	1	V.T#RSVS*DHRILS*SS*TINDGEVAPPLPQRS*.R	3
*	PARC_p629-02.12214.12214.2	2.7894	0.0764	1938.17	1937.846	10	304.0	40.6%	1	S.SSEKIESGTSIST#S*DTK.G	2
*	PARC_p629-04.15275.15275.2	2.6405	0.0258	2220.29	2219.242	20	186.5	30.0%	1	K.IESGTS*ISTSDTKGGLANFEN.D	2
*	PARC_p629-05.09578.09578.3	4.1565	0.0266	3738.02	3737.766	2	321.7	21.9%	1	E.DS*EDKSTPNTGAS*AKLINDPSST#ITVSDVYPKK.P	3
*	p629-04ms3.10835.10835.2	2.5137	0.0809	2035.25	2035.914	1	456.9	46.4%	1	V.Y@KISFS*GDSY@ELNIE.D	2

U **SPAC589.10c** 2 2 19.3% 150 17215 9.8 |||ubiquitin family protein|Schizosaccharomyces pombe|chr 1|||Manual

U **SPAC6G10.11c** 2 2 19.3% 150 17258 9.8 |ubi3||ubiquitin family protein|Schizosaccharomyces pombe|chr 1|||Manual

	Filename	XCorr	DeltCN	ObsM+H+	CalcM+H+	SpR	SpScore	Ion%	#	Sequence	
	PARC_p629-02.09662.09662.2	2.5521	0.0714	1915.39	1914.229	45	109.2	36.7%	1	-.MQIFVKT#LTGKTITLE.V	2
	PARC_p629-02.10897.10897.2	2.5208	0.1162	1843.31	1842.758	3	483.2	50.0%	1	K.Y@Y@KVEDDGS*VKRL.R	2

U **SPBC1711.05** 3 5 18.8% 451 47428 4.4 |||chaperone activity |Schizosaccharomyces pombe|chr 2|||Manual

	Filename	XCorr	DeltCN	ObsM+H+	CalcM+H+	SpR	SpScore	Ion%	#	Sequence	
	PARC_p629-02.19078.19078.3	4.1756	0.0225	3844.19	3843.14	42	409.6	18.6%	2	S.DSSSSSSSESESSSEDNDSS*S*SSSDS*ESESSSEDS.D	3
*	PARC_p629-04.12693.12693.3	3.8146	0.0182	3319.7	3318.377	8	463.3	22.1%	2	S.EDS*DS*T#S*SSS*DSDSSSSSEDGNSNT#DT#.T	3
*	PARC_p629-02.17053.17053.3	3.6738	0.0222	3722.21	3723.094	16	251.8	18.8%	1	S.SSEDGNS*NTDT#TTSGEVSAQSS*T#NSTSSEES*TS.V	3

U **SPBC1604.20c** 7 7 18.6% 628 70099 7.1 |tea2|klp4|kinesin-like protein|Schizosaccharomyces pombe|chr 2|||Manual

	Filename	XCorr	DeltCN	ObsM+H+	CalcM+H+	SpR	SpScore	Ion%	#	Sequence	
*	PARC_p629-04.15780.15780.2	2.5295	0.0607	2502.87	2504.288	4	180.3	33.3%	1	T.PRRYS*T#MTGIRT#GPSQS*GT#.G	2
*	PARC_p629-03.16882.16882.3	3.6369	0.0126	4083.32	4082.984	48	466.5	18.9%	1	G.PSQS*GT#GS*IPYSPTSPLSRNFSNY@S*IPMLRSNST.Q	3
*	PARC_p629-05.15035.15035.3	3.5865	0.0251	4172.24	4171.999	75	308.5	17.2%	1	G.SIPY@SPTS*PLSRNFSNYS*IPMLRSNS*T#QT#NVNG.P	3
*	PARC_p629-04.12188.12188.2	2.5376	0.0618	2485.69	2486.577	169	104.5	30.6%	1	R.PALY@PSKDLDYLKNEYVNY@.E	2
*	PARC_p629-03.19130.19130.3	3.79	0.0421	3823.97	3822.806	46	444.7	20.8%	1	T.AAT#DFNTYSSRS*HAILQVFLIRNNPT#AHT#SQ.I	3
*	PARC_p629-02.09146.09146.1	1.806	0.0539	1636.75	1637.72	113	136.2	30.8%	1	L.LATISIES*NHTMET.T	1
*	PARC_p629-04.08958.08958.2	2.6249	0.0185	1638.57	1637.72	16	557.7	50.0%	1	L.LATISIES*NHTMET.T	2

U **SPBC12C2.05c** 4 5 18.2% 642 72217 5.3 |||DAG domain|Schizosaccharomyces pombe|chr 2|||Manual

	Filename	XCorr	DeltCN	ObsM+H+	CalcM+H+	SpR	SpScore	Ion%	#	Sequence	
*	PARC_p629-02.12801.12801.2	2.9325	0.072	2595.87	2595.749	2	289.8	32.5%	1	S.IEKEY@AQKLAS*LSNKYGEKKS.R	2
*	PARC_p629-03.18591.18591.3	3.5724	0.0275	3840.83	3840.994	3	676.3	21.0%	1	F.S*IEKS*LHDTLSQRTVEIQSEIAKNEPVLDS*AM.F	3
*	PARC_p629-02.18104.18104.3	3.7477	0.0132	4395.59	4396.104	1	748.5	22.8%	1	D.S*S*S*IY@QT#SLSNVKTEETT#PAEPASKVDGVVLYDFT#.G	3
*	PARC_p629-02.17713.17713.2	2.7769	0.0162	3316.09	3316.268	37	156.2	21.4%	2	D.GT#DGLIPAS*YVKLNDELNTS*VTLDGDSSY.V	2

U **SPACUNK4.11c** 2 3 18.1% 188 21505 9.3 |||sequence orphan|Schizosaccharomyces pombe|chr 1|||Manual

	Filename	XCorr	DeltCN	ObsM+H+	CalcM+H+	SpR	SpScore	Ion%	#	Sequence	
*	PARC_p629-03.11554.11554.2	2.7096	0.0945	2346.55	2347.416	31	187.2	28.9%	1	A.SFGLFNKELGEENVDEKDVS*.K	2
*	PARC_p629-04.08448.08448.1	2.1271	0.0117	1764.75	1764.944	58	177.2	38.5%	2	P.KES*NARKTKKNKNK.K	1

U **SPAC3A12.06c** 8 11 17.6% 743 82397 6.8 |||sodium/calcium exchanger|Schizosaccharomyces pombe|chr 1|||Manual

	Filename	XCorr	DeltCN	ObsM+H+	CalcM+H+	SpR	SpScore	Ion%	#	Sequence	
*	PARC_p629-04.12427.12427.2	2.7421	0.0887	2251.49	2252.158	1	600.2	47.2%	1	W.T#HRISKAFASVSSTSDS*T#V.H	2
*	PARC_p629-02.18892.18892.3	3.8365	0.0873	3855.8	3855.416	139	371.8	19.6%	1	S.VSST#S*DS*T#VHLFERGNT#LNDS*NDQILLT#C.N	3
*	PARC_p629-03.16653.16653.2	2.9851	0.08	2113.75	2114.426	6	466.4	37.5%	1	G.GMAIGELLGSAFFIVAIVAGS*.V	2
*	PARC_p629-02.09266.09266.2	2.9394	0.0206	2387.69	2388.348	15	176.4	36.8%	1	V.FFSGS*S*GVSVVITDENY@LPV.S	2
*	PARC_p629-02.13071.13071.2	2.7989	0.0377	2287.73	2288.06	1	500.2	40.6%	1	K.IHY@INDNDPS*NS*YSSYQ.H	2
*	PARC_p629-02.18065.18065.2	2.8033	0.0223	2214.65	2213.599	27	233.5	33.3%	1	W.YEKLMDVLAVPS*VLIFTLA.L	2
*	PARC_p629-02.17831.17831.2	2.8546	0.0469	2214.69	2213.599	11	281.4	36.1%	1	W.YEKLMDVLAVPSVLIFT#LA.L	2
*	PARC_p629-01.18972.18972.2	2.9963	0.1392	1828.61	1828.125	17	353.8	42.9%	4	P.WVSFIGFVLGIIWIS*.T	2

U **SPBC1289.04c** 6 15 17.3% 871 93773 7.9 |pob1||Boi family protein|Schizosaccharomyces pombe|chr 2|||Manual

	Filename	XCorr	DeltCN	ObsM+H+	CalcM+H+	SpR	SpScore	Ion%	#	Sequence	
*	PARC_p629-05.13041.13041.3	3.6394	0.0603	4137.65	4139.063	8	452.7	17.9%	1	R.S*DKDSEKLYSNNS*LSSSHSSILNGPLDS*LS*KPSVPS.N	3
*	PARC_p629-04.11623.11623.2	3.1146	0.0518	2212.51	2211.135	4	751.7	42.1%	10	S.SDLSNFNTIDADY@NNASAST.S	2
*	PARC_p629-02.11786.11786.2	2.7386	0.0204	1799.35	1798.744	31	297.5	40.0%	1	L.ATPS*SRNSKST#QSAVP.E	2
*	PARC_p629-02.19442.19442.3	3.5183	0.0235	3853.7	3854.702	25	461.6	18.2%	1	P.TDSIS*QNSGLPS*RLTEPISSPSTSS*IDVDKEGTS*.F	3
*	PARC_p629-04.17191.17191.2	2.599	0.0357	2215.39	2215.968	12	364.5	35.3%	1	S.S*NLEQGS*S*SS*VTKSPESM.P	2
*	PARC_p629-02.20477.20477.3	3.7921	0.0103	3859.52	3858.762	8	572.3	20.3%	1	T.KSPES*MPDPSAKAS*SPVTSKGVS*INEKS*AVNNY@.A	3

U **SPBC14C8.02** 11 63 16.6% 427 48550 9.3 |tim44||TIM23 translocase complex|Schizosaccharomyces pombe|chr 2|||Manual

	Filename	XCorr	DeltCN	ObsM+H+	CalcM+H+	SpR	SpScore	Ion%	#	Sequence	
*	PARC_p629-03.08667.08667.2	3.0503	0.0184	1651.33	1651.567	1	557.2	67.9%	23	Y.EKARSGTT#AASSFT#G.K	2
*	PARC_p629-03.08124.08124.2	3.0517	0.0208	1651.41	1651.567	5	329.8	53.6%	2	Y.EKARSGTTAAS*SFT#G.K	2
*	PARC_p629-05.13788.13788.2	2.6568	0.0649	1779.55	1779.741	39	166.3	40.0%	2	Y.EKARS*GTTAASSFT#GK.T	2
*	PARC_p629-05.12732.12732.2	2.5737	0.0248	1780.47	1779.741	79	162.1	36.7%	1	Y.EKARSGT#TAASSFT#GK.T	2
*	PARC_p629-04.08991.08991.2	2.957	0.0589	1780.65	1779.741	96	196.8	40.0%	13	Y.EKARSGTT#AASSFT#GK.T	2
*	PARC_p629-03.12630.12630.2	2.5366	0.013	1797.47	1798.662	12	355.6	50.0%	1	A.RS*GT#TAAS*SFTGKTVG.K	2
*	PARC_p629-02.12583.12583.2	2.9073	0.0223	1798.03	1798.662	10	387.4	50.0%	17	A.RS*GT#T#AASSFTGKTVG.K	2
*	PARC_p629-05.08492.08492.2	2.8113	0.0208	1918.83	1918.032	6	392.3	42.1%	1	R.SGTTAAS*SFTGKTVGKAGAK.I	2
*	PARC_p629-02.21511.21511.3	3.9855	0.0456	3839.36	3840.79	69	440.1	18.5%	1	T.ANTVAT#GVDT#ATKPVRETAFY@KTIKQT#MS*DGS.T	3
*	PARC_p629-02.18689.18689.3	4.2125	0.0195	3839.54	3840.79	11	536.5	20.2%	1	T.ANT#VAT#GVDTATKPVRETAFY@KTIKQT#MS*DGS.T	3
*	PARC_p629-02.12222.12222.2	2.8635	0.0224	1802.47	1801.952	10	404.1	42.9%	1	E.Y@IVPEVTEAYVKGDK.E	2

U **SPAC6G10.02c** 9 11 16.3% 1125 127755 5.5 |tea3||kelch repeat protein|Schizosaccharomyces pombe|chr 1|||Manual

	Filename	XCorr	DeltCN	ObsM+H+	CalcM+H+	SpR	SpScore	Ion%	#	Sequence	
*	PARC_p629-05.07594.07594.2	2.8388	0.0269	1766.49	1765.72	12	366.2	50.0%	1	L.FKLDLDS*CTVY@SH.N	2
*	PARC_p629-02.14642.14642.3	3.7359	0.206	4106.33	4106.197	101	174.2	19.4%	1	N.FKSNQWNLVS*T#QSPLPS*PRT#GHSMLLVDS*KLW.I	3
*	PARC_p629-02.15183.15183.3	4.7134	0.0106	3847.04	3846.725	125	537.2	18.8%	1	Y.EIEKPIAS*LPLPNGNDT#ISRSS*ES*SSPINESES.N	3
*	PARC_p629-02.21833.21833.3	3.6815	0.0116	3375.17	3376.237	1	508.5	23.1%	1	S.ESS*S*PINES*ESNSLLKLQSDFKFSNSDD.R	3
*	PARC_p629-02.18025.18025.3	3.5116	0.016	4113.41	4113.367	7	403.5	18.8%	1	G.LSKLLSS*INENKDNLKEFSKSKQKIS*Y@LESQLE.G	3
*	PARC_p629-05.13556.13556.2	2.5165	0.0386	2767.79	2768.767	1	305.3	31.0%	1	L.KHS*YSSVMT#EKSKLSDQVNDLT#.E	2

*	PARC_p629-04.14847.14847.2	2.957	0.0225	2768.27	2768.767	65	151.7	21.4%	2	L.KHSYSS*VMT#EKSKLSDQVNDLT#.E	2
*	PARC_p629-05.11669.11669.2	2.6152	0.058	2769.21	2768.767	7	220.2	26.2%	2	L.KHSYS*SVMT#EKSKLSDQVNDLT#.E	2
*	PARC_p629-05.09416.09416.3	3.6709	0.0325	3871.22	3871.737	5	630.9	20.3%	1	T.DVHLPT#DFS*ASSSPLRSY@FNEEDSFNNASAAHS.S	3

U **SPAC24H6.09** 10 13 16.3% 753 84486 7.8 |gef1||RhoGEF|Schizosaccharomyces pombe|chr 1|||Manual

	Filename	XCorr	DeltCN	ObsM+H+	CalcM+H+	SpR	SpScore	Ion%	#	Sequence	
*	PARC_p629-02.12432.12432.2	2.6473	0.0271	2065.69	2066.031	1	286.8	46.9%	1	R.YTLPSLPSSPSYNCPT#T#.L	2
*	PARC_p629-02.12958.12958.2	3.0096	0.0214	2695.75	2695.378	2	300.5	38.9%	1	R.Y@T#LPSLPSS*PS*Y@NCPTT#LR.K	2
*	PARC_p629-02.15261.15261.3	3.8525	0.0339	3349.73	3349.32	2	542.0	25.0%	4	Y.LPGSSTDEQEKRSSVTLASMPS*S*HSSTAS*L.L	3
*	PARC_p629-02.21732.21732.3	3.6063	0.0173	3350.21	3349.32	5	679.3	23.3%	1	Y.LPGSSTDEQEKRSSVTLAS*MPS*SHSST#ASL.L	3
*	PARC_p629-02.18778.18778.3	3.5286	0.0166	3350.78	3349.32	34	370.7	23.3%	1	Y.LPGSSTDEQEKRSSVTLASMPS*S*HSS*TASL.L	3
*	PARC_p629-04.13497.13497.3	3.8091	0.0165	3081.68	3081.235	17	515.8	21.3%	1	H.SSTASLLSPLDTT#SFSTKLDSTILALET#.D	3
*	PARC_p629-05.11149.11149.3	3.6365	0.0167	4162.1	4163.331	98	366.5	16.4%	1	S.LLS*PLDTTS*FS*TKLDSTILALETDESLSRTISYATT.S	3
*	PARC_p629-05.15362.15362.2	2.6369	0.194	1831.47	1830.78	3	175.3	39.3%	1	L.DS*TILALETDESLS*R.T	2
*	PARC_p629-04.15172.15172.2	2.5804	0.0158	2281.53	2281.248	70	101.1	27.8%	1	I.S*GINQKQKPSHKRGS*LS*AS.H	2
*	PARC_p629-05.09053.09053.2	2.5353	0.0148	2471.27	2472.388	2	191.1	31.6%	1	T.ANLPS*QIQT#SLNS*SILNPIT#.N	2

U **SPBC215.15** 3 3 16.2% 297 32568 5.4 |sec13||WD repeat protein|Schizosaccharomyces pombe|chr 2|||Manual

	Filename	XCorr	DeltCN	ObsM+H+	CalcM+H+	SpR	SpScore	Ion%	#	Sequence	
*	PARC_p629-02.07977.07977.1	1.8118	0.1154	1168.55	1169.155	12	300.9	50.0%	1	K.VFS*IENNQQ.T	1
*	PARC_p629-03.17107.17107.3	3.523	0.1124	4076.84	4076.483	1	328.2	18.2%	1	Q.S*PAAGPKKLATAGCDNLVKIWAFDAGVNNWILEDTLAG.H	3
*	PARC_p629-02.16836.16836.2	2.597	0.0718	2332.51	2333.483	6	274.4	31.6%	1	K.IWAFDAGVNNWILEDT#LAGH.V	2

U **SPAPJ696.02** 3 5 16.0% 430 46374 9.1 |||actin cortical patch component |Schizosaccharomyces pombe|chr 1|||Manual

	Filename	XCorr	DeltCN	ObsM+H+	CalcM+H+	SpR	SpScore	Ion%	#	Sequence	
*	PARC_p629-02.13505.13505.3	3.5202	0.0414	3595.7	3594.807	24	410.3	21.7%	1	E.LT#DFVIILNS*KAAVQT#FARLGSIT#LGGNLSI.A	3
*	PARC_p629-02.13964.13964.3	3.6715	0.0421	3327.92	3328.244	1	650.7	24.0%	1	R.RGDSYRSNRSRAHDDDDEDDYSFSRS*K.S	3
*	PARC_p629-02.17670.17670.2	2.9348	0.0427	2112.51	2113.175	1	606.0	47.1%	3	D.YS*FSRSKSLSRKTAGGS*L.R	2

U **SPAPB2C8.01** 13 41 15.7% 1220 124086 3.8 |||glycoprotein |Schizosaccharomyces pombe|chr 1|||Manual

	Filename	XCorr	DeltCN	ObsM+H+	CalcM+H+	SpR	SpScore	Ion%	#	Sequence	
*	PARC_p629-02.18164.18164.3	3.5988	0.0408	4396.1	4396.387	93	406.1	16.4%	1	V.VVETSANTITTTLY@S*GS*LEFTT#TLDSANGTTPATIEVVE.P	3
*	PARC_p629-02.17957.17957.3	3.6172	0.0155	4394.78	4394.371	22	468.3	19.1%	1	V.VETSANTITTTLY@SGSLEFT#T#T#LDSANGTTPATIEVVEP.A	3
*	PARC_p629-05.13430.13430.3	4.0565	0.0217	4395.56	4394.371	23	418.1	17.8%	1	V.VETSANTITTT#LYSGSLEFT#T#T#LDSANGTTPATIEVVEP.A	3
*	PARC_p629-05.10116.10116.2	2.7806	0.1425	2407.95	2406.524	1	308.8	30.4%	1	T.TLDSANGTT#PATIEVVEPAAGTVT.T	2
*	PARC_p629-05.11248.11248.3	3.5936	0.1566	4153.22	4152.017	114	313.7	15.3%	1	T.T#LDSANGTTPAT#IEVVEPAAGTVTTTIYS*GTTPFNT#T#.L	3
	PARC_p629-03.18762.18762.3	3.9367	0.0106	3838.82	3838.536	49	460.9	18.0%	6	A.GT#VTTTVYS*GTQEY@TTTLATASGT#VS*GT#VEVVD.T	3
	PARC_p629-04.11936.11936.3	3.8055	0.0699	3662.48	3663.43	1	468.2	21.8%	6	V.TTTVYS*GTQEYTTTLATAS*GT#VS*GT#VEVVDTA.A	3
	PARC_p629-04.12008.12008.3	3.6413	0.0219	3664.91	3663.43	19	426.0	18.5%	6	V.TTTVYS*GTQEYTTT#LAT#ASGT#VS*GTVEVVDTA.A	3
	PARC_p629-02.17180.17180.3	4.2181	0.0133	4094.03	4092.904	120	427.5	16.7%	6	V.T#TTVYSGTQEY@T#TTLATASGTVSGTVEVVDT#AAGT#VT.T	3
	PARC_p629-05.08939.08939.2	2.7411	0.0208	1783.45	1783.805	12	265.9	38.9%	6	L.ATASGT#VSGTVEVVDTAAG.T	2
	PARC_p629-03.13981.13981.3	3.7016	0.0119	3853.43	3854.785	4	467.7	18.4%	3	A.GT#VTT#TVYSGTQEY@TTTLAT#ASGTVSGTVEVIEPA.A	3
*	PARC_p629-04.12142.12142.2	2.6963	0.0876	1998.47	1998.031	3	228.7	30.6%	2	T.VSGTVEVIEPAAGTVT#ST#V.Y	2
*	PARC_p629-05.09263.09263.3	3.5203	0.0559	3885.53	3884.069	1	220.3	17.1%	1	S.GSVEYTTVLADASGSVTGTVEVVEPAAGTVTTT#VYSGSE.V	3

U **SPBC1706.01** 6 10 15.6% 809 89562 4.5 |tea4||src |Schizosaccharomyces pombe|chr 2|||Manual

	Filename	XCorr	DeltCN	ObsM+H+	CalcM+H+	SpR	SpScore	Ion%	#	Sequence	
*	PARC_p629-03.18319.18319.2	2.6526	0.0609	2399.89	2400.444	1	571.6	42.5%	2	S.AY@PDAENSNIS*KINISIAGNK.E	2
*	PARC_p629-03.13394.13394.3	3.5676	0.0698	3839.84	3839.678	111	199.4	18.5%	1	K.HKT#ASSAT#VDS*PLRRSLSVDAMQSNASFSSY@S*.S	3
*	PARC_p629-02.17964.17964.3	3.7911	0.0177	4097.75	4098.062	108	309.0	15.9%	1	L.RPSS*YSAVS*ES*SNFTHDVSRDNKEIS*LNAPKSII.V	3
*	PARC_p629-02.17448.17448.3	3.922	0.0143	4097.93	4098.062	44	334.1	16.7%	1	L.RPS*S*YSAVSES*SNFTHDVSRDNKEIS*LNAPKSII.V	3
*	PARC_p629-01.18085.18085.2	2.5528	0.0148	2115.65	2115.959	294	117.6	32.4%	1	S.SSQSDAQCTT#S*SVYITAE.R	2
*	PARC_p629-02.13190.13190.2	3.6176	0.0789	2486.73	2487.56	34	152.7	35.0%	4	S.LQY@DENPFKQSVVAELNNNSS.S	2

U **SPAC3C7.12** 4 4 15.6% 461 52607 4.6 |tip1|noc1|CLIP170 family plus end tracking protein Tip1|Schizosaccharomyces pombe|chr 1|||Manual

	Filename	XCorr	DeltCN	ObsM+H+	CalcM+H+	SpR	SpScore	Ion%	#	Sequence	
*	PARC_p629-04.11482.11482.2	2.762	0.0785	2455.71	2456.655	10	177.3	30.4%	1	K.CKLASSISSSPS*PKIDGTAASIGM.G	2
*	PARC_p629-02.11060.11060.2	2.752	0.0442	1899.61	1899.042	2	608.1	46.9%	1	S.NLSMNT#ISSTALTPTEK.I	2
*	PARC_p629-05.11682.11682.3	4.3187	0.1388	3156.05	3156.351	1	1270.8	31.2%	1	S.EDDLLFS*LQQERDYALNQVEILQER.V	3
*	PARC_p629-04.16636.16636.3	4.2929	0.0396	3843.26	3844.204	3	314.0	20.8%	1	S.EDDLLFS*LQQERDYALNQVEILQERVDTLMK.Q	3

U **SPAC3G9.12** 13 14 15.4% 1462 164094 6.6 |||localization spindle pole body |Schizosaccharomyces pombe|chr 1|||Manual

	Filename	XCorr	DeltCN	ObsM+H+	CalcM+H+	SpR	SpScore	Ion%	#	Sequence	
*	PARC_p629-04.14038.14038.3	3.6354	0.0163	3843.26	3842.27	56	563.3	20.8%	1	L.QKRVLNILIDLWHFNPS*EIEKSLIHLS*TTSK.S	3
*	PARC_p629-	2.6838	0.0312	1901.53	1901.826	123	120.8	28.6%	2	K.S*LIHLST#T#SKSAETR.I	2

02.12382.12382.2

*	PARC_p629-03.11355.11355.2	2.5471	0.1065	2250.45	2251.376	1	254.7	36.1%	1	P.ALY@INLENANPSVREEAKE.V	2
*	PARC_p629-02.09000.09000.1	1.8353	0.1426	1225.82	1225.219	5	116.4	50.0%	1	L.YINLENANPS*.V	1
*	PARC_p629-01.15375.15375.2	2.6858	0.073	2045.89	2045.193	43	171.3	25.0%	1	I.VQNS*ASSFQPAPFMTAVAT.L	2
*	PARC_p629-04.14644.14644.3	3.5831	0.0413	4163.36	4164.734	18	261.2	18.8%	1	E.LILPNLLKVCSVTKKLAS*QAANVTFAAILVNCGVLS*R.N	3
*	PARC_p629-03.11968.11968.3	3.5397	0.0731	2212.07	2213.345	25	931.2	33.3%	1	A.ANVT#FAAILVNCGVLS*RNL.S	3
*	PARC_p629-02.17904.17904.1	1.9975	0.0876	1197.32	1196.221	13	172.6	61.1%	1	K.RSVS*ATIKDE.S	1
*	PARC_p629-03.09624.09624.2	2.6871	0.0407	1569.73	1568.517	20	464.6	50.0%	1	E.FLSSNLT#DALY@SG.S	2
*	PARC_p629-02.11684.11684.2	3.1623	0.0224	2315.85	2317.197	66	297.7	34.4%	1	G.S*S*ICYSLIFS*HSLLDLT#.F	2
*	PARC_p629-02.13074.13074.2	2.5773	0.1631	2576.81	2576.676	12	147.1	27.5%	1	T.T#FFMPANEEILGSPAKDY@DIH.D	2
*	PARC_p629-02.17063.17063.3	3.7244	0.0184	4112.87	4111.929	222	304.0	16.9%	1	D.Y@MNT#PMNDFS*LSFS*EIKGGILES*PVES*PMT#GT.I	3
*	PARC_p629-03.15418.15418.2	2.5351	0.0883	2484.73	2484.396	16	133.2	30.6%	1	L.QGLY@EFT#QQRLQS*LNTETG.E	2

U **SPCC23B6.04c** 7 9 15.1% 1008 110725 7.2 |||CRAL/TRIO domain|Schizosaccharomyces pombe|chr 3|||Manual

	Filename	XCorr	DeltCN	ObsM+H+	CalcM+H+	SpR	SpScore	Ion%	#	Sequence	
*	PARC_p629-03.13250.13250.2	2.7394	0.1401	2507.51	2508.504	5	284.2	31.8%	2	A.AKGPAS*NVRPARPNVS*TSSTSNE.V	2
*	PARC_p629-02.10059.10059.2	2.7939	0.0445	1069.35	1068.111	7	584.1	75.0%	1	S.S*SPASKMRN.T	2
*	PARC_p629-02.10513.10513.2	2.547	0.0566	2147.87	2148.268	1	404.7	41.2%	1	S.S*RFISDEKAVTEDAMKTE.H	2
*	PARC_p629-02.13041.13041.2	2.7231	0.0123	2301.67	2302.014	2	269.0	40.0%	1	Q.RQRQS*VS*SY@DT#IVPS*Q.F	2
*	PARC_p629-02.18387.18387.3	4.216	0.0353	4075.4	4074.854	64	434.2	19.1%	2	K.VVQNSPPS*LLST#DNKIPESGSDHPS*SQDNS*SKAS*L.V	3
*	PARC_p629-02.17309.17309.3	3.5579	0.0173	3146.18	3145.079	57	451.0	22.1%	1	P.S*SQDNSS*KASLVENS*QTQSSTPRKPLP.T	3
*	PARC_p629-04.15803.15803.3	3.6859	0.0255	4202.63	4202.215	11	446.9	18.4%	1	S.LTNRS*SSPTVTPTVNT#DRQPKTLT#NS*ADELSPQKR.R	3

U **SPBC244.01c** 6 8 14.2% 660 74473 6.8 |sid4||SIN component |Schizosaccharomyces pombe|chr 2|||Manual

	Filename	XCorr	DeltCN	ObsM+H+	CalcM+H+	SpR	SpScore	Ion%	#	Sequence	
*	PARC_p629-04.11604.11604.3	3.9603	0.027	3663.08	3662.712	17	237.6	20.2%	1	V.PPSGYPLTSSTCVSSIS*QPIQST#DCQKAQENL.S	3
*	p629-05ms3.09911.09911.2	2.5873	0.0554	1716.49	1717.755	99	218.2	35.7%	1	G.YPLT#SSTCVSSISQP.I	2
*	PARC_p629-01.17033.17033.2	3.0373	0.1025	1859.95	1858.881	1	731.1	53.6%	1	K.EIQILRHT#SQNGDQE.S	2
*	PARC_p629-02.09013.09013.2	2.7163	0.0792	1989.29	1989.798	33	235.0	34.4%	1	T.S*QNGDQESNESSKNAIT#.T	23
*	PARC_p629-02.12695.12695.2	2.9396	0.0704	2474.67	2473.551	112	138.0	31.6%	2	N.T#NNLDAKLS*DESELMIEKNK.S	2
*	PARC_p629-02.18659.18659.3	3.6685	0.0244	3375.83	3375.319	10	369.4	21.2%	2	D.ES*ELMIEKNKSYSTPAS*ST#IPT#FHTSQ.P	3

U **SPBC651.09c** 6 13 14.1% 560 63680 5.6 |||RNA polymerase II associated Paf1 complex |Schizosaccharomyces pombe|chr 2|||Manual

	Filename	XCorr	DeltCN	ObsM+H+	CalcM+H+	SpR	SpScore	Ion%	#	Sequence	
*	PARC_p629-03.12836.12836.3	3.6021	0.0292	3345.92	3346.933	5	466.1	19.6%	1	H.DDLDDVLSSS*S*DEDNNENVGQDY@AEESGG.E	32
*	PARC_p629-01.10783.10783.2	2.9398	0.2205	1873.73	1873.759	1	219.1	40.6%	2	E.NVGQDY@AEESGGEGNEK.S	2
*	PARC_p629-02.01961.01961.2	2.8856	0.1184	1760.37	1759.656	1	584.0	53.3%	1	N.VGQDY@AEESGGEGNEK.S	2
*	PARC_p629-02.08447.08447.1	2.3246	0.0407	1081.56	1080.104	11	82.3	50.0%	1	P.LT#SAAAGKRD.K	1
*	PARC_p629-02.01934.01934.2	2.6024	0.0664	2115.39	2116.185	39	195.7	35.0%	7	R.LS*AAAT#ATPMSAPTSVLTGTS.P	2
*	PARC_p629-03.12951.12951.3	3.5091	0.0352	3776.57	3777.619	4	226.5	18.2%	1	L.S*AAATAT#PMSAPTSVLTGTSPQPS*PS*LSTSIMS*T#.P	3

U **SPAC9G1.06c** 7 9 14.0% 886 98263 9.3 |cyk3||src |Schizosaccharomyces pombe|chr 1|||Manual

	Filename	XCorr	DeltCN	ObsM+H+	CalcM+H+	SpR	SpScore	Ion%	#	Sequence	
*	PARC_p629-02.12761.12761.2	2.8024	0.0433	2471.61	2471.392	33	138.1	30.0%	2	S.PQKS*SIDT#PITSSDQGLT#PDL.V	2
*	PARC_p629-02.12918.12918.2	2.8085	0.0247	2472.11	2471.392	11	179.0	35.0%	1	S.PQKS*S*IDTPITSSDQGLT#PDL.V	2
*	PARC_p629-03.18410.18410.3	3.5209	0.0338	3821.69	3823.089	21	380.2	18.8%	1	S.DSLKHQKSHPMLNSLGSSLS*LKKS*VSRPPSSMS*.R	3
*	PARC_p629-05.09139.09139.2	3.138	0.0101	2679.05	2679.763	21	186.9	33.3%	1	P.SMASS*VLSPSDYFPQHRRFQS*A.P	2
*	PARC_p629-03.19122.19122.3	3.6477	0.028	3374.21	3374.533	43	369.7	22.0%	1	K.DDLKRT#KTYT#KAEFAAKREEIFNKLS*.M	3
*	PARC_p629-02.02179.02179.2	2.5766	0.0139	1825.49	1826.638	40	294.7	42.3%	1	S.AYACQPST#S*IS*NTR.L	2
*	PARC_p629-02.21953.21953.2	2.6579	0.0461	1828.73	1827.826	72	292.8	43.3%	2	P.STSISNT#RLAIQT#PTG.N	2

U **SPCC1672.11c** 8 10 13.8% 1315 148786 6.6 |||P-type ATPase |Schizosaccharomyces pombe|chr 3|||Manual

	Filename	XCorr	DeltCN	ObsM+H+	CalcM+H+	SpR	SpScore	Ion%	#	Sequence	
*	PARC_p629-02.18493.18493.3	3.9931	0.0214	4080.83	4081.009	8	216.1	22.8%	1	S.KRSTS*SIAS*KNS*LNSYLASSLMSHDS*IFDGPGLGT#.S	3
*	PARC_p629-02.20452.20452.3	3.8093	0.012	3824.21	3825.661	10	473.9	21.0%	2	R.YNDGES*IAASRDYQS*LLRDNGSGVYSDEEEIT#.E	3
*	PARC_p629-02.16604.16604.3	3.9266	0.0115	3826.76	3825.661	105	380.0	18.5%	1	R.YNDGESIAAS*RDYQS*LLRDNGSGVYSDEEEIT#.E	3
*	PARC_p629-02.13289.13289.2	2.5812	0.0124	2196.73	2196.317	5	222.3	38.2%	1	P.VS*KIPATDQSMKELFS*FS.K	2
*	PARC_p629-02.17609.17609.3	4.1922	0.2336	4090.91	4091.999	162	318.4	18.2%	2	F.IIALIGFVFSSINLLTLGVPIAT#IIIRALDLITIVVPP.A	3
*	PARC_p629-01.15467.15467.1	2.1697	0.09	1484.33	1484.58	1	414.7	54.2%	1	D.KT#GTLTEDGLDIM.G	1
*	PARC_p629-03.11947.11947.3	3.6208	0.1697	3068.78	3070.227	2	572.7	23.2%	1	G.TLTEDGLDIMGVS*VIEGSELGDLRSNSGN.L	3
*	PARC_p629-03.16095.16095.3	3.6224	0.0534	3825.11	3823.834	26	446.3	20.7%	1	N.SGNLCS*KDLLS*NDS*PSNLLYT#MATCHMLRY.V	3

U **SPBC2D10.11c** 3 17 13.7% 379 43371 4.6 |||nucleosome assembly protein |Schizosaccharomyces pombe|chr 2|||Manual

	Filename	XCorr	DeltCN	ObsM+H+	CalcM+H+	SpR	SpScore	Ion%	#	Sequence	
*	PARC_p629-04.10944.10944.3	3.5746	0.335	3115.58	3116.338	188	250.3	19.4%	1	S.WNGFAAQT#PQNTPSSDVHLSKAALEKAR.Q	32
*	PARC_p629-04.09920.09920.2	2.922	0.0555	1763.53	1764.852	225	208.5	38.5%	15	L.EKARQTLQS*QLEDK.S	2

*	PARC_p629-02.10654.10654.2	2.8277	0.1054	1595.51	1595.671	1	697.2	65.4%	1	S.S*GYIESLAPAVQNR.I	2

U **SPAC1751.01c** 6 6 13.5% 720 78726 8.1 |gti1||gluconate transporter inducer Gti1|Schizosaccharomyces pombe|chr 1|||Manual

	Filename	XCorr	DeltCN	ObsM+H+	CalcM+H+	SpR	SpScore	Ion%	#	Sequence	
*	PARC_p629-03.17937.17937.2	2.5279	0.1467	2446.39	2447.492	170	133.2	21.7%	1	V.NISSPSAPSLS*DNLFPGSSFSASS.Q	2
*	PARC_p629-02.15500.15500.3	3.5903	0.026	2775.89	2774.789	7	682.5	25.0%	1	I.SSPS*APSLSDNLFPGSSFSASSQQNPS.S	3
*	PARC_p629-02.19646.19646.3	3.6623	0.0133	3822.5	3823.995	8	615.3	21.9%	1	H.YCSS*SSLSYLHDTERALVGS*LTDSYGFKKSGLI.K	3
*	PARC_p629-02.20163.20163.3	3.7566	0.0265	3839.39	3840.819	21	614.6	20.2%	1	Y.CSSS*S*LSY@LHDTERALVGS*LTDSYGFKKSGLI.K	3
*	PARC_p629-04.11702.11702.2	2.5794	0.0219	2440.13	2441.573	5	191.0	26.1%	1	Q.GLTLST#SPISGVESSSLSSAISRN.Q	2
*	PARC_p629-01.17317.17317.2	2.511	0.1073	1855.47	1855.88	6	440.1	42.9%	1	A.IS*RNQSNLSSFQQQQ.Q	2

U **SPCC962.01** 9 10 13.1% 1429 156031 7.0 ||SPCP31B10.09|C2 domain|Schizosaccharomyces pombe|chr 3|||Manual

	Filename	XCorr	DeltCN	ObsM+H+	CalcM+H+	SpR	SpScore	Ion%	#	Sequence	
*	PARC_p629-04.10691.10691.3	3.6181	0.0703	2651.72	2651.58	26	389.1	22.9%	1	R.AS*S*S*GVNGTNGGMKQVSPVSTARTS.I	3
*	PARC_p629-02.19435.19435.2	2.5687	0.0188	2979.89	2980.11	12	308.0	26.0%	1	S.S*KS*ENVPEPAGEKVSMPEKQDLQSAL.P	2
*	PARC_p629-05.10556.10556.2	2.8597	0.0832	2251.95	2251.09	1	590.3	50.0%	1	I.LET#YS*S*YKLT#GQWWQ.S	2
*	PARC_p629-02.16053.16053.2	2.5166	0.0572	1798.83	1797.998	21	327.1	36.1%	1	Y.DLSAMMAGIASGT#AVGVVG.L	2
*	PARC_p629-02.11907.11907.2	2.8875	0.1176	1769.25	1767.985	2	512.3	50.0%	1	E.NNKKS*SIKVAVFDVK.S	2
*	PARC_p629-04.11710.11710.2	2.7269	0.0465	2144.53	2146.029	37	240.7	35.3%	1	L.RLNTDNS*S*KQSSENVQSA.T	2
*	p629-03ms3.10435.10435.2	2.5873	0.09	2236.65	2236.929	78	210.8	29.4%	2	S.ENVQSAT#DPT#TPAKDNS*T#.S	2
*	PARC_p629-05.15091.15091.3	3.559	0.0919	2361.59	2360.506	12	758.8	31.2%	1	V.IS*VNEALQY@PSGFALISIVSA.D	3
*	PARC_p629-04.16242.16242.3	3.7917	0.0213	4083.92	4083.498	8	547.5	19.3%	1	T.LNPMESYINSGS*LHFMLQDGQNLPIGDIRSSDPFVV.L	3

U **SPBC16E9.11c** 5 5 13.1% 786 89260 8.8 |pub3||ubiquitin-protein ligase |Schizosaccharomyces pombe|chr 2|||Manual

	Filename	XCorr	DeltCN	ObsM+H+	CalcM+H+	SpR	SpScore	Ion%	#	Sequence	
*	PARC_p629-02.13147.13147.3	3.5372	0.019	2212.16	2213.457	12	531.7	31.9%	1	E.GFEVTVKPS*SVISIRLFDQ.K	3
*	PARC_p629-04.14120.14120.3	4.1599	0.0449	4522.52	4523.042	125	156.8	15.9%	1	R.TTPRPTATT#NTSNQSTSNST#RNGTS*AAT#SNGTGTGAGT#GAS*H.R	3
*	PARC_p629-01.17725.17725.2	2.9346	0.077	2059.89	2058.95	17	184.2	35.3%	1	R.S*S*PVTNRQTNNTSALSNS.N	2
*	PARC_p629-05.07839.07839.2	2.7496	0.0113	1782.35	1781.756	16	232.2	46.2%	1	A.VDNY@TLQINPHSS*I.N	2
*	PARC_p629-02.13801.13801.2	2.6843	0.0289	2039.65	2039.054	1	373.1	50.0%	1	N.PHS*S*INPEHLNYFRF.I	2

U **SPCC757.02c** 2 2 13.1% 405 46653 6.4 |||hypothetical protein|Schizosaccharomyces pombe|chr 3|||Manual

	Filename	XCorr	DeltCN	ObsM+H+	CalcM+H+	SpR	SpScore	Ion%	#	Sequence	
*	PARC_p629-01.11973.11973.2	2.625	0.0456	2477.51	2478.77	11	204.7	28.3%	1	M.AEEGY@VAIVTGATGLNGAAIIKRL.S	2
*	PARC_p629-03.18203.18203.3	3.5328	0.0279	3856.07	3856.822	1	724.2	22.3%	1	H.SWS*RT#WPKIAEY@FGVEVPKNQFATDFT#LS*.T	3

U **SPAC688.07c** 6 9 12.9% 1038 113939 4.9 |||sequence orphan|Schizosaccharomyces pombe|chr 1|||Manual

	Filename	XCorr	DeltCN	ObsM+H+	CalcM+H+	SpR	SpScore	Ion%	#	Sequence	
*	PARC_p629-02.12766.12766.2	2.758	0.0535	2449.89	2449.343	1	441.5	36.8%	1	E.LDNTSEKATLET#S*SKPVT#EQ.A	2
*	PARC_p629-03.08622.08622.2	2.628	0.1941	1921.07	1920.818	27	205.2	40.0%	1	E.ELSDS*LS*NDFGIDAEK.E	2
*	PARC_p629-02.13141.13141.3	3.557	0.0391	2288.15	2289.168	12	532.2	30.3%	1	N.AT#NSEVET#VTNDPSFSQQPG.T	3
*	PARC_p629-02.14889.14889.2	2.711	0.0357	1974.97	1973.929	10	326.6	40.6%	2	I.SSNS*STHKSHSEPLNRD.E	2
*	PARC_p629-03.16659.16659.3	3.9197	0.0574	4203.53	4202.239	7	441.4	18.6%	2	Q.QS*T#TSPSINSGASADAVSSGISKKADNSETNLNYFATLDA.F	3
*	PARC_p629-03.12137.12137.2	2.7173	0.0335	2589.27	2589.666	32	161.9	32.5%	2	D.ILHLFKKKAKSEQSNAKSS*S*S*.S	2

U **SPAC2H10.01** 3 3 12.9% 480 51135 5.7 |||involved in transcriptional regulation|Schizosaccharomyces pombe|chr 1|||Manual

	Filename	XCorr	DeltCN	ObsM+H+	CalcM+H+	SpR	SpScore	Ion%	#	Sequence	
*	p629-02ms3.09267.09267.2	3.0031	0.0536	2288.73	2289.275	7	226.3	34.2%	1	P.YRPVAVSES*SSRS*SFSMGSS.E	2
*	PARC_p629-03.17171.17171.3	3.8231	0.0119	4077.74	4077.168	62	320.9	17.7%	1	E.MTLSS*SASFGASVSPKSTPGSNSTGAAVDTNSVHSNGGSDLD.Y	3
*	PARC_p629-02.13102.13102.2	3.2358	0.0366	2593.07	2593.229	38	168.6	28.3%	1	L.S*S*S*ASFGASVSPKS*TPGSNST#GAA.V	2

U **SPAC15A10.02** 3 4 12.9% 450 49196 9.2 |taf12||transcription factor TFIID complex |Schizosaccharomyces pombe|chr 1|||Manual

	Filename	XCorr	DeltCN	ObsM+H+	CalcM+H+	SpR	SpScore	Ion%	#	Sequence	
*	PARC_p629-04.10603.10603.2	2.5903	0.1186	1780.63	1780.983	20	251.2	40.0%	1	V.KIAQFT#AAISNGIRSI.Q	2
*	PARC_p629-02.15848.15848.2	2.5352	0.0321	2768.83	2768.461	23	187.9	26.1%	1	N.RQAS*S*ANGNNTGTSTPVNAS*TDT#R.K	2
*	PARC_p629-02.12124.12124.2	3.0094	0.0335	1798.49	1798.776	4	574.2	41.2%	2	V.GVS*AANVSNESTELATSA.T	2

U **SPAC29B12.01** 8 8 12.8% 1604 183048 8.3 ||SPAC3G6.12|SNF2 family|Schizosaccharomyces pombe|chr 1|||Manual

	Filename	XCorr	DeltCN	ObsM+H+	CalcM+H+	SpR	SpScore	Ion%	#	Sequence	
*	PARC_p629-02.17529.17529.3	3.7279	0.0156	4078.31	4077.566	14	249.3	20.3%	1	P.EY@GAT#GT#PVNANNASRVDY@AT#T#AANVPEEY@AND.Y	3
*	PARC_p629-04.16511.16511.3	4.0341	0.0592	4398.29	4398.282	3	602.5	18.9%	1	T.ATSENEDLKDDLSLADLS*S*KKT#ANS*QATENNNTPSKAK.V	32
*	PARC_p629-03.16962.16962.3	4.1525	0.033	4399.46	4398.282	112	379.8	16.9%	1	T.ATSENEDLKDDLSLADLS*SKKTANS*QAT#ENNNTPS*KAK.V	3
*	PARC_p629-02.15209.15209.2	2.66	0.0349	2203.53	2204.152	64	283.4	35.3%	1	S.DKSRAKLS*SDT#NKDSEKN.D	2
*	PARC_p629-02.10608.10608.1	1.8883	0.0605	1758.83	1757.735	6	185.0	39.3%	1	T.S*KNANGET#KSTPKKS.K	1
*	PARC_p629-02.16449.16449.3	3.6944	0.0306	4349.6	4350.261	27	275.0	17.6%	1	T.ELY@S*HFVGRKMDREQDLPSAT#NTAS*VSEINFDSDE.E	3
*	PARC_p629-02.20576.20576.3	4.2075	0.0394	3827.06	3826.786	89	443.0	19.2%	1	L.THVLEES*DS*QLDLT#LLDS*VLVQRAS*APPIDI.Y	3
*	PARC_p629-02.17242.17242.3	3.8553	0.0708	4095.47	4094.532	1	664.1	20.7%	1	V.TEWQTRPELFVFLLSTRAGGLGINLT#AADTVIFYDS.D	3

U **SPBC13E7.03c** 6 6 12.8% 713 77869 9.2 |||SAM domain |Schizosaccharomyces pombe|chr 2|||Manual

	Filename	XCorr	DeltCN	ObsM+H+	CalcM+H+	SpR	SpScore	Ion%	#	Sequence	
*	PARC_p629-01.16726.16726.2	2.9072	0.0209	2100.65	2099.175	2	365.7	42.1%	1	S.QEIST#LLSSSLTSSPSNSGL.S	2
*	PARC_p629-02.12886.12886.3	3.9747	0.02	3892.97	3892.96	13	245.0	18.1%	1	S.TLLSS*SLTSSPSNSGLSLDKSLPSS*PKGDSPSLSS*SL.P	32

*	PARC_p629-01.14336.14336.2	2.6285	0.0715	2102.39	2102.097	175	132.2	30.6%	1	G.DS*PSLSSSLPS*LTTKSNLS.G	2
*2	PARC_p629-02.16682.16682.3	4.1614	0.0189	4108.88	4108.13	171	401.6	16.0%	1	P.ASSASTSPRNTPT#PSNNGTSINANVTSS*LTSNSTGKTSKT.T	3
*2	PARC_p629-02.16682.16682.3	4.1613	0.0189	4108.88	4108.13	199	389.4	16.0%	1	P.ASSASTSPRNT#PTPSNNGTSINANVTSS*LTSNSTGKTSKT.T	3
*	PARC_p629-03.12385.12385.2	2.9169	0.0409	2591.71	2592.241	34	162.1	28.6%	1	N.NGT#S*INANVTS*S*LTS*NSTGKTS.K	2

U **SPAC23A1.10** 2 2 12.8% 460 49675 9.0 |ef1a-b||translation elongation factor |Schizosaccharomyces pombe|chr 1|||Manual

U **SPCC794.09c** 2 2 12.8% 460 49661 9.0 |ef1a-a||translation elongation factor 1 |Schizosaccharomyces pombe|chr 3|||Manual

U **SPBC839.15c** 2 2 12.8% 460 49675 9.0 |ef1a-c||translation elongation factor |Schizosaccharomyces pombe|chr 2|||Manual

	Filename	XCorr	DeltCN	ObsM+H+	CalcM+H+	SpR	SpScore	Ion%	#	Sequence	
	PARC_p629-02.12039.12039.3	3.515	0.0945	3326.36	3325.59	32	357.2	19.0%	1	A.RFEEIVKET#S*NFIKKVGFNPKT#VPFV.P	3
	PARC_p629-02.17887.17887.3	3.5678	0.154	4090.46	4090.928	139	335.8	18.0%	1	C.AS*FT#AQVIILNHPGQIS*AGY@S*PVLDCHT#AHIAC.K	3

U **SPBC16H5.02** 4 4 12.7% 942 102555 6.2 |pfk1||6-phosphofructokinase |Schizosaccharomyces pombe|chr 2|||Manual

	Filename	XCorr	DeltCN	ObsM+H+	CalcM+H+	SpR	SpScore	Ion%	#	Sequence	
*	PARC_p629-02.18239.18239.2	2.7209	0.0624	2105.79	2105.925	51	206.8	37.5%	1	L.S*LSPAAEDSEWRGQKS*S*.L	2
*	PARC_p629-02.13884.13884.3	3.6862	0.0475	3819.44	3818.941	192	262.2	16.9%	1	R.AAKNLIS*AGIDSIIVCGGDGSLT#GADIFRS*DWPGL.V	3
*	PARC_p629-02.15147.15147.3	3.8809	0.0614	4015.52	4014.133	54	353.9	17.6%	1	T.IKQSASAS*RRRVFVCEVQGGRS*GYIAT#VGGLIT#GA.S	3
*	PARC_p629-04.11684.11684.3	3.6691	0.0355	3556.91	3557.786	3	326.0	16.4%	1	N.DPS*SAVVIGIRGTGVSFSSVADVENNETEIEMR.R	3

U **SPAC1687.06c** 2 2 12.7% 134 14755 11.2 |rpl44|rpl28|60S ribosomal protein L28/L44|Schizosaccharomyces pombe|chr 1|||Manual

	Filename	XCorr	DeltCN	ObsM+H+	CalcM+H+	SpR	SpScore	Ion%	#	Sequence	
*	PARC_p629-03.08987.08987.2	4.5602	0.535	1863.69	1863.91	1	1457.7	68.8%	1	F.S*GLCNDKAVGVQANSPR.G	2
*	PARC_p629-03.08970.08970.3	4.2778	0.2623	1865.15	1863.91	1	1388.1	42.2%	1	F.S*GLCNDKAVGVQANSPR.G	3

U **SPAC20G4.08** 9 9 12.3% 1076 119231 5.3 ||SPAC4F10.01|sequence orphan|Schizosaccharomyces pombe|chr 1|||Manual

	Filename	XCorr	DeltCN	ObsM+H+	CalcM+H+	SpR	SpScore	Ion%	#	Sequence	
*	PARC_p629-02.11469.11469.2	3.0985	0.0863	3076.23	3074.984	8	262.5	28.0%	1	G.KS*HDGS*EAVESTFPEKKESSLS*AQQP.H	2
*2	PARC_p629-03.12550.12550.2	2.8349	0.0512	2490.57	2489.541	11	163.6	30.0%	1	S.SLS*AQQPHVDDQRSSLLS*LLN.A	2
*2	PARC_p629-03.12550.12550.2	2.8349	0.0512	2490.57	2489.541	11	163.6	30.0%	1	S.S*LSAQQPHVDDQRSSLLS*LLN.A	2
*	PARC_p629-03.12390.12390.2	3.521	0.0931	2696.01	2695.744	13	182.6	31.0%	1	S.TSEIIFQLS*GAQS*LSDPIKS*RF.H	2
*	PARC_p629-02.13484.13484.2	2.5744	0.015	2232.07	2233.311	84	230.9	33.3%	1	C.FLIDTGISAKEY@DFSYDGT.V	2
*	PARC_p629-01.16169.16169.2	2.5547	0.081	2119.95	2119.251	33	183.6	41.2%	1	P.IYNSTYELILAS*LNTSEP.V	2
*	PARC_p629-02.18333.18333.1	1.9128	0.145	1745.42	1745.799	152	88.6	35.7%	1	Y.NSTY@ELILASLNTSE.P	1
*	PARC_p629-03.12199.12199.2	2.5893	0.0169	2589.67	2590.481	8	216.2	33.3%	1	D.LTERHS*TAS*PS*TVNSGFSTPRS.Q	2
*	PARC_p629-02.10287.10287.2	2.5815	0.0724	1420.51	1419.501	1	607.8	65.0%	1	A.QLRY@SIQNVAH.A	2

U **SPAC13A11.01c** 7 7 12.2% 777 88208 5.5 |rga8|SPAC2F7.18c|GTPase activating protein |Schizosaccharomyces pombe|chr 1|||Manual

	Filename	XCorr	DeltCN	ObsM+H+	CalcM+H+	SpR	SpScore	Ion%	#	Sequence	
*	PARC_p629-02.15037.15037.2	2.626	0.052	2491.27	2491.423	65	220.1	30.0%	1	E.T#T#SEAITDLTT#VSSPQQQSLL.E	2
*	PARC_p629-04.16746.16746.2	2.5735	0.0222	2722.55	2723.592	21	260.6	26.2%	1	L.VNS*PLS*S*VHQLREALNHS*SSVT.K	2
*	PARC_p629-02.13506.13506.2	2.8098	0.0126	2292.27	2293.465	2	423.5	38.2%	1	I.THLNRLITLT#PNKETFT#I.N	2
*	PARC_p629-03.11263.11263.2	2.8652	0.0289	2315.71	2315.553	1	951.9	55.6%	1	L.TYGPSIFEELRKLNS*SKRV.S	2
*	PARC_p629-05.09235.09235.2	3.2936	0.0356	2082.77	2084.22	2	548.2	50.0%	1	G.PS*IFEELRKLNSS*KRV.S	2
*	PARC_p629-02.12674.12674.2	2.8998	0.0179	2083.43	2084.22	2	537.5	50.0%	1	G.PS*IFEELRKLNS*SKRV.S	2
*	PARC_p629-02.14859.14859.2	2.5781	0.1788	1782.75	1782.774	1	599.0	50.0%	1	S.SFEELQPSEIPAES*E.F	2

U **SPBC15D4.01c** 5 7 12.2% 633 71452 7.0 ||SPBC2D10.21c|kinesin-like protein|Schizosaccharomyces pombe|chr 2|||Manual

	Filename	XCorr	DeltCN	ObsM+H+	CalcM+H+	SpR	SpScore	Ion%	#	Sequence	
*	p629-04ms3.07607.07607.2	2.6224	0.0368	1717.35	1717.754	73	237.3	35.7%	1	L.SLKKSSTS*DKKS*IAG.I	2
*	PARC_p629-05.13339.13339.2	2.5105	0.2185	2213.63	2213.249	2	307.0	30.6%	1	V.DLAGS*ERT#RSAETSGLLLR.E	2
*	PARC_p629-03.18098.18098.2	3.0717	0.0111	2103.41	2103.958	1	631.2	53.3%	2	S.PS*HS*LLQKS*KNTSS*TK.A	2
*	PARC_p629-02.14114.14114.2	2.6061	0.0196	1802.41	1803.885	5	311.9	46.4%	2	S.S*TKALTSHLEQLQQE.N	2
*	PARC_p629-05.08359.08359.2	2.8385	0.0349	1783.65	1783.722	4	395.3	53.6%	1	E.ESIKES*SATQQNENQ.H	2

U **SPAC23G3.01** 6 7 11.4% 1210 137849 6.8 |rpb2|SPAC521.06|DNA-directed RNA polymerase II subunit Rpb2p|Schizosaccharomyces pombe|chr 1|||Manual

	Filename	XCorr	DeltCN	ObsM+H+	CalcM+H+	SpR	SpScore	Ion%	#	Sequence	
*	PARC_p629-02.14828.14828.3	3.5854	0.0424	4098.05	4099.065	24	214.3	18.2%	1	H.TGAQGDVT#RRYEINFGQIYLSRPTMT#EADGSTT#T#.M	3
*	PARC_p629-04.16784.16784.3	3.6379	0.0498	4076.57	4077.196	218	296.2	17.2%	1	I.GKIPIMLRSTFCILNGVSDS*ELY@DLNECPY@DQG.G	3
*	PARC_p629-04.07529.07529.2	2.8219	0.1101	1782.77	1782.091	4	260.3	46.4%	1	R.GS*RLISSMQIKLMAR.N	2
*	PARC_p629-03.13616.13616.3	3.7376	0.0253	3350.39	3351.579	202	207.9	21.2%	1	I.SS*MQIKLMARNT#ENSGQT#IRATLPYIR.S	3
*	PARC_p629-01.17752.17752.2	3.0294	0.06	1842.65	1841.903	60	190.9	43.3%	1	S.T#ESGIVDQVMVTTNQE.G	2
*	PARC_p629-03.12638.12638.2	3.404	0.0692	2486.89	2487.684	20	164.6	31.8%	2	A.LSGFEGDATPFTDVTVEAVS*KLL.R	2

U **SPBC1815.01** 2 2 11.4% 439 47436 6.7 |eno101|eno1|enolase|Schizosaccharomyces pombe|chr 2|||Manual

	Filename	XCorr	DeltCN	ObsM+H+	CalcM+H+	SpR	SpScore	Ion%	#	Sequence	
*	PARC_p629-02.10846.10846.1	1.8384	0.1067	1755.9	1754.853	3	247.0	42.9%	1	T.DFQIVGDDLT#VTNVK.R	1
*	PARC_p629-04.17726.17726.3	3.5327	0.0158	3839.12	3839.034	45	370.4	19.1%	1	V.MVSHRS*GETADTFISHLTVGIGAGQLKSGAPCRS*E.R	3

U **SPAC2G11.06** 2 5 11.3% 432 48402 6.7 |vps4||AAA family ATPase|Schizosaccharomyces pombe|chr 1|||Manual

	Filename	XCorr	DeltCN	ObsM+H+	CalcM+H+	SpR	SpScore	Ion%	#	Sequence	
*	PARC_p629-03.20190.20190.2	2.7384	0.0572	1828.41	1827.652	11	402.7	37.5%	4	R.VS*NGNVEGSNSPT#ANEA.L	2
*	PARC_p629-02.20318.20318.3	3.5194	0.0397	3822.98	3822.636	108	327.5	17.7%	1	W.SGILLYGPPGT#GKS*Y@LAKAVAT#EAGS*T#FFS*IS.S	3

U **SPBC27B12.08** 11 17 10.7% 1919 217689 6.5 |||conserved eukaryotic protein|Schizosaccharomyces pombe|chr 2|||Manual

	Filename	XCorr	DeltCN	ObsM+H+	CalcM+H+	SpR	SpScore	Ion%	#	Sequence	
*	PARC_p629-03.17458.17458.3	3.6888	0.0699	4076.54	4075.647	53	353.4	22.3%	1	I.ET#S*IQST#NEDLIKEIWKCIKS*NLNT#S*S*T#N.V	3

*	PARC_p629-05.08166.08166.1	1.8515	0.1571	1339.77	1338.297	42	101.0	36.4%	1	D.KS*IEAAANAVPS*.T	1
*	PARC_p629-03.18911.18911.3	3.7802	0.0141	3660.05	3658.82	10	474.1	18.5%	1	K.ISEIAVKTAMAST#ADILS*RSKGSAT#ALYS*QLL.D	3
*	PARC_p629-02.13637.13637.2	2.8618	0.0296	2288.01	2288.579	6	369.7	39.5%	2	N.LTLS*LMSIYVAAEENPLYVA.S	2
*	PARC_p629-02.14265.14265.2	2.5405	0.0887	1894.75	1895.987	1	464.0	46.4%	1	E.ENPLY@VAS*SIFSKLF.N	2
*	PARC_p629-03.16767.16767.2	2.571	0.1453	2215.09	2215.263	88	230.8	31.6%	1	I.CESSSDSLSVISRSNS*AKLP.Y	2
*	PARC_p629-02.07472.07472.1	1.8033	0.031	1047.67	1046.99	38	79.9	57.1%	1	V.T#ANT#LFSI.Y	1
*	PARC_p629-02.13606.13606.3	3.5092	0.0258	2869.67	2869.348	1	462.6	27.1%	1	T.LPDKVVS*LMIELLENLTAVNDPKLI.P	3
*	PARC_p629-04.14473.14473.3	3.8683	0.0585	3374.9	3375.691	61	295.9	19.2%	6	A.FVDDSSPEIVAMSLNIASNFIGTGLLKTES*Q.A	3
*	PARC_p629-03.09513.09513.2	2.5059	0.054	1703.67	1702.8	2	345.8	46.2%	1	P.EIVAMS*LNIAS*NFI.G	2
*	PARC_p629-02.12081.12081.2	2.9849	0.0587	2329.83	2330.597	6	395.3	39.5%	1	T.AFMSMVLVLT#LAAPILDS*EQ.L	2

U **SPBC11C11.02** 3 6 10.6% 670 75167 6.6 |imp2||FCH domain|Schizosaccharomyces pombe|chr 2|||Manual

	Filename	XCorr	DeltCN	ObsM+H+	CalcM+H+	SpR	SpScore	Ion%	#	Sequence	
*	PARC_p629-03.11591.11591.2	2.929	0.0507	2211.67	2212.337	15	532.9	41.2%	3	L.KES*VQVMKAEVDNMAKS*H.L	2
*	PARC_p629-02.16380.16380.3	4.1461	0.0378	3827.75	3828.673	1	902.3	23.5%	2	K.LT#SRPS*T#PNVGNTAPDALSSPRNDSPLTS*AADEQ.M	3
*	PARC_p629-02.01811.01811.2	2.5584	0.0374	2059.67	2059.207	18	209.1	36.1%	1	N.PTPAAPGAFPNSNT#LPPRY.N	2

U **SPCC550.15c** 2 7 10.6% 463 52251 6.2 |||zinc finger protein|Schizosaccharomyces pombe|chr 3|||Manual

	Filename	XCorr	DeltCN	ObsM+H+	CalcM+H+	SpR	SpScore	Ion%	#	Sequence	
*	PARC_p629-01.16446.16446.2	2.8059	0.039	2505.95	2506.536	2	384.6	41.7%	6	S.KFQRNSRIKKLQS*EDAS*S*I.A	2
*	PARC_p629-03.15142.15142.3	3.6641	0.0118	3447.2	3447.389	233	153.3	19.8%	1	D.FTTSYPDYAVKQDETVVEEDGSS*GEGDWED.V	3

U **SPBC337.06c** 2 2 10.6% 265 30432 5.0 |cwf15||complexed with Cdc5p |Schizosaccharomyces pombe|chr 2|||Manual

	Filename	XCorr	DeltCN	ObsM+H+	CalcM+H+	SpR	SpScore	Ion%	#	Sequence	
*	PARC_p629-02.10779.10779.3	3.611	0.22	1927.85	1926.993	1	617.7	42.2%	1	Q.NS*KLIEGFTSPSTDDKP.N	3
*	PARC_p629-02.18215.18215.1	2.0137	0.0561	1244.0	1243.28	17	157.4	50.0%	1	L.NKAS*SGSFQVK.R	1

U **SPAC7D4.11c** 3 3 10.1% 769 87511 5.0 |||hypothetical protein|Schizosaccharomyces pombe|chr 1|||Manual

	Filename	XCorr	DeltCN	ObsM+H+	CalcM+H+	SpR	SpScore	Ion%	#	Sequence	
*	PARC_p629-02.16646.16646.3	3.7101	0.0196	3894.83	3896.052	65	493.8	22.5%	1	L.KDEAEFY@SLFSKKIVEETDLVDLAY@QLT#KNS.Q	3
*	PARC_p629-05.11910.11910.3	4.3498	0.0259	3828.98	3829.124	259	400.3	18.3%	1	A.LACKVLDVFVDCEPT#IDLRRRLNS*LVNACSL.I	3
*	PARC_p629-02.20170.20170.2	2.5299	0.0937	1830.67	1829.79	6	152.0	40.0%	1	L.YEGNLNSS*LSLNEDPS.V	2

U **SPAC977.14c** 2 2 9.7% 351 39915 6.4 |||aldo/keto reductase|Schizosaccharomyces pombe|chr 1|||Manual

	Filename	XCorr	DeltCN	ObsM+H+	CalcM+H+	SpR	SpScore	Ion%	#	Sequence	
*	PARC_p629-02.11583.11583.2	2.5299	0.0454	2212.11	2213.42	20	441.3	39.5%	1	S.SLSPELYGCLGNSGLKVS*KL.I	2
*	PARC_p629-04.12823.12823.2	2.5125	0.0414	1818.25	1819.603	5	314.2	42.3%	1	I.RTFDT#ANCYS*AGVS*.E	2

U **SPCC970.10c** 3 3 9.6% 680 78053 6.3 |||zinc finger protein|Schizosaccharomyces pombe|chr 3|||Manual

	Filename	XCorr	DeltCN	ObsM+H+	CalcM+H+	SpR	SpScore	Ion%	#	Sequence	
*	PARC_p629-04.15043.15043.2	2.6686	0.0527	2768.91	2769.74	39	148.4	21.4%	1	L.DELDT#T#RS*QLHSIIKLPDRSSS.F	2
*	PARC_p629-02.19742.19742.3	3.6079	0.0179	3918.41	3919.136	1	481.0	23.4%	1	A.TREAANKIRLVDLNDLELQKDLST#YLS*KELAS*.T	3
*	PARC_p629-02.12238.12238.2	2.7389	0.04	1595.45	1594.473	1	1348.7	75.0%	1	S.T#IQNLEEKVS*Y@.L	2

U **SPAC23G3.06** 3 3 9.6% 508 55759 9.1 |||ribonucleoprotein |Schizosaccharomyces pombe|chr 1|||Manual

	Filename	XCorr	DeltCN	ObsM+H+	CalcM+H+	SpR	SpScore	Ion%	#	Sequence	
*	PARC_p629-01.17277.17277.2	3.0414	0.0209	2117.63	2117.134	88	171.9	41.7%	1	H.LS*S*LISGLAPS*DLNAMSLG.L	2
*	PARC_p629-03.21742.21742.2	2.5478	0.0359	1800.97	1799.906	24	228.2	36.7%	1	T.LKS*AAEISMGTEITEE.D	2
*	PARC_p629-04.09185.09185.3	3.7115	0.058	1640.42	1639.81	1	978.0	44.2%	1	H.STPKYGLIYHAS*LV.G	3

U **SPBC27.08c** 3 3 9.4% 490 54754 7.1 |sua1|SPBC28F2.01c|sulfate adenylyltransferase |Schizosaccharomyces pombe|chr 2|||Manual

	Filename	XCorr	DeltCN	ObsM+H+	CalcM+H+	SpR	SpScore	Ion%	#	Sequence	
*	PARC_p629-02.12598.12598.2	3.4488	0.0759	2215.87	2216.205	3	595.4	44.1%	1	R.LS*T#GEVFPIPITLDLNES*.Q	2
*	PARC_p629-02.13325.13325.2	2.8648	0.0194	2502.41	2502.664	33	225.3	30.0%	1	E.WTTVNIKPKDSVSEVT#FAVLS*.Q	2
*	PARC_p629-03.18078.18078.2	2.5175	0.1375	2226.37	2227.18	54	155.7	30.6%	1	K.DS*VSEVTFAVLSQLSDEGY@.L	23

U **SPCC576.10c** 3 3 9.3% 389 43553 5.4 |rpt3||19S proteasome regulatory subunit|Schizosaccharomyces pombe|chr 3|||Manual

	Filename	XCorr	DeltCN	ObsM+H+	CalcM+H+	SpR	SpScore	Ion%	#	Sequence	
*	PARC_p629-04.11594.11594.2	2.6694	0.0229	1941.55	1943.042	16	335.3	39.3%	1	M.NS*TEEIDLYNRWKAL.E	2
*	PARC_p629-04.12655.12655.2	2.7475	0.1073	2285.07	2286.465	20	218.3	31.6%	1	I.IFIDEIDAIATKRFDAQT#GA.D	2
*	PARC_p629-02.13842.13842.2	2.7257	0.179	2286.91	2288.394	20	246.8	31.6%	1	I.FIDEIDAIAT#KRFDAQTGAD.R	2

U **SPAC23D3.13c** 7 10 9.2% 1616 181449 5.9 |||conserved protein|Schizosaccharomyces pombe|chr 1|||Manual

	Filename	XCorr	DeltCN	ObsM+H+	CalcM+H+	SpR	SpScore	Ion%	#	Sequence	
*	PARC_p629-04.12409.12409.2	2.6919	0.086	1607.59	1608.671	6	259.0	50.0%	1	G.SLKFLY@T#NKNLS.Q	2
*	PARC_p629-02.20364.20364.2	2.5552	0.0595	1829.17	1829.885	1	350.4	53.3%	1	P.S*VIGVGS*RVVLADTFE.D	2
*	PARC_p629-02.11227.11227.3	3.6468	0.0555	3174.32	3173.072	44	487.9	21.3%	1	T.T#LSNKAVPS*NLLSGNMTHS*ASSSIS*HNG.R	3
*	PARC_p629-03.13778.13778.2	2.6851	0.1403	1797.59	1798.02	39	265.9	39.3%	1	L.FLRCLSS*IAKVVGEK.M	2
*	PARC_p629-02.18459.18459.3	3.7404	0.0318	4074.98	4074.415	34	378.4	18.8%	2	P.IQTLTAPRQSFNEDLLSANTKNSVYKTSANRS*ISGGI.R	3
*	PARC_p629-04.12288.12288.3	3.7288	0.0549	3433.1	3434.302	134	264.3	21.3%	1	F.ES*LGMLLPVT#EVSDNT#SRGVEYDIS*T#IG.L	3
*	PARC_p629-02.12258.12258.2	2.8923	0.0713	1782.61	1782.866	3	476.1	50.0%	3	I.S*TIGLEALVS*ILETVG.H	2

U **SPAC1B3.16c** 2 3 9.2% 568 62822 8.0 |vht1||vitamin H transporter |Schizosaccharomyces pombe|chr 1|||Manual

	Filename	XCorr	DeltCN	ObsM+H+	CalcM+H+	SpR	SpScore	Ion%	#	Sequence	
*	PARC_p629-03.16815.16815.3	3.5348	0.02	4082.24	4081.349	120	355.6	17.4%	1	I.NPS*FS*GIDIS*LLNAPIWLADALGIVTVMPLY@DRF.H	3
*	PARC_p629-03.11231.11231.2	3.0755	0.0542	1796.45	1795.867	25	456.7	38.2%	2	D.VGVAS*S*LALVTGLGNLGS.V	2

U **SPCC4G3.12c** 4 8 9.1% 821 90500 5.2 |||zinc finger protein|Schizosaccharomyces pombe|chr 3|||Manual

	Filename	XCorr	DeltCN	ObsM+H+	CalcM+H+	SpR	SpScore	Ion%	#	Sequence	
*	PARC_p629-03.20177.20177.3	3.5268	0.0143	3647.87	3646.412	74	466.1	19.8%	1	Q.S*PS*PIPNS*NDNDSQT#RRSSWSSIASAFNDF.P	3
*	PARC_p629-02.12884.12884.2	2.8537	0.0636	2390.15	2390.238	84	222.5	28.9%	1	I.PNS*NDNDSQT#RRSSWSSIAS.A	2

*	PARC_p629-03.16225.16225.3	4.0153	0.0741	4092.41	4092.316	1	630.0	22.1%	1	T.PLNGNDESTVNMLSRLLS*AAAIET#VASIMNS*EARNM.D	3
*	PARC_p629-01.14184.14184.1	2.0951	0.1092	970.51	970.841	3	231.5	56.2%	5	F.DDAS*LSSAD.S	1

U **SPCC16C4.13c** 2 2 9.1% 165 17666 9.3 |rpl1201|rpl12-1, rpl12.1|60S ribosomal protein L12.1/L12A|Schizosaccharomyces pombe|chr 3|||Manual

U **SPCC31H12.04c** 2 2 9.1% 165 17666 9.3 |rpl1202|rpl12-2|60S ribosomal protein L12|Schizosaccharomyces pombe|chr 3|||Manual

Filename	XCorr	DeltCN	ObsM+H+	CalcM+H+	SpR	SpScore	Ion%	#	Sequence	
PARC_p629-04.18724.18724.3	3.6836	0.074	1764.32	1763.788	70	563.8	35.7%	1	G.TVKEILGT#AFSVGCT#.V	3
PARC_p629-04.10089.10089.3	3.6624	0.0916	1764.44	1763.788	60	578.3	37.5%	1	G.TVKEILGT#AFS*VGCT.V	3

U **SPAC20H4.10** 4 5 8.9% 1010 115276 5.9 |ufd2||ubiquitin-protein ligase |Schizosaccharomyces pombe|chr 1|||Manual

	Filename	XCorr	DeltCN	ObsM+H+	CalcM+H+	SpR	SpScore	Ion%	#	Sequence	
*	PARC_p629-02.10760.10760.1	1.8645	0.0245	1758.84	1757.915	5	221.1	36.7%	1	R.SAQNIS*SSISSLKLTM.S	1
*	PARC_p629-03.12946.12946.3	3.5049	0.043	3320.45	3320.452	5	464.4	24.0%	1	L.VQSIRDSEKLKERLETEQQNMS*GS*FQA.T	3
*	PARC_p629-02.14840.14840.2	2.5488	0.0144	2566.63	2567.176	2	286.1	40.6%	1	H.CY@EIMLT#QT#SDTS*RS*FS*.F	2
*	PARC_p629-02.16354.16354.3	3.9133	0.0237	3839.87	3840.359	37	472.9	19.8%	2	N.S*NS*NQNVQES*QS*NLAAAERQAST#Y@CQLGNE.T	3

U **SPAC926.09c** 11 18 8.8% 2073 230558 6.4 |fas1||fatty acid synthase |Schizosaccharomyces pombe|chr 1|||Manual

	Filename	XCorr	DeltCN	ObsM+H+	CalcM+H+	SpR	SpScore	Ion%	#	Sequence	
*	PARC_p629-02.11738.11738.2	3.2067	0.0882	1785.29	1784.85	1	814.7	57.1%	1	V.S*GPARSLYGLNLS*LR.K	2
*	PARC_p629-03.17288.17288.2	2.9242	0.0154	2934.75	2935.227	1	551.2	32.7%	1	G.YPIRGVT#IAAGIPS*LEVANELISTLGV.Q	2
*	PARC_p629-02.12090.12090.2	2.8443	0.1515	2339.07	2339.403	43	221.4	30.0%	1	G.VT#IAAGIPSLEVANELIS*T#LG.V	2
*	PARC_p629-02.16615.16615.2	3.1576	0.2993	1946.29	1945.139	1	713.6	47.2%	1	I.AAGIPS*LEVANELISTLGV.Q	2
*	PARC_p629-02.16223.16223.3	4.6161	0.033	3831.05	3830.868	19	646.7	21.9%	2	A.IRRCDNIVLIAGS*GFGGADDTEPY@LT#GEWSAAF.K	3
*	PARC_p629-02.12158.12158.3	3.9012	0.0682	4008.68	4009.212	1	242.7	20.0%	1	K.GVDDSEWEKTY@DGPTGGIVTVLS*ELGEPIHKLATRG.I	3
*	PARC_p629-03.12123.12123.2	2.5084	0.0114	2471.13	2471.598	141	95.8	28.6%	1	S.EWEKT#YDGPTGGIVTVLSELGE.P	2
*	PARC_p629-03.11368.11368.3	4.4027	0.0496	2213.03	2214.407	7	998.9	36.1%	6	G.IVT#VLS*ELGEPIHKLATRG.I	3
*	PARC_p629-05.08728.08728.3	3.7504	0.0122	3661.34	3660.761	4	656.3	21.7%	1	L.PGTHGIT#HGMY@TS*AAARRFVETYAAQNVPER.V	3
*	PARC_p629-03.15143.15143.2	2.6687	0.0767	2022.85	2024.118	11	347.8	36.7%	1	N.GRKIIKVEVLNQET#S*E.P	2
*	PARC_p629-04.16511.16511.2	2.5407	0.102	2932.53	2931.115	1	280.2	26.1%	2	N.PHS*IT#IHFGGS*KGKKIRDNYMAMA.Y	32

U **SPBC28F2.12** 6 11 8.8% 1752 194161 5.9 |rpb1||DNA-directed RNA polymerase II |Schizosaccharomyces pombe|chr 2|||Manual

	Filename	XCorr	DeltCN	ObsM+H+	CalcM+H+	SpR	SpScore	Ion%	#	Sequence	
*	PARC_p629-02.12144.12144.3	4.7367	0.1504	2240.06	2241.436	1	1989.8	43.4%	3	N.PT#DSGMLIENGEIIYGVVDK.K	3
*	PARC_p629-03.14289.14289.3	4.2821	0.0844	3843.95	3842.933	87	415.7	18.3%	1	N.Y@WLLHNGFSIGIGDTIADADT#MKEVT#RT#VKE.A	3
*	PARC_p629-03.12998.12998.3	3.6335	0.1174	2286.95	2287.427	1	1223.8	40.8%	4	G.EPAT#QMTLNTFHYAGVSSKN.V	3
*	PARC_p629-02.13519.13519.2	3.4	0.1644	2725.55	2726.896	1	571.7	32.7%	1	S.PMVDS*GFVGSPDAAAFSPLVQGGSEGR.E	2
*	PARC_p629-03.17396.17396.3	3.5147	0.0138	4135.73	4136.041	11	348.3	18.1%	1	L.LGAASPY@KGVQSPGY@TSPFS*SAMS*PGYGLTSPSYSPS*.S	3
*	PARC_p629-02.12693.12693.2	2.6184	0.0227	2508.31	2508.037	15	284.7	33.3%	1	P.T#S*PSYSPTSPSY@SATS*PS*Y@.S	2

U **SPCC1393.07c** 4 4 8.8% 845 94518 7.7 |||sequence orphan|Schizosaccharomyces pombe|chr 3|||Manual

	Filename	XCorr	DeltCN	ObsM+H+	CalcM+H+	SpR	SpScore	Ion%	#	Sequence	
*	PARC_p629-03.09420.09420.2	2.5897	0.0658	1540.01	1540.434	1	249.5	60.0%	1	L.PFLT#LSPCS*FT#.I	2
*	PARC_p629-01.10951.10951.2	2.6442	0.0331	1490.23	1489.204	3	788.4	58.3%	1	I.S*SSS*SDPS*SSPPP.S	2
*	PARC_p629-04.19418.19418.2	2.5068	0.0701	1829.23	1828.769	79	188.8	42.9%	1	L.S*VDVKAT#LLETTPPS*.C	2
*	PARC_p629-03.16750.16750.3	3.8183	0.1552	4082.03	4082.355	69	467.7	18.4%	1	E.LILSY@LPEKPIPDGAPIVTNVVETNKTNEAPS*S*FR.K	3

U **SPBC31E1.06** 5 6 8.7% 1121 127645 7.1 ||SPBC800.01|AAA family ATPase|Schizosaccharomyces pombe|chr 2|||Manual

	Filename	XCorr	DeltCN	ObsM+H+	CalcM+H+	SpR	SpScore	Ion%	#	Sequence	
*	PARC_p629-02.13736.13736.2	2.8183	0.0262	1802.51	1802.732	3	322.5	46.9%	1	K.VS*DGSASNNPKAFAVAS*.A	2
*	PARC_p629-03.12747.12747.2	2.7206	0.026	1803.15	1802.732	37	152.3	37.5%	2	K.VS*DGSAS*NNPKAFAVAS.A	2
*	PARC_p629-02.17317.17317.3	4.0495	0.0972	4094.51	4093.239	1	1096.7	22.9%	1	S.NFSKDENS*EAGFGERMVMQLQEAQQPLGVDGNS*GLQ.L	3
*	PARC_p629-02.17380.17380.2	2.7435	0.0145	2731.61	2730.646	14	221.6	25.0%	1	G.ES*AKS*SESDLVVSDDEEDFFKVS.K	2
*	PARC_p629-05.12229.12229.2	2.6028	0.1936	2787.49	2787.743	1	340.9	33.3%	1	K.YTPEHMHCFGT#FY@GPFVAPNS*G.F	2

U **SPBC21C3.01c** 10 10 8.4% 3071 354012 6.3 |vps13a|SPBC31F10.18c|involved in intracellular protein transport |Schizosaccharomyces pombe|chr 2|||Manual

	Filename	XCorr	DeltCN	ObsM+H+	CalcM+H+	SpR	SpScore	Ion%	#	Sequence	
*	PARC_p629-02.14480.14480.3	3.6224	0.0723	3854.39	3855.042	123	490.1	18.5%	1	N.LSDLEKPYSLGLT#LY@SLRVT#STDASFTEYLLS.T	3
*	PARC_p629-03.17584.17584.3	3.6522	0.0309	3823.49	3823.998	28	561.2	20.2%	1	Y.SLRVTSTDASFTEY@LLSTDPIPS*S*CIHKIITV.D	3
*	PARC_p629-02.19228.19228.3	3.8646	0.0207	3823.94	3823.998	14	526.8	19.4%	1	Y.SLRVTST#DASFTEYLLSTDPIPS*S*CIHKIITV.D	3
*	PARC_p629-03.19823.19823.3	3.5411	0.0588	3840.35	3841.304	105	565.0	20.2%	1	L.RAT#AHVVLFRHPTDQIMQLRGKLS*VEEISITL.S	3
*	PARC_p629-02.15479.15479.3	3.5442	0.1183	4122.92	4124.238	1	593.4	21.8%	1	Y.RSRVHT#SLIEERNSIY@LKPKTSAAHGLY@DWFS*.G	3
*	PARC_p629-02.19977.19977.3	3.7645	0.1166	3823.22	3824.156	94	405.1	19.4%	1	Y.ALEMHKT#S*DMTIDLQAPLIVVREECTDLKSPT.L	3
*	PARC_p629-04.19567.19567.2	2.7579	0.1272	2343.69	2343.647	1	504.0	41.7%	1	V.FS*REKLDLVITPQLIETLH.F	2
*	PARC_p629-04.14372.14372.3	3.8364	0.0496	3839.21	3840.663	190	339.6	22.3%	1	M.FSY@S*HNYGSRRCRLRADNSNWS*EPVS*FDA.I	3
*	PARC_p629-	3.7454	0.0237	4172.54	4171.395	4	373.7	19.9%	1	I.LYPS*ILSQEDT#MNDNS*LLPTFHSMVAVVKNDTYGV.T	3

	05.14157.14157.3										
*	PARC_p629-05.14104.14104.3	3.5947	0.296	4076.21	4077.651	85	123.1	19.5%	1	T.NNRILFVQPQQLNCKKEIHLS*KVKTVKLQSKEH.I	3

U **SPAC4A8.11c** 10 16 8.4% 1842 202168 6.2 |lsd1|fas2|fatty acid synthase |Schizosaccharomyces pombe|chr 1|||Manual

	Filename	XCorr	DeltCN	ObsM+H+	CalcM+H+	SpR	SpScore	Ion%	#	Sequence	
*	PARC_p629-02.15447.15447.3	3.9674	0.0147	3840.44	3840.808	187	382.7	17.1%	2	G.SEADAKAWLDSVAQKYAARNGVT#LSS*PT#AEGGSSSG.S	3
*	PARC_p629-02.01910.01910.2	2.522	0.1819	1738.29	1738.436	35	315.4	40.0%	1	S.S*PTAEGGSS*S*GS*AAVI.D	2
*	PARC_p629-02.01644.01644.2	3.442	0.0553	1841.99	1841.755	4	581.8	47.2%	6	S.S*PTAEGGSSSGSAAVIDEE.T	2
*	PARC_p629-02.11730.11730.2	2.5673	0.0117	1983.53	1982.64	108	205.6	32.4%	1	S.S*PTAEGGSS*S*GS*AAVIDE.E	2
*	PARC_p629-05.10784.10784.2	3.1089	0.1658	1999.77	1998.31	7	249.7	40.0%	1	T.FKKLTKNNT#MLVTQQL.E	2
*	PARC_p629-04.13632.13632.2	2.8385	0.0712	2372.81	2373.594	119	107.5	26.3%	1	L.QIKQLYGQVLHALSLPLPS*S*.N	2
*	PARC_p629-02.13495.13495.2	2.504	0.0656	2593.73	2594.8	20	220.7	29.5%	1	G.QVLHALS*LPLPSSNDEQTPVKET.I	2
*	PARC_p629-04.12708.12708.2	2.9159	0.064	1767.59	1766.885	75	274.0	35.3%	1	S.ELGNTVGS*GIGGMSALRG.M	2
*	PARC_p629-03.11248.11248.3	3.9276	0.053	2813.24	2813.999	1	1098.1	33.3%	1	S.KAGT#WTFPATLPAESDISKTNETTR.T	3
*	PARC_p629-02.12450.12450.2	2.5446	0.0714	2252.67	2253.343	1	666.2	42.5%	1	L.TTSLTNSNENVGVDVELVS*AI.S	2

U **SPAC3F10.05c** 2 5 8.3% 326 37867 7.0 |||sequence orphan|Schizosaccharomyces pombe|chr 1|||Manual

	Filename	XCorr	DeltCN	ObsM+H+	CalcM+H+	SpR	SpScore	Ion%	#	Sequence	
*	PARC_p629-04.11577.11577.2	3.0951	0.0276	1803.55	1802.813	3	435.9	46.4%	4	S.S*TNLGFSNSLQDKWD.C	2
*	PARC_p629-05.09297.09297.2	2.5791	0.0216	1787.91	1789.014	6	623.6	59.1%	1	R.FY@RWLSRSLKWR.P	2

U **SPAC1D4.04** 3 3 8.2% 527 56677 5.7 |cct2||T-complex protein 1 |Schizosaccharomyces pombe|chr 1|||Manual

	Filename	XCorr	DeltCN	ObsM+H+	CalcM+H+	SpR	SpScore	Ion%	#	Sequence	
*	PARC_p629-02.11947.11947.2	3.0592	0.0754	2084.11	2084.181	23	496.5	38.9%	1	G.MDKILQS*NSSGDIVVTNDG.A	2
*	PARC_p629-02.19015.19015.2	2.5753	0.1253	2549.39	2548.772	34	188.7	25.0%	1	S.SGDIVVTNDGAT#ILKSIALDNAAAK.V	32
*	PARC_p629-02.06580.06580.2	2.5159	0.0665	1184.71	1185.116	23	367.0	56.2%	1	L.ENIART#T#LS.S	2

U **SPBC13E7.08c** 2 3 8.2% 429 48733 4.5 |||proteasome interacting protein |Schizosaccharomyces pombe|chr 2|||Manual

	Filename	XCorr	DeltCN	ObsM+H+	CalcM+H+	SpR	SpScore	Ion%	#	Sequence	
*	PARC_p629-02.19284.19284.3	4.0626	0.0273	3838.01	3838.992	136	432.7	17.6%	1	S.ET#AVSDDGLFS*NTEEATEAPEADVPVKKVLEVAVP.N	3
*	PARC_p629-02.19152.19152.3	3.8578	0.0258	3838.13	3838.992	46	502.3	19.1%	2	S.ETAVS*DDGLFSNT#EEATEAPEADVPVKKVLEVAVP.N	3

U **SPAPYUG7.03c** 3 4 8.1% 704 78717 5.5 |mid2||anillin homologue|Schizosaccharomyces pombe|chr 1|||Manual

	Filename	XCorr	DeltCN	ObsM+H+	CalcM+H+	SpR	SpScore	Ion%	#	Sequence	
*	PARC_p629-04.08612.08612.2	2.7569	0.0408	1769.71	1768.581	2	617.4	50.0%	1	A.EDGDSFPYDQT#LS*N.S	2
*	PARC_p629-04.19800.19800.3	3.9821	0.0622	3862.67	3864.019	1	639.6	22.0%	1	S.VMNETDSSSTSVEDVNKENILSLNDS*CLIKLSDD.E	3
*	PARC_p629-01.13535.13535.1	1.93	0.0118	1314.63	1316.117	22	377.0	56.2%	2	D.NHY@S*DEEEL.E	1

U **SPCC4B3.04c** 6 8 8.0% 1316 147846 8.0 |||cyclic nucleic acid binding family protein|Schizosaccharomyces pombe|chr 3|||Manual

	Filename	XCorr	DeltCN	ObsM+H+	CalcM+H+	SpR	SpScore	Ion%	#	Sequence	
*	PARC_p629-02.14511.14511.3	3.6732	0.0142	3528.74	3529.483	26	152.1	18.3%	1	P.NSS*AQIVQVILT#RFQRVTFS*TGY@EY@LG.L	3
*	PARC_p629-02.13923.13923.2	2.58	0.0861	2286.03	2287.346	62	177.4	27.8%	1	E.AMS*EHLSQASNESFIVLRS*.K	2
*	PARC_p629-04.16020.16020.2	2.5564	0.1112	2441.89	2440.672	12	242.3	27.5%	1	A.TDREEVKSSIVVS*TKKSTILE.F	2
*	PARC_p629-04.11359.11359.2	2.6703	0.0728	1342.13	1343.404	2	893.4	65.0%	3	G.FLDATCPSKLT#.F	2
*	PARC_p629-02.12193.12193.2	2.5166	0.0629	1781.45	1782.703	40	243.6	46.2%	1	Q.S*RLTY@VT#SVATGEK.V	2
*	PARC_p629-04.09012.09012.2	2.5106	0.0628	1739.49	1739.768	2	248.2	45.8%	1	P.RDTSKRHPKFLS*S*.R	2

U **SPBC530.14c** 2 2 7.9% 544 61056 8.1 |dsk1||serine/threonine protein kinase|Schizosaccharomyces pombe|chr 2|||Manual

	Filename	XCorr	DeltCN	ObsM+H+	CalcM+H+	SpR	SpScore	Ion%	#	Sequence	
*	PARC_p629-03.11658.11658.2	2.6638	0.0118	2679.13	2680.514	2	320.5	30.4%	1	H.IEAPATT#S*SPTSNTSSSKT#RNNTG.Y	2
*	PARC_p629-02.12227.12227.3	4.2292	0.0946	2214.29	2214.065	1	1393.7	40.3%	1	N.SHPNS*PFS*SGDNS*LILDGV.N	3

U **SPAC19D5.04** 11 12 7.8% 3227 365036 5.1 |ptr1||HECT domain|Schizosaccharomyces pombe|chr 1|||Manual

	Filename	XCorr	DeltCN	ObsM+H+	CalcM+H+	SpR	SpScore	Ion%	#	Sequence	
*	PARC_p629-05.11879.11879.3	3.5468	0.0316	4173.98	4175.284	116	285.7	18.8%	1	S.FS*AHLLSHCANRSIY@NSTVYLEY@LLNS*SVLEVI.D	3
*	PARC_p629-02.12456.12456.3	3.6705	0.0502	2218.61	2217.315	2	1044.9	38.2%	1	S.PS*KDFSSASQPNSEGFSILK.L	3
*	PARC_p629-02.20735.20735.3	3.5137	0.048	3839.96	3841.22	195	439.6	18.2%	1	M.ACSAGLTSLLGYHLSVKT#PQATYVVARS*IVMLDH.L	3
*	PARC_p629-05.12245.12245.2	2.5854	0.422	2242.45	2243.426	3	198.7	30.6%	1	G.YSMAFPDFSES*KGLDMLVD.R	2
*	PARC_p629-02.09524.09524.2	3.1519	0.1131	1334.39	1333.408	1	949.4	70.0%	1	K.LS*HAFLDAVNR.K	2
*	PARC_p629-03.15852.15852.3	3.556	0.0542	3375.26	3375.81	18	384.6	22.3%	1	L.NLILVFT#SADFMSETSKTLNMALKSEFDM.T	3
*	PARC_p629-03.18078.18078.3	3.8598	0.0404	3339.05	3338.66	24	306.7	20.7%	2	I.GSYQEPSLINIRGREVDVS*SLGIDPTFLLA.L	23
*	PARC_p629-05.14559.14559.3	3.6134	0.2664	3339.59	3338.66	207	312.3	19.0%	1	I.GSYQEPS*LINIRGREVDVSSLGIDPTFLLA.L	3
*	PARC_p629-03.16681.16681.3	3.8878	0.0224	4083.17	4082.752	114	397.0	18.8%	1	Y.KS*EKLQKDSAT#TGYT#NDQDSRGS*T#VPKQDPGTT#.A	3
*	p629-05ms3.10420.10420.3	3.5532	0.1778	3160.1	3161.466	61	370.9	22.1%	1	W.MLNNDIT#DIITEEFAVEKDVFGEKTVV.D	3
*	PARC_p629-05.09368.09368.2	2.6222	0.0255	1787.71	1788.908	40	358.5	39.3%	1	N.EQELELLIS*GLPEID.I	2

U **SPBC887.04c** 2 2 7.7% 713 78654 5.3 |lub1||WD repeat protein|Schizosaccharomyces pombe|chr 2|||Manual

	Filename	XCorr	DeltCN	ObsM+H+	CalcM+H+	SpR	SpScore	Ion%	#	Sequence	
*	PARC_p629-02.09013.09013.3	3.5323	0.1894	2983.43	2984.13	1	478.4	27.1%	1	Q.GHS*LES*KKEPNLESQSSNKIKTTIF.P	23
*	PARC_p629-05.09513.09513.3	3.7528	0.0562	3429.8	3430.613	5	447.2	24.1%	1	V.KSVPS*SGNFESINVMLALRGLS*NVVPNIT#D.A	3

U **SPAC1F3.06c** 7 7 7.6% 1957 222785 5.2 |spo15||coiled-coil |Schizosaccharomyces pombe|chr 1|||Manual

	Filename	XCorr	DeltCN	ObsM+H+	CalcM+H+	SpR	SpScore	Ion%	#	Sequence	

*	PARC_p629-02.06586.06586.3	3.5837	0.1851	1745.96	1744.611	20	590.4	42.9%	1	S.S*EGS*T#ASSALPLTPR.S	3
*	PARC_p629-02.09787.09787.2	2.84	0.1444	2231.57	2232.327	182	105.8	33.3%	1	Q.LNELKGELQT#EISNSEHLS.S	2
*	PARC_p629-03.13051.13051.2	2.5084	0.0991	1825.55	1825.845	10	455.5	43.3%	1	A.VAT#NNELSESKNSLQT.L	2
*	PARC_p629-02.16549.16549.3	3.5798	0.054	3837.41	3838.765	22	560.7	21.0%	1	E.QSNSNNEALVEERS*DLANRLS*DMKKSLS*DSDN.V	3
*	PARC_p629-03.12268.12268.2	2.5162	0.042	1838.53	1838.989	1	721.3	53.6%	1	L.S*LMENIKSQLQEAKE.K	2
*	PARC_p629-04.16428.16428.3	3.663	0.0697	4088.09	4088.143	25	430.4	18.0%	1	S.QLQEAKEKIQVDEST#IQELDHEITAS*KNNY@EGK.L	3
*	PARC_p629-02.12353.12353.3	3.51	0.0526	3098.15	3099.262	1	840.4	29.2%	1	I.VKFKEDLEKCAAEEKEKQAT#FDNYS.E	3

U **SPBC2F12.05c** 5 6 7.4% 1310 148475 7.3 |||involved in ergosterol biosynthesis |Schizosaccharomyces pombe|chr 2|||Manual

	Filename	XCorr	DeltCN	ObsM+H+	CalcM+H+	SpR	SpScore	Ion%	#	Sequence	
*	PARC_p629-04.16968.16968.2	2.5659	0.056	2786.89	2785.888	108	102.8	21.7%	1	Y.SGNRDSSS*APAVPQHMS*GYLKKWT.N	2
*	PARC_p629-02.17901.17901.1	2.0312	0.1594	1755.64	1755.846	36	98.2	34.6%	1	L.PS*RENFEFNVNIAK.L	1
*	PARC_p629-03.17982.17982.3	3.7759	0.0558	3856.4	3856.813	1	564.0	21.0%	1	S.S*ES*KQSTQHQQPVEVQKSIQS*DVS*APKAKEVS.E	3
*	PARC_p629-02.12586.12586.2	3.2476	0.0914	2251.81	2252.285	7	641.4	40.6%	2	P.T#RENLDVNDDRPKIS*LW.G	2
*	PARC_p629-02.07766.07766.1	2.0574	0.1777	1099.69	1099.061	2	249.1	50.0%	1	I.LGSPS*VDNYG.Q	1

U **SPAC22A12.15c** 3 3 7.4% 663 73227 5.0 |bip1|bip|BiP |Schizosaccharomyces pombe|chr 1|||Manual

	Filename	XCorr	DeltCN	ObsM+H+	CalcM+H+	SpR	SpScore	Ion%	#	Sequence	
*	PARC_p629-04.09848.09848.1	1.9351	0.0744	1339.72	1340.337	32	118.6	55.6%	1	G.T#TY@SCVAVMK.N	1
*	PARC_p629-02.07883.07883.2	2.6641	0.0337	2262.83	2264.332	1	614.5	42.1%	1	A.FTEDERLVGEAAKNQAPS*NP.E	2
*	PARC_p629-04.11461.11461.2	2.844	0.0108	2211.73	2212.302	1	1144.5	52.8%	1	D.S*NLKKSEIDDIVLVGGST#R.I	2

U **SPBPB7E8.01** 3 3 7.4% 569 61400 4.9 |||glycoprotein |Schizosaccharomyces pombe|chr 2|||Manual

	Filename	XCorr	DeltCN	ObsM+H+	CalcM+H+	SpR	SpScore	Ion%	#	Sequence	
*	PARC_p629-02.18465.18465.3	3.8514	0.0303	4061.81	4060.518	26	467.9	20.1%	1	E.T#S*ILDSTNTTSTNATNTTT#TT#S*SSS*TASSSASASSST.S	3
*	PARC_p629-03.15135.15135.3	3.5918	0.1132	4183.85	4182.805	80	179.3	17.5%	1	T.SILDSTNTTSTNATNTTTTTSSSST#AS*SSASASSSTSAT#S*G.A	3
*	PARC_p629-01.10131.10131.1	2.0086	0.0402	1141.66	1141.833	36	93.8	61.1%	1	T.SS*S*STASS*SA.S	1

U **SPAC27E2.09** 9 17 7.3% 2310 264620 7.4 |mak2||histidine kinase |Schizosaccharomyces pombe|chr 1|||Manual

	Filename	XCorr	DeltCN	ObsM+H+	CalcM+H+	SpR	SpScore	Ion%	#	Sequence	
*	PARC_p629-03.14048.14048.3	3.624	0.023	3250.91	3250.446	1	582.7	26.0%	1	I.QKMTAKSMNSRIS*S*ATDLCYT#IVELM.Q	3
*	PARC_p629-02.11340.11340.2	2.5019	0.0422	1850.39	1849.999	23	250.9	42.9%	1	S.SSNVYDYLPAFRLLT#.K	2
*	PARC_p629-03.11748.11748.2	2.9748	0.033	1801.49	1802.021	17	394.0	42.9%	4	G.S*AVTLESILLQCVMF.G	2
*	PARC_p629-03.18769.18769.3	3.5298	0.0837	3822.8	3823.823	12	434.5	19.2%	1	A.TTTGDYAEKLKLLRNQDINDFS*LGLAS*Y@S*DI.F	3
*	PARC_p629-01.16652.16652.2	3.0974	0.0266	2102.71	2102.187	12	252.1	41.2%	6	R.NDEESFDIVSLVS*VIKCG.Q	2
*	PARC_p629-04.13712.13712.2	2.5414	0.1068	2368.67	2369.569	73	184.8	27.8%	1	L.SRRAIRT#GNVT#FLKLLSQQ.I	2
*	PARC_p629-02.08710.08710.1	2.0892	0.1348	1253.02	1253.418	2	490.8	61.1%	1	S.ELKS*GKMKLE.P	1
*	PARC_p629-02.12634.12634.2	3.1398	0.0363	2254.93	2253.51	72	409.7	36.8%	1	S.GLGLS*ICLQICKIMDGDIGV.Q	2
*	PARC_p629-04.10177.10177.2	2.5307	0.0976	1780.49	1780.897	8	224.4	46.4%	1	D.GDPFITS*LNKNQSRI.F	2

U **SPAC26F1.13c** 3 4 7.3% 1111 126451 7.3 ||||leucine-tRNA ligase |Schizosaccharomyces pombe|chr 1|||Manual

	Filename	XCorr	DeltCN	ObsM+H+	CalcM+H+	SpR	SpScore	Ion%	#	Sequence	
*	PARC_p629-05.11143.11143.3	3.5434	0.0391	3838.82	3839.825	27	456.1	19.8%	1	M.AT#T#EPS*VEQLET#KTAKLKLENT#TKRDTLIE.L	3
*	PARC_p629-02.13059.13059.2	2.8482	0.0567	2485.87	2487.321	1	252.3	36.1%	2	V.S*S*PVNTALSNSLLY@IRT#LS*.R	2
*	PARC_p629-02.15246.15246.3	3.7802	0.0874	3854.93	3856.377	57	482.7	19.4%	1	K.KEMKRAMPFIQQFKQSVINRGEHVSANSIFS*R.E	3

U **SPBC146.03c** 4 8 7.2% 1324 150652 6.5 |cut3|smc4|condensin subunit|Schizosaccharomyces pombe|chr 2|||Manual

	Filename	XCorr	DeltCN	ObsM+H+	CalcM+H+	SpR	SpScore	Ion%	#	Sequence	
*	PARC_p629-03.11347.11347.2	3.6668	0.0289	2216.65	2215.436	1	936.7	47.1%	3	G.ERPRKLVRSVLESPS*QKD.V	2
*	PARC_p629-02.16858.16858.3	3.6097	0.0235	4109.84	4108.877	53	424.8	19.2%	1	G.FNERS*S*QLVS*EFT#TTEDIENCEET#TQVLPPR.L	3
*	PARC_p629-02.18204.18204.3	3.7195	0.0452	4062.47	4062.938	99	478.3	18.5%	1	A.S*ALIHKSAT#HPS*LDSCDVEIT#FKEVNS*DFTYV.D	3
*	PARC_p629-04.11495.11495.2	2.6575	0.0247	1769.67	1769.867	3	511.0	50.0%	3	H.LS*ELESLNQRFTEI.S	2

U **SPAC12G12.04** 3 3 7.2% 582 62168 6.0 |mcp60|hsp60|heat shock protein 60|Schizosaccharomyces pombe|chr 1|||Manual

	Filename	XCorr	DeltCN	ObsM+H+	CalcM+H+	SpR	SpScore	Ion%	#	Sequence	
*	PARC_p629-04.16108.16108.3	4.0295	0.0358	3376.61	3377.491	1	533.1	22.4%	1	G.ARLVQDVASKTNEVAGDGTT#TAT#VLT#RAIF.S	3
*	PARC_p629-02.01783.01783.2	2.6932	0.074	1798.85	1797.835	18	455.1	43.8%	1	K.T#NEVAGDGTTTATVLTR.A	2
*	PARC_p629-03.09163.09163.2	2.7319	0.0219	1323.25	1324.305	3	549.8	59.1%	1	I.KVGGS*SEVEVNE.K	2

U **SPAPYUK71.03c** 4 4 7.1% 1225 135781 7.2 |||C2 domain|Schizosaccharomyces pombe|chr 1|||Manual

	Filename	XCorr	DeltCN	ObsM+H+	CalcM+H+	SpR	SpScore	Ion%	#	Sequence	
*	PARC_p629-05.09351.09351.2	2.5228	0.0549	1791.83	1790.93	6	494.3	46.9%	1	V.T#PAPKASSTVATDKVNI.E	2
*	PARC_p629-03.15851.15851.2	2.5689	0.3342	2316.37	2315.509	1	180.9	27.5%	1	I.FLDDLPHHIFLSSALNVT#GGA.T	2
*	PARC_p629-02.12344.12344.1	2.2318	0.0451	1798.76	1798.932	91	83.4	28.6%	1	K.T#MLSRDLISKGAT#KP.L	1
*	PARC_p629-03.14916.14916.3	3.6133	0.0121	4161.74	4161.509	127	172.3	16.7%	1	T.NYEIPLDSKQGVLY@LRIT#LSPKWVLRSKRAGNSS*.L	3

U **SPAC1783.05** 7 21 7.0% 1373 158548 5.8 |hrp1|chd1|ATPase|Schizosaccharomyces pombe|chr 1|||Manual

	Filename	XCorr	DeltCN	ObsM+H+	CalcM+H+	SpR	SpScore	Ion%	#	Sequence	
*	PARC_p629-03.11629.11629.2	3.0916	0.0981	1772.25	1770.771	1	645.2	46.7%	7	T.ANS*TVVSSALST#PKID.D	2
*	p629-02ms3.11997.11997.2	2.5775	0.0648	2315.95	2317.439	57	92.5	31.6%	1	S.GTEIRTSLRSSKGKSVNY@NE.Q	2
*	PARC_p629-02.09328.09328.1	1.9136	0.0376	1475.84	1476.417	36	137.7	40.0%	1	R.FLS*KDT#VEEDI.L	1
*	PARC_p629-01.15435.15435.2	2.5526	0.0453	2045.83	2045.007	136	132.8	29.4%	3	K.APT#KS*STRQTTLDGSISN.T	2
*	PARC_p629-03.10171.10171.2	2.5759	0.0146	1340.61	1340.215	4	784.4	68.2%	1	D.ETS*PEGTVGEDE.V	2
*	PARC_p629-02.09849.09849.1	2.1529	0.0446	1064.98	1064.099	2	160.3	55.6%	2	T.LT#AVAALDAE.L	1
*	PARC_p629-03.07839.07839.2	2.666	0.2387	1156.31	1155.076	5	491.0	68.8%	6	T.EDLSLET#EE.N	2

U **SPAC6F12.10c** 4 4 6.9% 1323 144866 5.7 |ade3|min11|phosphoribosylformylglycinamidine synthase |Schizosaccharomyces pombe|chr 1|||Manual

	Filename	XCorr	DeltCN	ObsM+H+	CalcM+H+	SpR	SpScore	Ion%	#	Sequence	
*	PARC_p629-02.15158.15158.3	4.1295	0.0163	3860.06	3859.003	3	517.2	20.7%	1	L.S*VQS*KKKILELTVSGVSDLNAVY@FY@LIYT#K.S	3
*	PARC_p629-04.13546.13546.2	2.7505	0.1037	2802.13	2800.877	3	215.8	25.0%	1	N.HPTAVSPFPGAAT#GSGGEIRDEGAVGQGS.L	32
*	PARC_p629-05.08214.08214.2	2.9185	0.146	1393.87	1392.485	7	355.3	54.5%	1	F.T#AVDVHMTDILS.G	2
*	PARC_p629-05.10111.10111.2	3.2341	0.1098	2345.17	2346.361	5	325.7	34.2%	1	R.T#S*RYPFNPNGSRDAIAGVRS.P	2

U **SPCC330.02** 2 2 6.7% 563 62728 6.8 ||SPCC613.14|leucine-rich repeat protein |Schizosaccharomyces pombe|chr 3|||Manual

	Filename	XCorr	DeltCN	ObsM+H+	CalcM+H+	SpR	SpScore	Ion%	#	Sequence	
*	PARC_p629-02.17636.17636.2	2.8566	0.0143	2214.79	2215.227	4	430.3	46.9%	1	F.TIT#PYSKY@SNSEKGWLC.Y	2
*	PARC_p629-02.14498.14498.2	2.6109	0.0418	2234.53	2233.32	18	159.7	30.0%	1	S.PGGIINDGS*ILDVLNQIGS*GL.H	2

U **SPAC1565.08** 3 4 6.6% 815 90125 5.0 |cdc48|SPAC6F12.01|AAA family ATPase|Schizosaccharomyces pombe|chr 1|||Manual

	Filename	XCorr	DeltCN	ObsM+H+	CalcM+H+	SpR	SpScore	Ion%	#	Sequence	
*	PARC_p629-03.11583.11583.2	2.5627	0.0125	2679.23	2678.632	5	242.9	29.5%	1	V.DDAT#NDDNSVIT#LSSNTMETLQL.F	2
*	PARC_p629-05.08048.08048.1	1.8675	0.1627	1321.68	1322.252	2	154.3	41.7%	1	S.AGDS*GGGDRVVNQ.L	1
*	PARC_p629-03.11521.11521.2	3.7109	0.1468	2240.81	2241.167	4	747.3	44.1%	2	I.KDS*IEEDIKRENET#GEAP.A	2

U **SPBC21H7.04** 3 3 6.6% 709 78831 8.9 |||DEAD/DEAH box helicase|Schizosaccharomyces pombe|chr 2|||Manual

	Filename	XCorr	DeltCN	ObsM+H+	CalcM+H+	SpR	SpScore	Ion%	#	Sequence	
*	PARC_p629-02.01897.01897.2	2.672	0.0604	2118.43	2117.325	50	169.0	33.3%	1	D.KDAFIEAQT#GSGKTLAYLL.P	2
*	PARC_p629-02.20870.20870.3	3.536	0.0727	3377.27	3376.294	7	504.1	23.1%	1	R.LHGSLS*QQIRT#STLNLFSSSEDS*GS*HIL.L	3
*	PARC_p629-03.12836.12836.2	2.6121	0.0221	2230.95	2232.205	10	345.4	38.9%	1	Q.IRTSTLNLFSSS*EDS*GSHI.L	32

U **SPAC222.10c** 2 2 6.6% 665 75694 5.6 |byr4||two-component GAP for GTPase spg1 |Schizosaccharomyces pombe|chr 1|||Manual

	Filename	XCorr	DeltCN	ObsM+H+	CalcM+H+	SpR	SpScore	Ion%	#	Sequence	
*	PARC_p629-02.14677.14677.3	3.5833	0.0276	3254.81	3255.429	223	237.0	19.2%	1	I.LHRKQNRMDPKASFSSVEQS*S*LRTPSS.A	3
*	PARC_p629-04.10944.10944.2	2.5697	0.0157	2077.39	2077.013	10	170.5	37.5%	1	P.VKGNS*SISST#WT#PSNLK.I	32

U **SPAC29A4.08c** 3 4 6.6% 488 54191 5.1 |prp19|cwf8|ubiquitin-protein ligase |Schizosaccharomyces pombe|chr 1|||Manual

	Filename	XCorr	DeltCN	ObsM+H+	CalcM+H+	SpR	SpScore	Ion%	#	Sequence	
*	PARC_p629-02.14822.14822.3	4.7845	0.0525	3854.51	3855.951	32	551.6	21.0%	1	K.FGPHS*S*PVKTLQFGENGY@WLVVTTNDDSDIFI.W	3
*	PARC_p629-04.12666.12666.3	3.514	0.0164	3854.57	3855.951	167	360.6	20.2%	1	K.FGPHS*SPVKT#LQFGENGY@WLVVTTNDDSDIFI.W	3
*	PARC_p629-05.09815.09815.3	3.8333	0.0734	3856.07	3855.951	245	359.2	19.4%	2	K.FGPHS*S*PVKTLQFGENGYWLVVT#TNDDSDIFI.W	3

U **SPCC10H11.01** 3 3 6.4% 1014 114213 7.9 |prp11||DEAD/DEAH box helicase|Schizosaccharomyces pombe|chr 3|||Manual

	Filename	XCorr	DeltCN	ObsM+H+	CalcM+H+	SpR	SpScore	Ion%	#	Sequence	
*	PARC_p629-01.18141.18141.2	2.5418	0.0676	2043.13	2043.177	153	166.7	29.4%	1	N.AMPSTGIS*GFEINRQKDT.S	2
*	PARC_p629-02.11930.11930.1	2.1071	0.129	1479.17	1480.666	3	264.2	37.5%	1	G.VFDVLIAT#SVVAR.G	1
*	PARC_p629-03.17971.17971.3	3.695	0.0412	3824.27	3825.284	20	513.9	21.2%	1	E.LVVNRAITELRRLLLEGINHSLEGGNKPSAS*GRY.T	3

U **SPBC16G5.16** 3 3 6.4% 827 94566 7.3 |||transcriptional regulator |Schizosaccharomyces pombe|chr 2|||Manual

	Filename	XCorr	DeltCN	ObsM+H+	CalcM+H+	SpR	SpScore	Ion%	#	Sequence	
*	PARC_p629-02.12886.12886.2	3.388	0.0423	2595.65	2594.802	75	132.9	26.2%	1	S.QPDPLS*FPYLPPTPAEDEHKKP.P	32
*	PARC_p629-02.12063.12063.2	2.6094	0.0115	1795.39	1795.787	1	818.4	53.8%	1	F.S*DVLT#SHESRLSKR.V	2
*	PARC_p629-01.10961.10961.2	2.7247	0.0793	2143.35	2143.082	10	259.5	40.6%	1	V.YPVKSPS*DS*HCKVS*LPA.V	2

U **SPAC23D3.06c** 5 12 6.0% 1325 145776 5.1 |nup146||nucleoporin|Schizosaccharomyces pombe|chr 1|||Manual

	Filename	XCorr	DeltCN	ObsM+H+	CalcM+H+	SpR	SpScore	Ion%	#	Sequence	
*	PARC_p629-04.14988.14988.2	3.4825	0.0843	2340.65	2339.26	26	354.2	35.0%	4	N.T#NEPNAGGSQVTESEIEEEDI.E	2
*	PARC_p629-05.12618.12618.3	3.5421	0.0266	3472.31	3473.551	1	932.9	26.8%	1	T.KRAS*LPYSNKKDSDSSPTALVMDFT#AT#DR.I	3
*	PARC_p629-01.16765.16765.2	2.8659	0.0314	1860.87	1859.86	3	240.1	46.9%	4	E.YIEGQTST#PSAVSEQAT.M	2
*	PARC_p629-02.01903.01903.2	3.1407	0.0224	1861.33	1859.86	1	279.2	53.1%	2	E.YIEGQTSTPSAVS*EQAT.M	2
*	PARC_p629-02.07711.07711.2	2.5547	0.1342	1464.37	1464.621	1	227.0	50.0%	1	E.KVLKS*LSTLEQQ.A	2

U **SPBC1105.04c** 2 2 5.9% 522 59840 6.0 |cbp1|abp1|ARS binding protein|Schizosaccharomyces pombe|chr 2|||Manual

	Filename	XCorr	DeltCN	ObsM+H+	CalcM+H+	SpR	SpScore	Ion%	#	Sequence	
*	PARC_p629-02.12528.12528.2	2.6643	0.1247	2212.17	2212.34	1	653.9	41.7%	1	H.ILHAINEQPTESVVLNNT#E.P	2
*	PARC_p629-02.08568.08568.2	2.5601	0.0818	1526.49	1525.704	1	818.5	59.1%	1	I.T#ISLLNYQKLLE.A	2

U **SPAC17G6.13** 2 2 5.8% 433 49137 5.0 |slt1||involved in response to drug|Schizosaccharomyces pombe|chr 1|||Manual

	Filename	XCorr	DeltCN	ObsM+H+	CalcM+H+	SpR	SpScore	Ion%	#	Sequence	
*	PARC_p629-05.09171.09171.3	3.6582	0.0454	3212.66	3211.494	32	224.5	26.1%	1	I.Y@S*PEES*S*ESQAEVADQQT#GPY@S*T#.S	3
*	PARC_p629-04.14970.14970.2	2.8939	0.1186	2337.45	2338.814	9	314.3	34.4%	1	E.S*QAEVADQQT#GPY@S*T#S*E.Y	2

U **SPBP19A11.04c** 7 13 5.7% 2196 251196 5.9 |mor2|cps12|HEAT repeat |Schizosaccharomyces pombe|chr 2|||Manual

	Filename	XCorr	DeltCN	ObsM+H+	CalcM+H+	SpR	SpScore	Ion%	#	Sequence	
*	PARC_p629-02.07189.07189.1	2.0054	0.1932	1152.57	1154.056	8	300.9	56.2%	7	E.T#IPS*NALEE.S	1
*	PARC_p629-02.09704.09704.2	2.8002	0.1353	1828.81	1829.018	1	630.9	53.6%	1	Q.LYPTT#LLEESIAFLQ.S	2
*	PARC_p629-03.12154.12154.2	3.2286	0.0232	2593.75	2593.681	9	209.3	34.2%	1	I.IHT#VCHNSLIYFS*SGLKMS*K.S	2
*	PARC_p629-02.13414.13414.3	3.7273	0.107	3345.89	3346.438	64	432.5	23.1%	1	R.IWIS*QQEAVIEKRNSVLS*NKSCASSESN.T	3
*	PARC_p629-04.11666.11666.2	2.72	0.1012	1770.51	1769.911	5	477.4	50.0%	1	R.IES*SELNFNLKSAVK.N	2
*	PARC_p629-03.12785.12785.3	3.6703	0.0642	3319.91	3319.202	122	317.8	20.8%	1	P.QYLFSVQLAS*DFT#EDS*FT#LTSECLR.Y	3
*	PARC_p629-04.12245.12245.2	2.5057	0.0158	1801.55	1800.81	27	218.2	45.8%	1	I.QFDHYS*RIVQEQC.Q	2

U **SPBC1826.01c** 6 6 5.7% 1953 217630 6.3 |||HEAT repeat |Schizosaccharomyces pombe|chr 2|||Manual

	Filename	XCorr	DeltCN	ObsM+H+	CalcM+H+	SpR	SpScore	Ion%	#	Sequence	
*	PARC_p629-05.10904.10904.3	3.5395	0.0315	2876.81	2876.806	223	291.8	25.0%	1	G.SPT#TS*IPEHKTSINNNKPEDT#PTP.S	3
*	PARC_p629-02.18955.18955.3	3.6594	0.0577	3854.12	3855.296	149	442.4	18.3%	1	F.HLMRYTLTGVRRS*VVYALTKFISVQTSCS*WI.T	3
*	PARC_p629-01.18467.18467.1	2.0043	0.1051	1093.42	1094.189	1	275.5	56.2%	1	G.AQLT#LQQMA.Q	1
*	PARC_p629-02.12853.12853.2	3.0137	0.0113	2679.29	2679.495	37	144.9	23.9%	1	L.RDCGLGNS*SVNS*NGIDSALT#NAVS.E	2
*	PARC_p629-02.08253.08253.1	2.0342	0.0183	1267.52	1266.269	5	220.2	50.0%	1	Q.NAGLSS*IGTDQI.L	1
*	PARC_p629-02.12169.12169.2	2.5516	0.1642	1815.65	1814.819	7	160.7	42.9%	1	G.T#DQILDLFNTTADEQ.Q	2

U **SPBC1604.21c** 3 3 5.7% 1012 112949 5.2 |ptr3|uba1, SPBC211.09|ubiquitin activating enzyme |Schizosaccharomyces pombe|chr 2|||Manual

	Filename	XCorr	DeltCN	ObsM+H+	CalcM+H+	SpR	SpScore	Ion%	#	Sequence	
*	PARC_p629-04.14628.14628.2	2.5538	0.1159	2502.51	2501.156	1	361.7	38.2%	1	A.S*T#AVS*IMNPS*LTGKIT#S*Y@.Q	2
*	PARC_p629-02.12312.12312.2	2.6235	0.0577	2030.35	2030.166	14	496.8	43.3%	1	F.FNNNIQQLLFNFPKDS*.V	2
*	PARC_p629-02.12628.12628.2	2.5729	0.0892	2678.77	2679.861	17	144.2	26.1%	1	I.ADSLPPPS*SLVGFRLTPAEFEKDD.D	2

U **SPCC364.02c** 2 2 5.7% 384 43023 9.2 |bis1||stress response protein bis1|Schizosaccharomyces pombe|chr 3|||Manual

	Filename	XCorr	DeltCN	ObsM+H+	CalcM+H+	SpR	SpScore	Ion%	#	Sequence	
*	PARC_p629-02.10600.10600.1	1.9361	0.0474	1077.74	1077.96	37	211.5	57.1%	1	G.Y@S*NSDAIK.S	1
*	PARC_p629-02.10458.10458.2	2.535	0.0104	1608.57	1608.548	8	358.2	46.2%	1	H.GSS*PS*ASRHTALRT.S	2

U **SPCC737.08** 12 15 5.5% 4717 537794 5.2 |||midasin |Schizosaccharomyces pombe|chr 3|||Manual

	Filename	XCorr	DeltCN	ObsM+H+	CalcM+H+	SpR	SpScore	Ion%	#	Sequence	
*	PARC_p629-03.08447.08447.2	2.9667	0.0872	1580.53	1581.682	2	573.5	50.0%	3	G.ETGTGKT#TTIQLLAG.L	2
*	PARC_p629-05.10608.10608.3	3.6503	0.0351	4186.79	4187.09	2	328.5	20.7%	1	R.PHY@T#VRTLSRTLSY@VT#EIAPIY@GLRRS*LY@E.G	3
*	PARC_p629-05.08586.08586.2	2.7121	0.0314	1796.49	1795.87	7	577.8	50.0%	2	D.S*VLERLNS*VLELSR.T	2
*	PARC_p629-	2.6202	0.0116	2274.51	2275.485	1	449.1	38.9%	1	K.THS*QAFTLLQKVVDQLDQL.K	2

	03.09597.09597.2										
*	PARC_p629-05.15449.15449.2	2.5168	0.092	1889.79	1890.886	2	422.7	42.9%	1	V.Y@HNVSSFKGSTPS*LW.N	2
*	PARC_p629-03.19954.19954.3	4.0266	0.015	3648.98	3647.908	69	437.0	19.6%	1	P.SLWNLLNQFSKFLIEIAS*ANS*NIVYKLSY@.D	3
*	PARC_p629-02.18357.18357.3	3.7566	0.0325	3061.16	3062.26	22	442.4	26.1%	1	R.VFHLLS*EWEKLASREY@SLANEMDL.I	3
*	PARC_p629-01.16530.16530.1	2.0886	0.0187	1171.81	1172.241	12	132.1	50.0%	1	L.FPSS*IPLYAS.A	1
*	PARC_p629-02.16386.16386.2	2.5661	0.0318	2484.83	2485.608	192	92.9	28.9%	1	I.ET#AT#KNMRTFKELAETSSFI.E	2
*	PARC_p629-05.11794.11794.3	3.6832	0.1315	4265.18	4266.019	15	281.5	16.4%	1	S.GELES*GTGLGS*GVGAEDITNT#LNEDDDLEELANEEDTAN.Q	3
*	PARC_p629-02.12080.12080.2	3.4648	0.066	2350.83	2351.451	51	241.1	38.9%	1	D.LVS*KEDDNKALEDKDRQEK.E	2
*	PARC_p629-03.16964.16964.3	3.5775	0.0908	3316.01	3315.345	7	535.3	23.2%	1	I.SIDDSKS*MSES*GS*TVLALETLALVT#KALS.L	3

U **SPBC32H8.13c** 6 7 5.5% 2352 266561 6.7 |mok12||alpha-1,3-glucan synthase|Schizosaccharomyces pombe|chr 2|||Manual

	Filename	XCorr	DeltCN	ObsM+H+	CalcM+H+	SpR	SpScore	Ion%	#	Sequence	
*	PARC_p629-01.15342.15342.2	2.5711	0.1013	1799.71	1798.905	41	304.7	39.3%	1	D.FTISTLSELSYFVNS*.S	2
*	PARC_p629-03.14137.14137.3	3.5845	0.0256	4506.86	4506.611	60	166.8	16.4%	1	Y.TDFQLSNGSSY@SCNAPTFWDVTGLPINNT#EDLNSISEVM.C	3
*	PARC_p629-02.11718.11718.2	2.644	0.04	1799.61	1800.965	1	484.6	53.8%	2	S.T#VKNLLYPYEEIDL.S	2
*	PARC_p629-02.16798.16798.3	3.6806	0.0746	3376.01	3375.309	29	363.8	21.3%	1	L.VY@PNNAT#FSPSLLYKASNGDLY@VNHT#GA.G	3
*	PARC_p629-02.12794.12794.2	3.0881	0.0486	2445.89	2445.668	2	455.2	42.1%	1	D.MHFKDGNSSKLEIVESKEIY@.A	2
*	PARC_p629-02.10678.10678.1	2.0068	0.0991	1523.63	1523.565	105	190.1	33.3%	1	C.FGLS*YVT#FAGLPA.F	1

U **SPCC63.08c** 3 3 5.4% 830 91585 7.7 |||serine/threonine protein kinase |Schizosaccharomyces pombe|chr 3|||Manual

	Filename	XCorr	DeltCN	ObsM+H+	CalcM+H+	SpR	SpScore	Ion%	#	Sequence	
*	PARC_p629-01.15956.15956.2	2.6129	0.0317	2119.69	2118.315	5	305.1	37.5%	1	E.ILRYEKYDAKADLWS*VG.A	2
*	PARC_p629-03.09895.09895.2	2.5148	0.0149	1443.51	1442.653	1	638.5	65.0%	1	H.T#SRMQIPYMKP.N	2
*	PARC_p629-02.13761.13761.2	2.8587	0.2609	1841.83	1842.758	1	334.0	46.9%	1	L.S*RNAATSELSGNDAQAC.L	2

U **SPAC3A11.09** 2 2 5.4% 759 85487 6.1 |||sodium/hydrogen exchanger family|Schizosaccharomyces pombe|chr 1|||Manual

	Filename	XCorr	DeltCN	ObsM+H+	CalcM+H+	SpR	SpScore	Ion%	#	Sequence	
*	PARC_p629-03.16039.16039.3	3.6626	0.1369	3480.05	3478.627	46	484.7	20.2%	1	G.SSIAFFMLGKRINT#LALS*FT#RT#RDSHF.A	3
*	PARC_p629-02.09385.09385.2	2.8792	0.1076	1544.21	1545.608	20	457.1	46.2%	1	N.GPS*IDEKLAQGDPK.A	2

U **SPBC36.07** 4 5 5.2% 1253 141607 5.5 |||RNA polymerase II |Schizosaccharomyces pombe|chr 2|||Manual

	Filename	XCorr	DeltCN	ObsM+H+	CalcM+H+	SpR	SpScore	Ion%	#	Sequence	
*	PARC_p629-01.13702.13702.2	2.5093	0.1002	1781.95	1780.942	2	281.1	50.0%	1	S.LLS*WKPS*GSVLATIK.S	2
*	PARC_p629-02.15509.15509.2	2.6807	0.0168	2214.79	2214.111	39	265.0	37.5%	1	Q.TSS*GKLIS*Y@NLNVKS*IE.S	2
*	PARC_p629-03.16534.16534.3	4.0839	0.0328	4078.34	4079.842	85	338.2	19.5%	2	T.MEDQTPDDISLAST#S*LST#NRSLY@T#QYTKSSNSS.K	3
*	PARC_p629-02.18429.18429.3	4.3474	0.0323	4080.74	4079.842	30	166.9	19.5%	1	T.MEDQTPDDISLAST#S*LST#NRS*LY@TQYTKSSNSS.K	3

U **SPAC4A8.16c** 4 4 5.2% 918 104367 4.9 |tif33|SPAC823.01c|eukaryotic translation initiation factor eIF3c|Schizosaccharomyces pombe|chr 1|||Manual

	Filename	XCorr	DeltCN	ObsM+H+	CalcM+H+	SpR	SpScore	Ion%	#	Sequence	
*	PARC_p629-02.12955.12955.2	2.6378	0.0353	1766.29	1766.471	1	475.3	50.0%	1	E.S*SEEES*ESEESEVE.V	2
*	PARC_p629-02.12003.12003.2	2.5122	0.046	1738.37	1738.859	364	160.2	30.0%	1	S.ADKAFIKS*LDAANGRA.F	2
*	PARC_p629-03.11491.11491.2	2.8851	0.0824	2229.07	2228.31	1	643.0	47.1%	1	L.VRSQGY@LERIGVVENT#AR.L	2
*	PARC_p629-02.12046.12046.2	3.1029	0.0225	2229.61	2228.31	1	592.6	44.1%	1	L.VRS*QGYLERIGVVENT#AR.L	2

U **SPAC1006.06** 9 10 4.7% 1158 130879 9.3 |rgf2||GDP-GTP exchange protein for Rho1p |Schizosaccharomyces pombe|chr 1|||Manual

	Filename	XCorr	DeltCN	ObsM+H+	CalcM+H+	SpR	SpScore	Ion%	#	Sequence	
*	PARC_p629-02.17292.17292.3	3.5429	0.1264	4191.32	4192.252	120	385.6	16.7%	1	Y.TKRRANTSDSFDSVLSDSST#TPTISSSVQVNS*LAFIT#.S	3
*	PARC_p629-04.15494.15494.3	3.623	0.0559	3347.87	3348.848	34	389.7	22.0%	1	K.RRANT#SDSFDSVLS*DSST#TPT#IS*S*S*V.Q	3
*	PARC_p629-03.16361.16361.3	4.6222	0.0178	4092.47	4091.88	199	447.8	19.1%	1	D.SFDS*VLSDSSTTPTISSSVQVNS*LAFIT#SS*LS*AIT#.K	3
*	PARC_p629-03.16952.16952.3	3.8179	0.0189	4093.31	4091.88	38	423.5	18.4%	1	D.S*FDSVLSDSSTTPTISSSVQVNS*LAFIT#SS*LS*AIT#.K	3
*	PARC_p629-01.17853.17853.2	3.212	0.0476	1830.41	1829.543	13	393.6	53.6%	2	D.S*VLSDS*S*T#TPTISSS.V	2
*	PARC_p629-03.13760.13760.3	3.6669	0.0112	4099.67	4099.865	213	162.6	16.7%	1	S.VLS*DSS*T#T#PT#ISSS*VQVNSLAFITSSLSAIT#KEP.E	3
*	PARC_p629-02.17933.17933.3	3.6651	0.0248	4073.09	4074.072	118	312.1	19.1%	1	T.TPTISSSVQVNS*LAFITSS*LSAIT#KEPEAPETEY@N.P	3
*	PARC_p629-03.17290.17290.3	4.026	0.0362	4073.84	4074.072	43	335.8	19.9%	1	T.TPTIS*S*S*VQVNSLAFITSSLSAITKEPEAPETEY@N.P	3
*	PARC_p629-03.17381.17381.3	3.8091	0.0136	4075.07	4074.072	4	385.9	20.6%	1	T.T#PTISS*S*VQVNSLAFITSSLSAITKEPEAPETEY@N.P	3

U **SPBC1289.10c** 2 4 4.6% 743 81159 7.2 |||transcriptional regulator |Schizosaccharomyces pombe|chr 2|||Manual

	Filename	XCorr	DeltCN	ObsM+H+	CalcM+H+	SpR	SpScore	Ion%	#	Sequence	
*	PARC_p629-02.11878.11878.2	2.9023	0.1079	1854.45	1854.811	4	284.9	43.3%	3	S.S*PKTEGAPSNAQFRPS*.L	2
*	PARC_p629-03.15945.15945.2	2.6194	0.1341	2326.05	2327.158	2	205.2	32.4%	1	G.QFSGNQMNQY@GFS*NS*PYS.Q	2

U **SPAC1805.17** 2 2 4.4% 1078 123567 5.2 |crm1|caf2, SPAC1B2.01|chromosome region maintenance protein 1|Schizosaccharomyces pombe|chr 1|||Manual

	Filename	XCorr	DeltCN	ObsM+H+	CalcM+H+	SpR	SpScore	Ion%	#	Sequence	
*	PARC_p629-02.11269.11269.1	2.3672	0.1157	1547.69	1548.657	94	137.7	37.5%	1	S.IGS*GFYPQIAKNY.V	1
*	PARC_p629-03.16544.16544.3	4.0755	0.0365	4097.54	4097.254	36	473.4	17.4%	1	A.SMGPDVS*NAFFQTYYISLLQDILYVLT#DS*DHKSG.F	3

U **SPBP35G2.06c** 2 2 4.3% 1142 131439 5.6 |nup131|Nup133a|nucleoporin |Schizosaccharomyces pombe|chr 2|||Manual

	Filename	XCorr	DeltCN	ObsM+H+	CalcM+H+	SpR	SpScore	Ion%	#	Sequence	
*	PARC_p629-05.09283.09283.3	3.6128	0.0135	3333.8	3335.161	21	532.6	26.1%	1	I.ILDS*IQES*FSY@VNRFLILDFS*VS*T#.S	3
*	PARC_p629-02.12651.12651.3	3.7296	0.024	3137.69	3138.252	7	478.9	25.0%	1	F.LLNFDFT#YNLSESEVYNTVFNLCDE.L	3

U **SPBC17D11.05** 3 3 4.3% 932 107071 8.9 |tif32||eukaryotic translation initiation factor eIF3a|Schizosaccharomyces pombe|chr 2|||Manual

	Filename	XCorr	DeltCN	ObsM+H+	CalcM+H+	SpR	SpScore	Ion%	#	Sequence	
*	PARC_p629-02.08539.08539.1	2.1771	0.0484	1064.68	1063.207	8	75.1	57.1%	1	E.T#IVLKRSR.N	1
*	PARC_p629-03.09331.09331.2	2.9936	0.107	1651.43	1651.732	3	347.0	50.0%	1	L.QSTPSELITS*QLTR.I	2
*	PARC_p629-05.12794.12794.2	2.6575	0.0779	2213.93	2215.062	5	260.9	32.4%	1	G.GVS*RDS*PRYSRGGY@SRGS.V	2

U **SPBC14C8.14c** 3 5 4.2% 959 109719 5.7 |pol5||DNA polymerase V|Schizosaccharomyces pombe|chr 2|||Manual

	Filename	XCorr	DeltCN	ObsM+H+	CalcM+H+	SpR	SpScore	Ion%	#	Sequence	
*	PARC_p629-02.08210.08210.1	1.8186	0.0939	1262.55	1262.324	2	450.1	66.7%	1	L.LELEKS*PKNN.L	1
*	PARC_p629-02.12120.12120.2	2.8203	0.0209	2212.05	2213.345	111	323.1	32.4%	2	K.NNLLIS*MDESVIEIVQKS*.L	2
*	PARC_p629-02.13771.13771.2	2.5538	0.0262	1772.75	1771.47	37	285.3	34.6%	2	K.AS*LGS*T#GS*SIS*QLL.L	2

U **SPAC644.12** 2 2 4.1% 757 86844 7.9 |cdc5||transcriptional regulator|Schizosaccharomyces pombe|chr 1|||Manual

	Filename	XCorr	DeltCN	ObsM+H+	CalcM+H+	SpR	SpScore	Ion%	#	Sequence	
*	PARC_p629-02.16171.16171.1	1.9639	0.1365	1376.8	1375.31	3	170.2	42.3%	1	I.S*GEGAEAAAPVNDP.N	1
*	PARC_p629-02.19344.19344.2	2.5778	0.0258	2215.53	2214.113	52	155.8	34.4%	1	A.LIS*S*DAINY@PFGNS*KVK.G	2

U **SPBC1105.10** 3 3 4.0% 1297 148739 6.8 |||WD repeat protein |Schizosaccharomyces pombe|chr 2|||Manual

	Filename	XCorr	DeltCN	ObsM+H+	CalcM+H+	SpR	SpScore	Ion%	#	Sequence	
*	PARC_p629-02.11777.11777.1	1.8663	0.0546	1334.66	1333.361	2	154.4	55.6%	1	T.QLS*WGYNFDL.L	1
*	PARC_p629-02.15721.15721.2	2.5236	0.1187	2215.05	2214.281	11	315.6	31.6%	1	F.ILPVQNNDNSALCALT#EGGH.A	2
*	PARC_p629-01.16741.16741.2	3.2436	0.0423	2509.51	2508.538	1	893.2	45.2%	1	E.Y@T#NSLISDSSWLDNGTLVTAIG.N	2

U **SPAC13G7.01c** 2 3 4.0% 721 82819 6.1 |erg7|SPAC4G9.21c|lanosterol synthase |Schizosaccharomyces pombe|chr 1|||Manual

	Filename	XCorr	DeltCN	ObsM+H+	CalcM+H+	SpR	SpScore	Ion%	#	Sequence	
*	PARC_p629-04.09246.09246.2	2.9074	0.1441	1413.61	1414.535	7	454.0	65.0%	2	I.ENALEY@VVKMQ.R	2
*	p629-05ms3.08518.08518.2	2.8834	0.1388	2067.69	2068.357	1	161.4	35.3%	1	R.KPIEKGIKFLMASQKS*DG.S	2

U **SPAC17A5.14** 2 2 3.7% 1328 152589 7.3 |exo2||exonuclease II |Schizosaccharomyces pombe|chr 1|||Manual

	Filename	XCorr	DeltCN	ObsM+H+	CalcM+H+	SpR	SpScore	Ion%	#	Sequence	
*	PARC_p629-05.10814.10814.3	3.5864	0.0503	4083.95	4083.359	25	232.7	16.7%	1	P.PCFRDLMVNS*ESPIIDFYPENFALDQNGKTASWE.A	3
*	PARC_p629-03.13354.13354.2	2.7027	0.0652	1862.55	1864.032	5	259.9	39.3%	1	L.LNIHPLLLSKIT#SS*F.L	2

U **SPAC17A5.16** 3 8 3.2% 925 104836 6.6 |||conserved eukaryotic protein|Schizosaccharomyces pombe|chr 1|||Manual

	Filename	XCorr	DeltCN	ObsM+H+	CalcM+H+	SpR	SpScore	Ion%	#	Sequence	
*	PARC_p629-03.09951.09951.2	2.8594	0.0306	1757.41	1757.905	6	423.9	57.7%	5	N.KFSQNPNLSY@SIIR.L	2
*	PARC_p629-03.10354.10354.2	2.8732	0.031	1757.59	1757.905	31	292.8	46.2%	2	N.KFSQNPNLS*YSIIR.L	2
*	PARC_p629-01.12297.12297.2	3.5157	0.0443	1987.29	1985.944	1	774.9	56.7%	1	H.IS*SRNVS*ISVPT#VLQD.V	2

U **SPAC15A10.11** 4 4 2.3% 2052 234041 5.3 |ubr11||ubiquitin-protein ligase |Schizosaccharomyces pombe|chr 1|||Manual

	Filename	XCorr	DeltCN	ObsM+H+	CalcM+H+	SpR	SpScore	Ion%	#	Sequence	
*	PARC_p629-03.16331.16331.3	3.6795	0.0354	3824.39	3824.648	1	515.7	20.7%	1	L.AEAVVKDY@DFLT#NLNAT#TLSLLT#QSNRPS*T#.L	3
*	PARC_p629-04.17736.17736.3	3.6285	0.0173	3824.54	3824.648	5	591.8	21.6%	1	L.AEAVVKDY@DFLT#NLNATTLS*LLT#QSNRPS*T#.L	3
*	PARC_p629-01.15690.15690.2	2.8785	0.0961	2117.79	2117.065	1	423.6	53.1%	1	F.NEFSNNEDDSELERLS*K.L	2
*	PARC_p629-01.15919.15919.2	2.8402	0.1837	2115.23	2116.121	2	200.3	46.9%	1	N.EFSNNEDDSELERLS*KL.I	2

U **SPAC9.03c** 3 7 1.7% 2176 248807 5.2 |brr2||U4/U6 x U5 tri-snRNP complex |Schizosaccharomyces pombe|chr 1|||Manual

	Filename	XCorr	DeltCN	ObsM+H+	CalcM+H+	SpR	SpScore	Ion%	#	Sequence	
*	PARC_p629-02.08604.08604.1	2.0524	0.0842	1064.69	1064.012	12	67.8	50.0%	2	I.T#IPDDGLNE.L	1
*	PARC_p629-03.11116.11116.2	2.6562	0.0212	1523.67	1524.765	13	425.7	54.5%	4	L.QNFKIVY@IAPLK.A	2
*	PARC_p629-03.10212.10212.2	2.5042	0.0655	1756.33	1755.762	2	438.5	50.0%	1	Y.NRQSGT#LT#ATELGKV.A	2

U **SPCC895.09c** 1 25 1.4% 1327 149880 8.6 |||RNA helicase|Schizosaccharomyces pombe|chr 3|||Manual

	Filename	XCorr	DeltCN	ObsM+H+	CalcM+H+	SpR	SpScore	Ion%	#	Sequence	
*	PARC_p629-02.12187.12187.2	3.81	0.1462	2213.75	2214.23	28	533.8	38.2%	25	E.WDDDVS*ALQSILDNKVS*K.I	2

	Proteins	Peptide IDs	Copies
Unfiltered	314	76440	91444
Redundant	136	696	1152
Nonredundant	132	689	1145

mts3-1 dma1 deletion dnt1 deletion
+ pREP41-NTAP-dma1(RF deletion)

DTASelect v1.9
SEQUEST 2.7 in SQT format.

Jump to the summary table.

sequest.params modifications:

*	ST	0.0
#	M	0.0
@	C	0.0
Static	C	57.0

true	Use criteria
1.6	Minimum +1 XCorr
2.3	Minimum +2 XCorr
3.5	Minimum +3 XCorr
4.5	Minimum +4 XCorr
0.08	Minimum DeltCN
1	Minimum charge state
9	Maximum charge state
0.0	Minimum ion proportion
1000	Maximum Sp rank
-1.0	Minimum Sp score
Include	Modified peptide inclusion
Any	Tryptic status requirement
true	Multiple, ambiguous IDs allowed
Ignore	Peptide validation handling
XCorr	Purge duplicate peptides by protein
false	Include only loci with unique peptide
false	Remove subset proteins
Ignore	Locus validation handling
0	Minimum modified peptides per locus
10	Minimum redundancy for low coverage loci
4	Minimum peptides per locus

Locus Key:

Validation Status	Locus	Sequence Count	Spectrum Count	Sequence Coverage	Length	MolWt	pI	Descriptive Name

Similarity Key:

Locus	# of identical peptides	# of differing peptides

U	**SPCC24B10.09**	6	27	37.9%	132	15534	10.1	|rps1702|rps17-2, rps17|40S ribosomal protein S17|Schizosaccharomyces pombe|chr 3|||Manual

	Filename	XCorr	DeltCN	ObsM+H+	CalcM+H+	SpR	SpScore	Ion%	#	Sequence	
	3264plusp804-02.10524.10524.2	3.1203	0.2509	1080.3322	1080.2261	1	854.7	87.5%	6	R.LTLDFQTNK.R	22
	3264plusp804-02.11033.11033.1	2.5101	0.1482	1157.66	1158.3806	116	430.9	50.0%	3	R.IVDEVAIIASK.R	11
	3264plusp804-02.11208.11208.2	4.5979	0.3882	1158.4521	1158.3806	1	1246.8	90.0%	13	R.IVDEVAIIASK.R	22
*	3264plusp804-02.11661.11661.2	3.6129	0.304	1566.4122	1564.7325	1	703.0	66.7%	3	R.KDQYVPEVSELEK.D	2
*	3264plusp804-02.14264.14264.2	4.3118	0.4917	1619.9521	1619.8583	1	832.4	70.0%	1	K.ALGYDSIPTVVVAASR.P	2
*	3264plusp804-02.14924.14924.2	4.5615	0.4365	1718.1921	1716.975	1	738.3	59.4%	1	K.ALGYDSIPTVVVAASRP.E	2

Similarities: SPBC839.05c(3:3)

U	**SPAC17G8.10c**	37	264	37.5%	267	30597	6.9	|dma1||zinc finger protein|Schizosaccharomyces pombe|chr 1|||Manual

	Filename	XCorr	DeltCN	ObsM+H+	CalcM+H+	SpR	SpScore	Ion%	#	Sequence	
*	3264plusp804-02.08209.08209.1	1.632	0.0907	795.61	795.9109	116	279.5	58.3%	1	K.SVEGYLK.E	1
*	3264plusp804-02.08894.08894.2	2.4905	0.1309	1181.1721	1182.2726	7	624.2	66.7%	2	K.SVEGYLKEQE.L	2
*	3264plusp804-02.10542.10542.2	2.9541	0.2231	1367.0721	1366.5107	2	528.1	63.6%	2	K.SVEGYLKEQELA.A	2
*	3264plusp804-02.10757.10757.2	3.251	0.2051	1439.4122	1437.5896	121	191.4	45.8%	1	K.SVEGYLKEQELAA.E	2
*	3264plusp804-02.10808.10808.2	3.6057	0.3035	1565.9321	1566.7051	2	661.5	57.7%	15	K.SVEGYLKEQELAAE.T	2
*	3264plusp804-02.11481.11481.2	2.7612	0.2183	1666.5521	1667.8102	5	265.8	46.4%	4	K.SVEGYLKEQELAAET.D	2
*	3264plusp804-02.11012.11012.2	4.2605	0.4392	1783.3922	1782.8987	1	1565.1	73.3%	12	K.SVEGYLKEQELAAETD.S	2
*	3264plusp804-02.11302.11302.2	3.826	0.3525	1869.1921	1869.9769	1	1276.2	59.4%	4	K.SVEGYLKEQELAAETDS.E	2
*	3264plusp804-02.11645.11645.2	4.2081	0.3937	1998.8121	1999.0924	1	1329.5	61.8%	4	K.SVEGYLKEQELAAETDSE.K	2
*	3264plusp804-02.11465.11465.2	6.4365	0.5226	2127.6921	2127.2666	1	2221.9	66.7%	41	K.SVEGYLKEQELAAETDSEK.D	2
*	3264plusp804-02.11993.11993.3	5.4439	0.3104	2128.3743	2127.2666	1	1315.2	44.4%	25	K.SVEGYLKEQELAAETDSEK.D	3
*	3264plusp804-	5.9175	0.4802	2241.672	2242.355	1	1872.6	60.5%	4	K.SVEGYLKEQELAAETDSEKD.D	2

	02.11698.11698.2										
*	3264plusp804-02.11759.11759.2	5.2986	0.5164	2356.912	2357.4436	1	1238.4	55.0%	1	K.SVEGYLKEQELAAETDSEKDD.D	2
*	3264plusp804-02.12471.12471.3	5.1574	0.3779	2600.5444	2600.7063	1	1349.4	38.6%	9	K.SVEGYLKEQELAAETDSEKDDDK.I	3
*	3264plusp804-02.11446.11446.3	3.7778	0.2757	2801.8743	2800.944	1	888.9	28.1%	1	K.SVEGYLKEQELAAETDSEKDDDKIS.I	3
*	3264plusp804-02.12001.12001.3	7.4073	0.4921	3070.9744	3070.291	1	1992.6	36.5%	85	K.SVEGYLKEQELAAETDSEKDDDKISIR.L	3
*	3264plusp804-02.04893.04893.2	4.7544	0.4397	2293.652	2293.4033	1	530.9	44.7%	1	K.EQELAAETDSEKDDDKISIR.L	2
*	3264plusp804-02.14740.14740.2	3.3194	0.268	1653.7522	1653.7911	1	649.6	60.7%	2	R.LTNFVGPNAHSFSFD.P	2
*	3264plusp804-05.00442.00442.2	4.7713	0.4234	2117.632	2119.3872	1	705.3	55.6%	1	R.LTNFVGPNAHSFSFDPLVR.Y	2
*	3264plusp804-02.14668.14668.2	2.6924	0.2606	1068.2722	1068.2175	1	673.7	87.5%	4	H.SFSFDPLVR.Y	2
*	3264plusp804-05.00629.00629.2	2.7542	0.3195	2265.5322	2266.5156	1	459.8	36.8%	1	K.TSKPYPISNNDILQLGADYR.G	2
*	3264plusp804-05.00237.00237.3	4.3405	0.3868	2265.6243	2266.5156	1	1470.5	39.5%	1	K.TSKPYPISNNDILQLGADYR.G	3
*	3264plusp804-02.15458.15458.3	3.608	0.2929	1949.3644	1950.1582	1	1581.6	46.9%	1	K.PYPISNNDILQLGADYR.G	3
*	3264plusp804-02.15476.15476.2	5.8888	0.5551	1949.6721	1950.1582	1	1254.5	71.9%	6	K.PYPISNNDILQLGADYR.G	2
*	3264plusp804-02.14853.14853.2	4.0659	0.3764	1851.6921	1853.0416	1	1078.6	63.3%	3	P.YPISNNDILQLGADYR.G	2
*	3264plusp804-02.13590.13590.2	3.7232	0.3935	1689.5122	1689.8656	1	1135.2	64.3%	1	Y.PISNNDILQLGADYR.G	2
*	3264plusp804-02.12935.12935.2	4.1585	0.4428	1592.5322	1592.7489	1	1624.2	73.1%	1	P.ISNNDILQLGADYR.G	2
*	3264plusp804-02.12073.12073.2	3.5048	0.3836	1481.4122	1479.5895	1	1280.1	79.2%	2	I.SNNDILQLGADYR.G	2
*	3264plusp804-02.12151.12151.2	3.201	0.3675	1392.3922	1392.5114	1	1163.4	72.7%	1	S.NNDILQLGADYR.G	2
*	3264plusp804-02.12125.12125.2	2.9483	0.4482	1277.8922	1278.4075	1	1192.5	85.0%	1	N.NDILQLGADYR.G	2
*	3264plusp804-02.12406.12406.2	3.1854	0.2385	1164.1322	1164.3036	1	1171.2	83.3%	2	N.DILQLGADYR.G	2
*	3264plusp804-02.10186.10186.2	2.805	0.1461	1050.6322	1049.215	49	783.8	81.2%	1	D.ILQLGADYR.G	2
*	3264plusp804-02.09102.09102.2	3.0455	0.1512	990.2922	990.1039	3	439.0	85.7%	7	R.VELNNSWK.I	2
*	3264plusp804-02.11678.11678.2	2.4426	0.4132	1224.8522	1225.3867	7	200.5	77.8%	1	K.LSPYNLNEFK.R	2
*	3264plusp804-02.10128.10128.2	2.9881	0.2659	1111.6322	1112.2273	1	1214.0	87.5%	4	L.SPYNLNEFK.R	2
*	3264plusp804-01.17314.17314.2	3.5543	0.2818	1723.0721	1720.855	1	532.5	56.7%	3	R.MQELVLCGSSESGPPE.C	2
*	3264plusp804-01.17760.17760.1	2.277	0.2901	848.21	846.826	14	235.8	50.0%	9	C.GSSESGPPE.C	1

U **SPCC1739.13** 20 53 36.9% 647 70233 5.2 |ssa2||heat shock protein 70 family|Schizosaccharomyces pombe|chr 3|||Manual

	Filename	XCorr	DeltCN	ObsM+H+	CalcM+H+	SpR	SpScore	Ion%	#	Sequence	
	3264plusp804-02.10261.10261.2	3.6938	0.4735	1488.2922	1488.5939	1	717.3	75.0%	8	R.TTPSYVAFTDTER.L	22
*	3264plusp804-02.18167.18167.2	3.9949	0.3847	1613.4922	1613.9246	1	821.3	73.1%	3	K.TFTPEEISSMVLMK.M	2
	3264plusp804-02.12585.12585.2	3.5939	0.4787	1883.4321	1883.0679	1	916.0	75.0%	2	K.VTDAVVTVPAYFNDSQR.Q	22
	3264plusp804-02.16121.16121.2	4.1844	0.3788	1212.0322	1212.4349	1	1763.1	90.9%	4	K.DAGLIAGLNVLR.I	22
	3264plusp804-02.13936.13936.2	5.5192	0.5595	1689.4122	1688.9213	1	2002.0	83.3%	3	R.IINEPTAAAIAYGLDR.S	22
	3264plusp804-02.13951.13951.3	4.6666	0.3424	1689.8043	1688.9213	1	1388.7	48.3%	2	R.IINEPTAAAIAYGLDR.S	33
*	3264plusp804-02.10812.10812.2	3.805	0.4607	1402.3722	1402.5907	1	927.7	65.4%	4	K.ASVNEIVLVGGSTR.I	2
*	3264plusp804-02.18244.18244.2	6.7056	0.5084	2578.172	2578.7925	1	2153.3	54.0%	2	K.SINPDEAVAYGAAVQAAVLTGDTSEK.T	2
*	3264plusp804-02.18277.18277.3	4.3073	0.4213	2578.6143	2578.7925	1	902.3	31.0%	1	K.SINPDEAVAYGAAVQAAVLTGDTSEK.T	3
*	3264plusp804-02.20066.20066.3	5.417	0.4838	2615.3044	2616.8438	1	3061.4	43.2%	4	K.SEIFSTYSDNQPGVLIQVFEGER.A	3
*	3264plusp804-02.20037.20037.2	5.4092	0.4725	2616.5522	2616.8438	1	1091.3	52.3%	4	K.SEIFSTYSDNQPGVLIQVFEGER.A	2
*	3264plusp804-02.17452.17452.2	4.8684	0.4889	2139.9321	2140.314	1	1081.3	61.1%	1	F.STYSDNQPGVLIQVFEGER.A	2
*	3264plusp804-02.16739.16739.2	4.7616	0.5657	1788.2322	1788.9548	1	1088.3	66.7%	2	Y.SDNQPGVLIQVFEGER.A	2
	3264plusp804-02.11471.11471.2	2.3053	0.2725	1184.4521	1184.3806	1	568.1	65.0%	1	K.FELSGIPPAPR.G	22
	3264plusp804-02.17700.17700.2	2.6038	0.4048	2034.4122	2035.2585	1	434.3	38.9%	1	I.EVTFDVDANGILNVSALEK.G	22
*	3264plusp804-04.00296.00296.2	2.3159	0.108	1535.7922	1535.6482	8	278.0	50.0%	1	V.DASDKETVDKAVKE.T	2
*	3264plusp804-02.11362.11362.2	4.0857	0.4102	1579.5122	1579.7039	1	1565.4	76.9%	2	K.ETIEWLDSNTTAAK.D	2
*	3264plusp804-02.13094.13094.2	4.8379	0.4701	2298.632	2299.453	1	1293.2	57.9%	2	K.ETIEWLDSNTTAAKDEFEAK.Q	2
*	3264plusp804-02.10016.10016.2	3.9181	0.4023	1301.9722	1302.5286	1	1061.9	81.8%	5	K.ELESVANPIMAK.I	2
*	3264plusp804-01.17602.17602.2	2.3157	0.2232	2361.372	2361.6372	49	202.3	19.6%	1	K.IYQAGGAPGGMPGAAPGAAPGAAPGAAPG.G	2

Similarities: SPAC13G7.02c(7:13)

U **SPCC74.05** 5 11 36.8% 136 15305 10.7 |rpl2702|rpl27-2|60S ribosomal protein L27|Schizosaccharomyces pombe|chr 3|||Manual

	Filename	XCorr	DeltCN	ObsM+H+	CalcM+H+	SpR	SpScore	Ion%	#	Sequence	
*	3264plusp804-02.09229.09229.2	2.6713	0.2027	1301.1122	1300.4978	1	714.4	63.6%	1	K.VVILQNVDQGSK.S	2
	3264plusp804-01.17924.17924.1	2.2794	0.1599	1664.44	1664.0624	20	342.8	46.4%	1	R.YPLKVTKSMGAKRIA.K	11
	3264plusp804-02.11738.11738.1	2.3338	0.2646	1078.52	1079.2383	1	605.8	75.0%	1	R.YALELDNLK.G	11
	3264plusp804-02.11773.11773.2	3.1586	0.1708	1078.9321	1079.2383	1	972.4	93.8%	2	R.YALELDNLK.G	22
*	3264plusp804-02.10216.10216.2	3.5626	0.4288	1519.9521	1520.6824	1	980.5	69.2%	6	K.GLVTPTTFSEPSQR.S	2

Similarities: SPBC685.07c(3:2)

U **SPAC890.08** 10 24 32.7% 113 13261 10.2 |rpl31||60S ribosomal protein L31|Schizosaccharomyces pombe|chr 1|||Manual

	Filename	XCorr	DeltCN	ObsM+H+	CalcM+H+	SpR	SpScore	Ion%	#	Sequence	
*	3264plusp804-02.09103.09103.1	1.9654	0.1432	905.59	906.06964	2	382.0	78.6%	2	K.EIVAFAQK.H	1
*	3264plusp804-02.13891.13891.2	6.3835	0.6304	2341.632	2342.5212	1	2735.4	67.5%	1	R.SDEDDKALYTYVQAVDVANPK.M	2
*	3264plusp804-02.15995.15995.3	4.222	0.482	3261.0244	3261.5354	1	504.2	25.9%	1	R.SDEDDKALYTYVQAVDVANPKMETTVVEE.-	3
*	3264plusp804-02.12223.12223.1	3.7681	0.4182	1652.64	1652.8877	1	795.1	57.1%	2	K.ALYTYVQAVDVANPK.M	1
*	3264plusp804-02.12305.12305.2	4.7426	0.5026	1652.8121	1652.8877	1	1071.0	71.4%	5	K.ALYTYVQAVDVANPK.M	2
*	3264plusp804-02.12214.12214.3	4.5961	0.4317	1652.8744	1652.8877	1	2644.9	58.9%	3	K.ALYTYVQAVDVANPK.M	3
*	3264plusp804-02.15040.15040.2	5.0015	0.4816	2572.5923	2571.9019	1	925.9	47.7%	6	K.ALYTYVQAVDVANPKMETTVVEE.-	2
*	3264plusp804-02.14993.14993.3	4.3192	0.4012	2572.8542	2571.9019	1	832.2	37.5%	2	K.ALYTYVQAVDVANPKMETTVVEE.-	3
*	3264plusp804-02.11158.11158.2	4.1857	0.4458	1583.1322	1581.8088	1	1693.7	73.1%	1	A.LYTYVQAVDVANPK.M	2
*	3264plusp804-02.09577.09577.2	2.4434	0.1462	1470.1522	1468.6494	1	1007.4	75.0%	1	L.YTYVQAVDVANPK.M	2

U **SPBC1711.06** 7 25 28.1% 363 39910 10.7 |rpl401|rpl4-1, rpl4|60S ribosomal protein L2|Schizosaccharomyces pombe|chr 2|||Manual

	Filename	XCorr	DeltCN	ObsM+H+	CalcM+H+	SpR	SpScore	Ion%	#	Sequence	
*	3264plusp804-02.01011.01011.2	2.7488	0.1553	1842.4722	1842.0557	3	439.4	43.8%	5	S.IYNKDGSVSSETLALPF.V	2
*2	3264plusp804-02.19135.19135.2	4.9113	0.4906	1697.4521	1697.9255	1	1677.5	80.0%	6	K.DGSVSSETLALPFVFK.A	22
	3264plusp804-02.20892.20892.2	4.4255	0.4162	2255.892	2256.5579	1	1139.0	65.8%	3	R.IEEIPEVPLVVDDAVQSFQK.T	22
*	3264plusp804-02.13884.13884.2	2.3909	0.1667	1488.4722	1488.724	2	378.8	46.2%	1	R.GPLVVFNEDTGIVK.A	2
*	3264plusp804-05.00190.00190.2	5.1322	0.3913	2070.872	2071.335	1	1695.2	60.5%	2	K.SAFGLLDSVFGSTTEVAQLK.K	2
	3264plusp804-02.16505.16505.2	5.0062	0.484	1866.6522	1867.0685	1	1935.6	86.7%	6	K.NYFLPENIISNADVTR.L	22
	3264plusp804-02.11897.11897.2	4.5265	0.2166	1361.4321	1359.5626	1	1573.4	90.9%	2	R.LINSDEIQSIVK.A	22

Similarities: SPBP8B7.03c(4:3)

U **SPBC685.07c** 4 5 26.5% 136 15389 10.9 |rpl2701|rpl27-1|60S ribosomal protein L27|Schizosaccharomyces pombe|chr 2|||Manual

	Filename	XCorr	DeltCN	ObsM+H+	CalcM+H+	SpR	SpScore	Ion%	#	Sequence	
*	3264plusp804-02.11341.11341.2	3.3785	0.4846	1270.6522	1271.4998	1	1717.7	86.4%	1	K.VVILQAIDQGSK.S	2
	3264plusp804-01.17924.17924.1	2.2794	0.1599	1664.44	1664.0624	20	342.8	46.4%	1	R.YPLKVTKSMGAKRIA.R	11
	3264plusp804-02.11738.11738.1	2.3338	0.2646	1078.52	1079.2383	1	605.8	75.0%	1	R.YALELDNLK.G	11
	3264plusp804-02.11773.11773.2	3.1586	0.1708	1078.9321	1079.2383	1	972.4	93.8%	2	R.YALELDNLK.G	22

Similarities: SPCC74.05(3:1)

U **SPBC106.18** 4 16 24.8% 141 15836 10.5 |rpl25a||60S ribosomal protein L25|Schizosaccharomyces pombe|chr 2|||Manual

U **SPBC4F6.04** 4 16 24.8% 141 15768 10.5 |rpl2502|rpl25b, rpl23a-2|60S ribosomal protein L25|Schizosaccharomyces pombe|chr 2|||Manual

Filename	XCorr	DeltCN	ObsM+H+	CalcM+H+	SpR	SpScore	Ion%	#	Sequence	
3264plusp804-02.12559.12559.2	5.4916	0.355	2064.872	2065.3894	1	1929.5	67.6%	3	R.LDEYKIIVNPINSESAMK.K	2
3264plusp804-02.10322.10322.2	3.5623	0.2924	1417.2122	1416.6758	1	1459.0	83.3%	6	K.IIVNPINSESAMK.K	2
3264plusp804-02.04954.04954.2	4.6189	0.4384	1331.2122	1331.4252	1	2809.0	91.7%	1	K.LSADADALDVANR.I	2
3264plusp804-02.18979.18979.2	5.1765	0.4871	1762.7922	1761.9725	1	2024.9	75.0%	6	K.LSADADALDVANRIGFL.-	2

U **SPAC13G7.02c** 11 28 23.3% 644 70143 5.2 |ssa1||heat shock protein 70 family|Schizosaccharomyces pombe|chr 1|||Manual

	Filename	XCorr	DeltCN	ObsM+H+	CalcM+H+	SpR	SpScore	Ion%	#	Sequence	
	3264plusp804-02.10261.10261.2	3.6938	0.4735	1488.2922	1488.5939	1	717.3	75.0%	8	R.TTPSYVAFTDTER.L	22
	3264plusp804-02.12585.12585.2	3.5939	0.4787	1883.4321	1883.0679	1	916.0	75.0%	2	K.VTDAVVTVPAYFNDSQR.Q	22
	3264plusp804-02.16121.16121.2	4.1844	0.3788	1212.0322	1212.4349	1	1763.1	90.9%	4	K.DAGLIAGLNVLR.I	22
	3264plusp804-02.13936.13936.2	5.5192	0.5595	1689.4122	1688.9213	1	2002.0	83.3%	3	R.IINEPTAAAIAYGLDR.S	22
	3264plusp804-02.13951.13951.3	4.6666	0.3424	1689.8043	1688.9213	1	1388.7	48.3%	2	R.IINEPTAAAIAYGLDR.S	33
*	3264plusp804-02.10556.10556.2	3.2909	0.2813	1419.3322	1418.5901	1	1182.4	69.2%	3	K.SSVNEIVLVGGSTR.I	2
*	3264plusp804-02.19869.19869.3	4.991	0.3731	2586.2344	2586.8174	1	1464.7	37.5%	1	K.SEVFSTYADNQPGVLIQVFEGER.A	3
*	3264plusp804-02.19800.19800.2	5.4711	0.3961	2586.4922	2586.8174	1	820.6	47.7%	2	K.SEVFSTYADNQPGVLIQVFEGER.A	2
	3264plusp804-02.11471.11471.2	2.3053	0.2725	1184.4521	1184.3806	1	568.1	65.0%	1	K.FELSGIPPAPR.G	22
	3264plusp804-	2.6038	0.4048	2034.4122	2035.2585	1	434.3	38.9%	1	I.EVTFDVDANGILNVSALEK.G	22

	02.17700.17700.2										
*	3264plusp804-02.05198.05198.3	4.0997	0.3384	2729.4243	2730.0012	1	972.1	31.2%	1	R.NSLDDPNLKDKVDASDKEAIDKAVK.E	3

Similarities: SPCC1739.13(7:4)

U **SPBC839.05c** 5 42 22.9% 131 15514 10.3 |rps1701|rps17-1|40S ribosomal protein S17|Schizosaccharomyces pombe|chr 2|||Manual

	Filename	XCorr	DeltCN	ObsM+H+	CalcM+H+	SpR	SpScore	Ion%	#	Sequence	
	3264plusp804-02.10524.10524.2	3.1203	0.2509	1080.3322	1080.2261	1	854.7	87.5%	6	R.LTLDFQTNK.R	22
	3264plusp804-02.11033.11033.1	2.5101	0.1482	1157.66	1158.3806	116	430.9	50.0%	3	R.IVDEVAIIASK.R	11
	3264plusp804-02.11208.11208.2	4.5979	0.3882	1158.4521	1158.3806	1	1246.8	90.0%	13	R.IVDEVAIIASK.R	22
*	3264plusp804-02.09816.09816.1	2.2396	0.2262	1147.55	1148.3042	8	283.5	61.1%	1	K.SLGYDQIPVR.V	1
*	3264plusp804-02.10233.10233.2	3.6639	0.2419	1148.3922	1148.3042	1	1215.5	88.9%	19	K.SLGYDQIPVR.V	2

Similarities: SPCC24B10.09(3:2)

U **SPAC18G6.14c** 3 35 22.6% 195 21947 10.1 |rps7||40S ribosomal protein S7|Schizosaccharomyces pombe|chr 1|||Manual

	Filename	XCorr	DeltCN	ObsM+H+	CalcM+H+	SpR	SpScore	Ion%	#	Sequence	
*	3264plusp804-05.00700.00700.2	3.0531	0.3109	1324.8722	1324.6927	4	512.8	54.5%	15	K.AIVVFVPQPLLK.A	2
*	3264plusp804-02.11631.11631.2	3.7304	0.3577	1671.3722	1670.8192	1	673.7	69.2%	19	K.VFLDNRDANTVDYK.L	2
*	3264plusp804-02.16899.16899.2	2.6022	0.1308	1882.8722	1881.1357	1	872.8	52.9%	1	K.LTGKNVTFEFPVATGEGL.-	2

U **SPAC22A12.15c** 12 30 22.2% 663 73227 5.0 |bip1|bip|BiP |Schizosaccharomyces pombe|chr 1|||Manual

	Filename	XCorr	DeltCN	ObsM+H+	CalcM+H+	SpR	SpScore	Ion%	#	Sequence	
*	3264plusp804-02.11056.11056.2	4.104	0.5	1528.3522	1528.6586	1	792.1	75.0%	3	R.ITPSYVAFTEDER.L	2
*	3264plusp804-02.12497.12497.2	4.1616	0.3958	1689.2922	1688.8345	1	847.5	71.4%	2	K.NQAPSNPENTIFDIK.R	2
*	3264plusp804-02.18756.18756.2	4.1848	0.412	1466.5922	1466.7327	1	904.5	91.7%	3	K.FTPEEISAMILSK.M	2
*	3264plusp804-02.11837.11837.2	4.0107	0.4106	1200.0521	1200.3805	1	1705.7	90.9%	2	K.DAGTIAGLNVIR.I	2
*	3264plusp804-02.12649.12649.2	4.1436	0.5464	1646.0521	1646.881	1	1157.5	63.3%	1	R.IVNEPTAAAIAYGLDK.T	2
*	3264plusp804-02.12063.12063.2	5.1121	0.5769	2221.4722	2221.4692	1	1776.5	60.0%	1	R.IVNEPTAAAIAYGLDKTDTEK.H	2
*	3264plusp804-02.19159.19159.2	5.8398	0.5391	2119.652	2120.2798	1	2568.3	73.5%	7	R.IEIESFFNGQDFSETLSR.A	2
*	3264plusp804-02.11949.11949.2	2.9313	0.1522	1591.7522	1589.7863	1	1045.2	60.7%	2	K.KSEIDDIVLVGGSTR.I	2
*	3264plusp804-02.12578.12578.2	3.7194	0.4292	1461.7722	1461.6122	1	1273.6	73.1%	1	K.SEIDDIVLVGGSTR.I	2
*	3264plusp804-02.18108.18108.2	3.8841	0.3021	1297.6921	1297.4937	1	1276.3	80.0%	4	K.VQELLESFFGK.K	2
*	3264plusp804-02.17292.17292.2	2.7534	0.3386	1847.8121	1846.1896	1	550.8	52.9%	1	L.DVIPLTLGIETTGGVMTK.L	2
*	3264plusp804-02.11248.11248.2	3.8408	0.3835	1316.3722	1316.4534	1	1261.1	85.0%	3	R.NTLENYAYSLK.G	2

U **SPBC405.07** 2 20 22.2% 99 11258 11.8 |rpl3602|rpl36-2, rpl36|60S ribosomal protein L36|Schizosaccharomyces pombe|chr 2|||Manual

U **SPCC970.05** 2 20 22.2% 99 11263 11.8 |rpl3601|rpl36-1|60S ribosomal protein L36|Schizosaccharomyces pombe|chr 3|||Manual

Filename	XCorr	DeltCN	ObsM+H+	CalcM+H+	SpR	SpScore	Ion%	#	Sequence	
3264plusp804-02.09763.09763.2	3.1516	0.3536	1138.8322	1139.2529	1	603.1	77.8%	13	R.EVAGFAPYER.R	2
3264plusp804-02.10888.10888.2	4.4171	0.3803	1361.9521	1362.523	1	2181.9	90.9%	7	K.IEELTSVIQSSR.L	2

U **SPAC3C7.12** 9 20 21.7% 461 52607 4.6 |tip1|noc1|CLIP170 family plus end tracking protein Tip1|Schizosaccharomyces pombe|chr 1|||Manual

	Filename	XCorr	DeltCN	ObsM+H+	CalcM+H+	SpR	SpScore	Ion%	#	Sequence	
*	3264plusp804-05.00802.00802.2	5.3289	0.5065	1633.7922	1634.9312	1	1493.5	85.7%	8	-.MFPLGSVVEVITGER.G	2
*	3264plusp804-05.00490.00490.2	4.7898	0.4843	1869.4521	1869.1675	1	1577.8	78.1%	3	K.GVYVGLELLPEFAEFGK.N	2
*	3264plusp804-02.13483.13483.1	2.0998	0.346	1023.76	1024.2047	1	358.5	68.8%	1	K.TGIFVPFDK.C	1
*	3264plusp804-02.13490.13490.2	2.4409	0.1213	1025.3522	1024.2047	1	432.3	75.0%	2	K.TGIFVPFDK.C	2
*	3264plusp804-02.11739.11739.2	6.1378	0.4895	2584.7322	2584.8555	1	1626.0	52.1%	1	R.LTNVSSSSNLSMNTISSTALTPTEK.I	2
*	3264plusp804-02.11751.11751.3	4.8536	0.4167	2585.0645	2584.8555	1	1171.0	34.4%	1	R.LTNVSSSSNLSMNTISSTALTPTEK.I	3
*	3264plusp804-02.10110.10110.2	2.9197	0.1736	1050.9922	1051.1846	5	1227.8	92.9%	1	R.IEDLLYER.Q	2
*	3264plusp804-02.14569.14569.2	3.3546	0.2516	1580.5721	1580.6915	1	1299.1	70.8%	2	K.SEDDLLFSLQQER.D	2
*	3264plusp804-02.13007.13007.2	4.9715	0.4385	1591.4321	1591.7612	1	2331.7	83.3%	1	R.DYALNQVEILQER.V	2

U **SPBP8B7.03c** 5 19 21.5% 363 39767 10.8 |rpl402|rpl4-2, rpl4|60S ribosomal protein L2|Schizosaccharomyces pombe|chr 2|||Manual

	Filename	XCorr	DeltCN	ObsM+H+	CalcM+H+	SpR	SpScore	Ion%	#	Sequence	
*2	3264plusp804-02.19135.19135.2	4.9113	0.4906	1697.4521	1697.9255	1	1677.5	80.0%	6	K.DGSVSSETIALPFVFK.A	22
	3264plusp804-02.20892.20892.2	4.4255	0.4162	2255.892	2256.5579	1	1139.0	65.8%	3	R.IEEIPEVPLVVDDAVQSFQK.T	22
*	3264plusp804-02.13935.13935.2	4.0072	0.5263	1458.7322	1458.6976	1	1046.1	73.1%	2	R.GPLVVFNEDAGIVK.A	2
	3264plusp804-02.16505.16505.2	5.0062	0.484	1866.6522	1867.0685	1	1935.6	86.7%	6	K.NYFLPENIISNADVTR.L	22
	3264plusp804-02.11897.11897.2	4.5265	0.2166	1361.4321	1359.5626	1	1573.4	90.9%	2	R.LINSDEIQSIVK.A	22

Similarities: SPBC1711.06(4:1)

U **SPBC1685.09** 4 10 21.4% 56 6658 10.7 |rps29||40S ribosomal protein S29|Schizosaccharomyces pombe|chr 2|||Manual

	Filename	XCorr	DeltCN	ObsM+H+	CalcM+H+	SpR	SpScore	Ion%	#	Sequence	
*	3264plusp804-02.09913.09913.1	2.4513	0.3323	1155.54	1156.2805	2	150.4	55.6%	6	R.EYANDIGFVK.Y	1
*	3264plusp804-02.09807.09807.2	2.4834	0.2483	1156.2722	1156.2805	2	639.5	72.2%	2	R.EYANDIGFVK.Y	2
*	3264plusp804-02.11922.11922.2	2.8495	0.3545	1318.9722	1319.4564	1	852.6	85.0%	1	R.EYANDIGFVKY.R	2
*	3264plusp804-02.11589.11589.2	2.8611	0.2916	1476.8722	1475.6439	2	316.6	54.5%	1	R.EYANDIGFVKYR.-	2

U **SPCC1739.01** 8 97 16.5% 547 57978 9.6 ||SPCC1906.05|zinc finger protein|Schizosaccharomyces pombe|chr 3|||Manual

	Filename	XCorr	DeltCN	ObsM+H+	CalcM+H+	SpR	SpScore	Ion%	#	Sequence	
*	3264plusp804-02.15561.15561.2	5.9344	0.4875	1987.2522	1987.2205	1	1886.6	66.7%	72	R.LTLDQSLGNLSLGSGINQR.R	2
*	3264plusp804-02.15293.15293.3	5.4506	0.3732	1987.6743	1987.2205	1	2346.7	48.6%	8	R.LTLDQSLGNLSLGSGINQR.R	3
*	3264plusp804-02.12791.12791.2	4.9984	0.5465	1873.3922	1874.061	1	1736.7	67.6%	1	L.TLDQSLGNLSLGSGINQR.R	2
*	3264plusp804-02.09965.09965.2	4.1905	0.5385	2294.0923	2294.495	1	843.5	50.0%	2	R.YVEGSGSMSTTPLATSVNNSYK.L	2
*	3264plusp804-02.10970.10970.2	2.8203	0.3601	1892.3722	1894.0453	1	385.8	44.1%	1	R.EEAVFSSPTTEGSRPVSL.A	2
*	3264plusp804-02.10630.10630.2	4.7107	0.4876	1965.4122	1965.1241	1	1327.1	61.1%	3	R.EEAVFSSPTTEGSRPVSLA.R	2
*	3264plusp804-02.11681.11681.2	2.919	0.3168	2121.5723	2121.3115	217	117.6	28.9%	9	R.EEAVFSSPTTEGSRPVSLAR.L	2
*	3264plusp804-02.05183.05183.3	3.9481	0.4229	2935.1042	2934.1003	1	355.9	24.1%	1	R.SDTASPETIAGLGDTKNDPVVSTNNSVSR.I	3

U **SPAC1071.07c** 1 18 12.3% 154 17634 10.4 |rps1502|rps15-2, rps15|40S ribosomal protein S15|Schizosaccharomyces pombe|chr 1|||Manual
U **SPCC1393.03** 1 18 12.4% 153 17519 10.4 |rps1501|rps15-1|40S ribosomal protein S15|Schizosaccharomyces pombe|chr 3|||Manual

Filename	XCorr	DeltCN	ObsM+H+	CalcM+H+	SpR	SpScore	Ion%	#	Sequence	
3264plusp804-05.00021.00021.2	6.1561	0.5334	2034.4122	2035.4677	1	2273.3	80.6%	18	R.NMIILPEMVGSVVGIYNGK.L	2

U **SPAC664.11** 4 8 10.1% 674 72977 7.2 |ssc1|ssp1|heat shock protein 70 family|Schizosaccharomyces pombe|chr 1|||Manual

	Filename	XCorr	DeltCN	ObsM+H+	CalcM+H+	SpR	SpScore	Ion%	#	Sequence	
*	3264plusp804-02.11634.11634.2	2.7462	0.1645	1681.4122	1681.8455	1	343.7	53.6%	1	K.NAVVTVPAYFNDSQR.Q	2
*	3264plusp804-02.16270.16270.2	3.7909	0.2355	1648.5322	1647.8657	1	559.0	63.3%	2	K.TDISLPFITADATGPK.H	2
*	3264plusp804-02.16880.16880.2	3.7924	0.4551	2160.632	2161.435	1	1169.4	55.3%	1	K.DANLQTSEINEVILVGGMTR.M	2
*	3264plusp804-02.09747.09747.2	5.6409	0.564	1781.3522	1780.9323	1	1347.7	71.9%	4	K.SQVFSTAADGQTAVEIR.V	2

U **SPBC337.08c** 1 20 4.2% 382 42972 7.6 |ubi4||ubiquitin family protein|Schizosaccharomyces pombe|chr 2|||Manual

Filename	XCorr	DeltCN	ObsM+H+	CalcM+H+	SpR	SpScore	Ion%	#	Sequence	
3264plusp804-02.10703.10703.2	4.236	0.4835	1764.5322	1764.9244	1	1384.8	66.7%	20	K.TITLEVESSDTIDNVK.S	2

U **SPBC23E6.08** 1 24 3.8% 550 62618 5.8 |sat1||involved in G1 phase arrest |Schizosaccharomyces pombe|chr 2|||Manual

	Filename	XCorr	DeltCN	ObsM+H+	CalcM+H+	SpR	SpScore	Ion%	#	Sequence	
*	3264plusp804-02.14352.14352.2	4.0304	0.2428	2226.0923	2227.5835	1	1198.0	50.0%	24	S.SYSVINTLNGMIGNLFGVKQA.S	2

U **SPCC970.10c** 2 16 3.2% 680 78053 6.3 |||zinc finger protein|Schizosaccharomyces pombe|chr 3|||Manual

	Filename	XCorr	DeltCN	ObsM+H+	CalcM+H+	SpR	SpScore	Ion%	#	Sequence	
*	3264plusp804-02.13682.13682.3	4.4772	0.0976	2213.3044	2212.4185	1	1671.0	40.0%	15	L.LSTVDSSTNSVSRDPFSVLSI.D	3
*	3264plusp804-02.14123.14123.2	3.0882	0.1744	1928.8522	1927.0319	3	667.8	47.1%	1	V.DSSTNSVSRDPFSVLSID.D	2

U **SPBPB2B2.06c** 1 42 3.2% 601 68418 5.1 |||calcineurin-like phosphoesterase|Schizosaccharomyces pombe|chr 2|||Manual

	Filename	XCorr	DeltCN	ObsM+H+	CalcM+H+	SpR	SpScore	Ion%	#	Sequence	
*	3264plusp804-02.19065.19065.2	4.1405	0.2747	2211.412	2213.446	1	1007.2	50.0%	42	S.EDSFDTPEGIELSKIIKQY.R	2

U **SPAC13C5.02** 1 11 2.7% 411 48519 5.7 |dre4||WW domain |Schizosaccharomyces pombe|chr 1|||Manual

	Filename	XCorr	DeltCN	ObsM+H+	CalcM+H+	SpR	SpScore	Ion%	#	Sequence	
*	3264plusp804-02.10044.10044.2	2.6489	0.156	1197.4922	1196.3024	2	502.6	60.0%	11	S.QASLAFNTSEK.L	2

U **SPAC31A2.07c** 2 64 1.9% 848 94661 9.5 |||DEAD/DEAH box helicase|Schizosaccharomyces pombe|chr 1|||Manual

	Filename	XCorr	DeltCN	ObsM+H+	CalcM+H+	SpR	SpScore	Ion%	#	Sequence	
*	3264plusp804-02.13042.13042.1	2.583	0.1845	1795.78	1795.9469	47	109.4	36.7%	1	A.ARHAILDLTNDEGIEQ.S	1
*	3264plusp804-02.20206.20206.2	2.9931	0.1441	1796.3322	1795.9469	12	281.4	40.0%	63	A.ARHAILDLTNDEGIEQ.S	2

U **SPAC1782.01** 1 21 0.9% 1679 190967 6.1 ||SPAPYUG7.07|conserved eukaryotic protein|Schizosaccharomyces pombe|chr 1|||Manual

	Filename	XCorr	DeltCN	ObsM+H+	CalcM+H+	SpR	SpScore	Ion%	#	Sequence	
*	3264plusp804-01.17508.17508.2	3.1931	0.1156	1683.1522	1680.9539	19	595.2	50.0%	21	I.GLLDESSMQTLYPIL.N	2

	Proteins	Peptide IDs	Copies
Unfiltered	4665	40576	43247
Redundant	29	166	950
Nonredundant	26	149	886
