# Supplementary material for: Ubiquitination of CLIP-170 family protein restrains polarized growth upon DNA replication stress
Source: Nat Commun. 2022 Sep 22;13:5565. doi: 10.1038/s41467-022-33311-y (PMC9499959; doi:10.1038/s41467-022-33311-y)
Supplement: Supplementary file 5 — Reporting Summary [file 41467_2022_33311_MOESM5_ESM.pdf]

nature portfolio

Corresponding author(s): Chuanhai Fu, Yamei Wang, Quan-wen Jin

Last updated by author(s): 2022/08/23

# Reporting Summary

Nature Portfolio wishes to improve the reproducibility of the work that we publish. This form provides structure for consistency and transparency in reporting. For further information on Nature Portfolio policies, see our Editorial Policies and the Editorial Policy Checklist.

## Statistics

For all statistical analyses, confirm that the following items are present in the figure legend, table legend, main text, or Methods section.

| n/a | Confirmed | |
|---|---|---|
| ☐ | ☒ | The exact sample size (*n*) for each experimental group/condition, given as a discrete number and unit of measurement |
| ☐ | ☒ | A statement on whether measurements were taken from distinct samples or whether the same sample was measured repeatedly |
| ☐ | ☒ | The statistical test(s) used AND whether they are one- or two-sided<br>*Only common tests should be described solely by name; describe more complex techniques in the Methods section.* |
| ☒ | ☐ | A description of all covariates tested |
| ☒ | ☐ | A description of any assumptions or corrections, such as tests of normality and adjustment for multiple comparisons |
| ☐ | ☒ | A full description of the statistical parameters including central tendency (e.g. means) or other basic estimates (e.g. regression coefficient) AND variation (e.g. standard deviation) or associated estimates of uncertainty (e.g. confidence intervals) |
| ☐ | ☒ | For null hypothesis testing, the test statistic (e.g. *F*, *t*, *r*) with confidence intervals, effect sizes, degrees of freedom and *P* value noted<br>*Give P values as exact values whenever suitable.* |
| ☒ | ☐ | For Bayesian analysis, information on the choice of priors and Markov chain Monte Carlo settings |
| ☒ | ☐ | For hierarchical and complex designs, identification of the appropriate level for tests and full reporting of outcomes |
| ☒ | ☐ | Estimates of effect sizes (e.g. Cohen's *d*, Pearson's *r*), indicating how they were calculated |

*Our web collection on statistics for biologists contains articles on many of the points above.*

## Software and code

Policy information about availability of computer code

| | |
|---|---|
| Data collection | Image collection was performed with Element software (Nikon, version 2.34), Metamorph software (Molecular Devices, version 7.1) or iQ software (Andor, version 3.6). Scansifter software (v2.1.25) was used to extract Mass Spectrometry spectra; SEQUEST algorithm (TurboSequest, v.2.7) was used to search Mass Spectrometry spectra against the S. pombe protein database; DTASelect software (v1.9) was used to assemble and filter peptides. |
| Data analysis | Statistical analyses were performed with Prism (GraphPad Software Inc., versions 6.0) or Microsoft Office Excel (Microsoft, version 2017); flow cytometry data were analyzed with FlowJo (version 10) software; images were analyzed with Fiji/ImageJ (National Institutes of Health, open source, versions 2.0.0-rc-69). |

For manuscripts utilizing custom algorithms or software that are central to the research but not yet described in published literature, software must be made available to editors and reviewers. We strongly encourage code deposition in a community repository (e.g. GitHub). See the Nature Portfolio guidelines for submitting code & software for further information.

## Data

Policy information about availability of data

All manuscripts must include a data availability statement. This statement should provide the following information, where applicable:

- Accession codes, unique identifiers, or web links for publicly available datasets
- A description of any restrictions on data availability
- For clinical datasets or third party data, please ensure that the statement adheres to our policy

The list of potential Dma1-interacting proteins identified by mass spectrometry in this study is provided in the Supplementary Data.

# Field-specific reporting

Please select the one below that is the best fit for your research. If you are not sure, read the appropriate sections before making your selection.

☒ Life sciences ☐ Behavioural & social sciences ☐ Ecological, evolutionary & environmental sciences

For a reference copy of the document with all sections, see nature.com/documents/nr-reporting-summary-flat.pdf

# Life sciences study design

All studies must disclose on these points even when the disclosure is negative.

| | |
|---|---|
| Sample size | Sample size was as large as practicable for all experiments. |
| Data exclusions | No data were excluded from our studies. |
| Replication | All experiments were reproduced reliably two to more than three times in independent setting with similar results obtained. |
| Randomization | Randomization is built in to all of our experiments in fission yeast, as colonies are chosen randomly for outgrowth. |
| Blinding | Investigators were blinded to group allocation during experiments and not blinded to data analysis. Blinding was unecessary for data analysis as phenotypes were objectively obvious/large, and would be impossible as the phenotype automatically reports on the genotype upon inspection. |

# Reporting for specific materials, systems and methods

We require information from authors about some types of materials, experimental systems and methods used in many studies. Here, indicate whether each material, system or method listed is relevant to your study. If you are not sure if a list item applies to your research, read the appropriate section before selecting a response.

## Materials & experimental systems

| n/a | Involved in the study | |
|---|---|---|
| ☐ | ☒ | Antibodies |
| ☒ | ☐ | Eukaryotic cell lines |
| ☒ | ☐ | Palaeontology and archaeology |
| ☒ | ☐ | Animals and other organisms |
| ☒ | ☐ | Human research participants |
| ☒ | ☐ | Clinical data |
| ☒ | ☐ | Dual use research of concern |

## Methods

| n/a | Involved in the study | |
|---|---|---|
| ☒ | ☐ | ChIP-seq |
| ☒ | ☐ | Flow cytometry |
| ☒ | ☐ | MRI-based neuroimaging |

## Antibodies

| | |
|---|---|
| Antibodies used | Primary antibodies used were peroxidase-anti-peroxidase (PAP) soluble complex (Sigma-Aldrich; P1291); rabbit polyclonal anti-Myc (GeneScript; A00172-40); mouse monoclonal anti-GFP (clone 7.1/13.1; Roche, Cat. No. 11814460001); mouse monoclonal anti-GFP (clone ME11; Beijing Ray Antibody Biotech; RM1008); rat monoclonal anti-HA (clone 3F10; Roche, Cat. No. 11 867 423 001); mouse monoclonal anti-Cig2 (clone 3A11/5; Santa Cruz Biotechnology, sc-53223); rabbit polyclonal anti-PSTAIRE (detecting Cdc2) (Santa Cruz Biotechnology; sc-53).<br>Secondary antibodies used were goat anti-mouse or goat anti-rabbit polyclonal IgG (H+L) HRP conjugates (Thermo Fisher Scientific; #31430 or #32460). |
| Validation | 1. Peroxidase-anti-peroxidase (PAP) soluble complex (Sigma-Aldrich; P1291) was used and thus validated to detect TAP-tagged fission yeast proteins in previous publications, such as: DOI 10.1016/j.cub.2011.12.049, and DOI: 10.1016/j.molcel.2017.04.017.<br>2. Rabbit polyclonal anti-Myc (GeneScript; A00172-40) is validated by manufacturer using c-Myc tagged fusion proteins (https://www.genscript.com/antibody/A00172-c_Myc_tag_Antibody_pAb_Rabbit.html).<br>3. Mouse monoclonal anti-GFP (clone 7.1/13.1; Roche, Cat. No. 11814460001) is validated by manufacturer (https://www.sigmaaldrich.cn/deepweb/assets/sigmaaldrich/product/documents/294/951/11814460001bul.pdf), and was also used and thus validated to immunoprecipitate and detect GFP-tagged fission yeast proteins in previous publications, such as: DOI: 10.1016/j.cub.2017.03.007.<br>4. Mouse monoclonal anti-GFP (clone ME11; Beijing Ray Antibody Biotech, RM1008) is validated by manufacturer with purified GFP (http://www.rayantibody.com/uploads/2014/07/101640111509.pdf).<br>5. Rat monoclonal anti-HA (clone 3F10; Roche, Cat. No. 11 867 423 001) is validated by manufacturer (https://www.sigmaaldrich.cn/deepweb/assets/sigmaaldrich/product/documents/248/175/roahahabul.pdf).<br>6. Mouse monoclonal anti-Cig2 (clone 3A11/5; Santa Cruz Biotechnology, sc-53223) is validated by manufacturer (https://datasheets.scbt.com/sc-53223.pdf), and was also used and thus validated to detect fission yeast Cig2 proteins in previous publications, such as: DOI:10.1111/febs.16138. |

7. Rabbit polyclonal anti-PSTAIRE (detecting Cdc2) (Santa Cruz Biotechnology; sc-53) is validated by manufacturer (https://www.scbt.com/zh/p/cig2-antibody-cig2-3a11-5), and has been used regularly in our previous studies, such as: DOI: 10.1242/jcs.132845.
8. Goat anti-mouse polyclonal IgG (H+L) HRP conjugates (Thermo Fisher Scientific; #31430 or #32460) is validated by manufacturer (https://www.thermofisher.cn/cn/zh/antibody/product/Goat-anti-Mouse-IgG-H-L-Secondary-Antibody-Polyclonal/31430).
9. Goat anti-rabbit polyclonal IgG (H+L) HRP conjugates (Thermo Fisher Scientific; #31430 or #32460) is validated by manufacturer (https://www.thermofisher.cn/cn/zh/antibody/product/Goat-anti-Rabbit-IgG-H-L-Secondary-Antibody-Polyclonal/32460).
